# Base editing of trinucleotide repeats that cause Huntington’s disease and Friedreich’s ataxia reduces somatic repeat expansions in patient cells and in mice

**DOI:** 10.1038/s41588-025-02172-8

**Published:** 2025-05-26

**Authors:** Zaneta Matuszek, Mandana Arbab, Maheswaran Kesavan, Alvin Hsu, Jennie C. L. Roy, Jing Zhao, Tian Yu, Ben Weisburd, Gregory A. Newby, Neil J. Doherty, Muzhou Wu, Shota Shibata, Ana Cristian, Y. Allen Tao, Liam G. Fearnley, Melanie Bahlo, Heidi L. Rehm, Jun Xie, Guangping Gao, Ricardo Mouro Pinto, David R. Liu

**Affiliations:** 1 Merkin Institute of Transformative Technologies in Healthcare, Broad Institute of Harvard and MIT, Cambridge, MA USA; 2 Department of Chemistry and Chemical Biology, Harvard University, Cambridge, MA USA; 3 Department of Molecular and Cellular Biology, Harvard University, Cambridge, MA USA; 4 Department of Neurology, Harvard Medical School, Boston, MA USA; 5 FM Kirby Neurobiology Center, Boston Children’s Hospital, Boston, MA USA; 6 Center for Genomic Medicine, Massachusetts General Hospital, Boston, MA USA; 7 Molecular Medicine Program, Faculty of Medicine, Laval University, Quebec City, Quebec Canada; 8 Rosamund Stone Zander Translational Neuroscience Center, Department of Neurology, Boston Children’s Hospital, Boston, MA USA; 9 Division of Genetics, Department of Medicine, Brigham and Women’s Hospital and Harvard Medical School, Boston, MA USA; 10 Program in Medical and Population Genetics, Broad Institute of MIT and Harvard, Cambridge, MA USA; 11 Department of Genetic Medicine, The Johns Hopkins University, Baltimore, MD USA; 12 Population Health and Immunity Division, The Walter and Eliza Hall Institute of Medical Research, Parkville, Victoria Australia; 13 Department of Medical Biology, The University of Melbourne, Parkville, Victoria Australia; 14 Horae Gene Therapy Center, University of Massachusetts Medical School, Worcester, MA USA; 15 Microbiology and Physiological Systems, University of Massachusetts Medical School, Worcester, MA USA; 16 Howard Hughes Medical Institute, Harvard University, Cambridge, MA USA; 17 Present Address: Rosamund Stone Zander Translational Neuroscience Center, Department of Neurology, Boston Children’s Hospital, Boston, MA USA; 18 Present Address: Department of Neurology, Harvard Medical School, Boston, MA USA

**Keywords:** Targeted gene repair, Targeted gene repair

## Abstract

Trinucleotide repeat (TNR) diseases are neurological disorders caused by expanded genomic TNRs that become unstable in a length-dependent manner. The CAG•CTG sequence is found in approximately one-third of pathogenic TNR loci, including the *HTT* gene that causes Huntington’s disease. Friedreich’s ataxia, the most prevalent hereditary ataxia, results from GAA repeat expansion at the *FXN* gene. Here we used cytosine and adenine base editing to reduce the repetitiveness of TNRs in patient cells and in mice. Base editors introduced G•C>A•T and A•T>G•C interruptions at CAG and GAA repeats, mimicking stable, nonpathogenic alleles that naturally occur in people. AAV9 delivery of optimized base editors in *Htt.Q111* Huntington’s disease and YG8s Friedreich’s ataxia mice resulted in efficient editing in transduced tissues, and significantly reduced repeat expansion in the central nervous system. These findings demonstrate that introducing interruptions in pathogenic TNRs can mitigate a key neurological feature of TNR diseases in vivo.

## Abstract (web summary)

Base editing of the pathogenic trinucleotide repeat expansions underlying Huntington’s disease and Friedreich’s ataxia introduces repeat interruptions that reduce somatic expansion in patient cells and mice.

## Main

Trinucleotide repeat (TNR) sequences are common genomic elements that can become unstable in a length-dependent manner. Pathogenic expansion of TNRs is associated with over 40 severe, predominantly neurological disorders^1^. TNRs may be localized to gene promoters, coding sequences, untranslated regions and introns, and the repeat motif can vary between TNR disorders^2^. The most common pathogenic triplet base pair is CAG•CTG, which occurs in at least 15 known pathogenic TNR loci^2^. CAG repeats in exons frequently encode oligomers of glutamine^3^. These polyglutamine (poly-Q) diseases include Huntington’s disease (HD), spinocerebellar ataxias (SCAs), dentatorubral-pallidoluysian atrophy, and spinal and bulbar muscular atrophy. The most common hereditary ataxia in humans, Friedreich’s ataxia (FRDA), is caused by the intronic expansion of GAA repeats. Currently there are no approved treatments that halt TNR disease progression^4–6^.

The age of TNR disease onset, disease severity and rate of disease progression are primarily determined by the length of the corresponding repeat tract at birth, with longer repeats being associated with a less favorable prognosis. Repeat lengths beyond a locus-specific threshold are unstable in some somatic cells and can expand, contract and become increasingly unstable as repeat length increases. The instability of these genomic loci results from the formation of higher-order DNA and R-loop structures during transcription and cell replication that interfere with normal cell function^7–10^. These abnormal structures are subjected to error-prone DNA repair that can result in the expansion or contraction of the repeat, with a general bias toward expansions in longer repeat tracts^11–14^. Single-cell analysis of brain tissue from patients with HD suggests that affected neurons undergo decades of CAG repeat expansion without evident phenotype before crossing a threshold that causes rapid neurodegeneration^15^, suggesting that therapeutic intervention to halt somatic repeat expansion before this threshold is reached may prevent or impede disease onset or progression.

In cell and animal models, naturally occurring single-nucleotide variants within repeat tracts inhibit the formation of higher-order nucleotide structures and reduce repeat instability^16–22^. At various TNR loci, repeat instability is better predicted by the length of pure uninterrupted repeats than by the length of the repeat tract^13,23^. TNR interruptions such as synonymous CAA triplets within CAG repeat tracts, or GAG or GGA triplets within GAA repeat tracts, are common. TNR interruptions in patients are associated with reduced somatic instability^24–26^, reduced transgenerational transmission^27–29^, delayed onset and progression of the disease, and overall milder phenotypes compared with individuals with uninterrupted repeats^13,26,30–41^. Genome-wide association data from >9,000 patients with HD suggest that a single CAA interruption in a CAG poly-Q region delays HD onset by an average of 12 yr (refs. ^13,22^), while pedigree analyses of 16 patients with HD suggest that CAG repeat interruption delays disease onset 13–29 yr (refs. ^36,42^). These findings collectively raise the possibility that introducing interruptions in pathogenic TNR tracts might improve their genomic stability and ameliorate disease pathology.

Base editing is a precision genome editing technology that directly introduces targeted changes to the DNA in living cells^43–48^. Cytosine base editors (CBEs), which mediate C•G>T•A substitutions, and adenine base editors (ABEs), which mediate A•T>G•C substitutions, in theory can install single-nucleotide changes that interrupt repeats within TNR alleles resembling interruptions found in the general population, or in mild or unaffected individuals with long repeats^23^^,^^49^ (Table 1 and Supplementary Table 1). In this study, we use base editing to introduce interruptions in repeats associated with HD and FRDA and assess their effect on expansion of these repeats in patient cells and in mouse models of these diseases.

## Results

### Synonymous cytosine base editing of CAG repeats in vitro

**Fig. 1:**
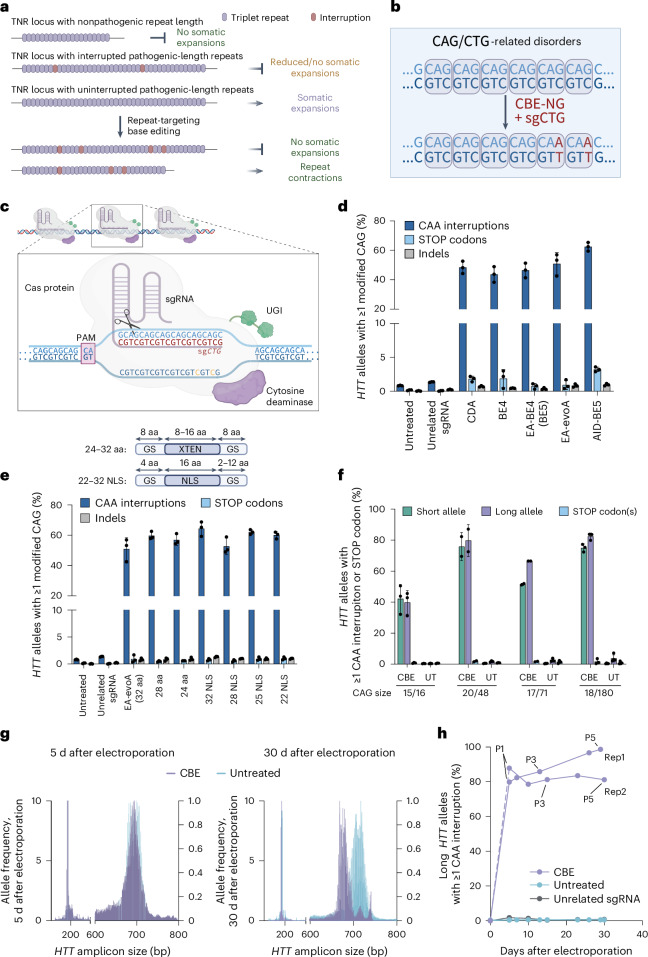
Synonymous cytosine base editing of CAG repeats in vitro*.* **a**, An overview of the base editing approach to reduce triplet-repeat expansions. **b**, Schematic of the CAG-CBE base editing strategy. **c**, An illustration of cytosine base editing at CAG repeats. The smaller cartoon illustrates the multiple binding opportunities for the Cas9-sgCTG complex at CAG repeats. The magnified snippet shows a singular binding event. **d**, Optimization of cytosine base editing strategies in HEK293T cells. Data are mean ± s.d. of biological triplicates. **e**, Optimization of the ‘GS’ linker of EA-evoA-Cas9-NG in HEK293T cells. Data are mean ± s.d. of biological triplicates. **f**, CAG repeat base editing at *HTT* alleles in human fibroblasts. Numbers below the bars indicate the number of CAG repeats (CAG size) in *HTT* alleles. Data are mean ± s.d. of biological replicates (*n* = 2 for HD cell lines with 20/48 and 17/71 CAGs, *n* = 3 for HD cell lines with 15/16 and 18/180 CAGs). **g**, Distribution of *HTT* CAG allele sizes in CBE-treated (CBE) and untreated HD fibroblasts with 18/180 CAG repeats in Rep1, 5 d (P1) and 30 d (P5) after electroporation, as measured by fragment analysis. **h**, CAG repeat base editing in HD fibroblasts with 18/180 CAG repeats measured across 30 d and five cell passages. P1–P5 refer to cell passages 1–5. Rep1 and Rep2 refer to two independent biological replicates. Illustrations in **a**, **c** and **e** were created using BioRender.com. CBE, CBE-treated; UGI, uracil DNA glycosylase inhibitor domain; UT, untreated cells. Source data

In patients suffering from poly-Q disorders including HD and SCAs, naturally occurring synonymous CAA interruptions in pathogenic CAG repeats are associated with a delayed or lack of disease onset^13,22,36,42,50^. These interruptions are proposed to stabilize repeats and suppress somatic repeat expansion^17,18,22^. In HD knock-in mice, long stretches of alternating CAG and CAA codons do not undergo somatic expansion, unlike pure CAG repeats of similar length^22^. We hypothesized that introducing CAA interruptions throughout CAG repeat tracts by cytosine base editing might reduce the expansion of long pathogenic TNR alleles (Fig. 1a).

**Extended Data Fig. 1:**
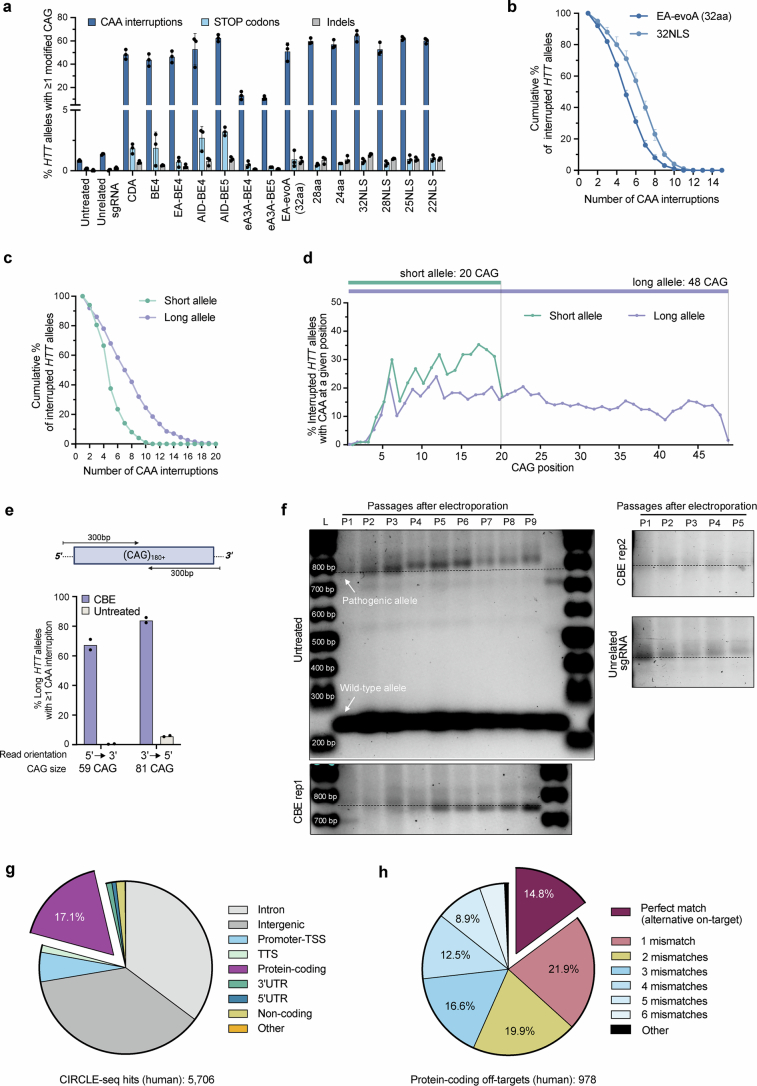
Synonymous cytosine base editing of CAG repeats in vitro. (**a**) Optimization of cytosine base editing strategies in HEK293T cells. Data are mean ± SD of biological triplicates. (**b-c**) Cumulative mean % of interrupted *HTT* alleles with at least a specified number of CAA interruptions induced by EA-evoA-NG in (**b**) HEK293T and (**c**) HD fibroblasts (GM04855, 20/48 CAGs). Data are mean ± SD of biological replicates ((**b**) n = 3, (**c**) n = 2). (**d**) Distribution of CAA interruptions throughout CAG repeats in GM04855 fibroblasts (20/48 CAGs) at interrupted *HTT* alleles represented as % interrupted alleles with CAA interruptions at a given position of the repeat tract. Data are mean ± SD of two biological replicates. (**e**) CAG repeat base editing at the pathogenic *HTT* allele in HD fibroblasts with 180 CAG repeats (GM09197) quantified in 5’- > 3’ (spanning 59 CAGs) and 3′- > 5′ (spanning 81 CAGs) sequencing direction. Data are mean ± SD of two biological replicates. (**f**) A representative agarose gel showing the distribution of CAG allele sizes in CBE-treated (CBE) and untreated HD fibroblasts with 180 CAG repeats (GM09197); P – passage, L – ladder. The dashed lines indicate the starting CAG size. The experiment was performed twice with similar results. (**g-h**) CIRCLE-seq nominated off-target hits in the human genome classified by (**g**) the identity of the targeted region annotated with HOMER and (**h**) number of mismatches of the protein-coding sites with the sgCTG. TSS-transcription start site, TTS-transcription termination site. Source data

To induce CAA interruptions in CAG repeat tracts, we designed a single-guide RNA (sgRNA) targeting CTG repeats on the opposite strand (sgCTG) and compared base editing throughout the repeat region using eight cytosine deaminases in the BE4max architecture with the NG-protospacer adjacent motif (PAM) Cas9 variant^42,43^ (Fig. 1b–d and Extended Data Fig. 1a). We co-transfected HEK293T cells, which contain an average of 17 CAG repeats at *HTT*, with plasmids encoding a CBE and sgCTG. We measured edited repeat tracts at the *HTT* locus by Illumina high-throughput sequencing (HTS) and an in-house software, powTNRka (Supplementary Note 2). Even though CAG repeats are common genetic elements and thus sgCTG should target many sites in the human genome, we did not observe any evident cellular toxicity. We determined the fraction of *HTT* alleles with at least one CAA interruption within the sequenced *HTT* CAG repeat tract of 17 repeats, and observed 44–62% average editing among our top six strategies (48 ± 4.2% for CDA-BE4 (ref. ^51^), 44 ± 5.3% for BE4 (refs. ^52,53^), 46 ± 5.0% for EA-BE4 (ref. ^54^), 51 ± 7.5% for EA-evoA (refs. ^51,54^), 53 ± 13% for AID-BE4 (ref. ^55^) and 62 ± 3.0% for AID-BE5 (ref. ^54^)) (Fig. 1d and Extended Data Fig. 1a).

Rarely, CBEs induce G•C>A•T changes upstream of the sgRNA binding site^54^ on the opposing DNA strand (Supplementary Text). This effect is more likely when multiple CBE binding events occur in proximity at the same target site, as with our sgCTG-targeting approach^54^. At glutamine-coding CAG repeats these edits can result in nonsense mutation (CAG to TAG or TAA). rAPOBEC1 family deaminases that harbor the ‘EA’ purity and efficiency modifications achieved the highest top-strand product purity^54^ (55:1 for EA-evoA and 63:1 for EA-BE4; Fig. 1d and Extended Data Fig. 1a). Modifying the flexible Gly-Ser (GS) linker between the EA-evoA deaminase and Cas protein improved editing outcomes: rigid linkers incorporating a nuclear localization signal (NLS)^53,56^ enhanced editing efficiency by up to 1.3-fold and product purity by 1.6-fold (Fig. 1e, Supplementary Text and Extended Data Fig. 1a,b). The EA-evoA-32NLS base editor yielded the highest editing efficiency (64 ± 4.8%) as well as the highest top-strand purity (81:1). We selected this editing strategy (hereafter designated CAG-CBE) for further study.

### CBE interruption of HTT CAG repeats reduces expansion in HD cells

To assess genome editing by CAG-CBE in pathogenic CAG repeats, we quantified interruptions in three HD patient-derived fibroblast lines that each carry one wild-type *HTT* allele and one pathogenic allele with 48–180 CAG repeats (Methods). We delivered the base editor and synthetic sgCTG by messenger RNA electroporation^47,57^. At 5 d after electroporation we observed that 66–82% of treated cells contained interrupted repeats in the pathogenic CAG repeat tract (Fig. 1f). Within each sample, editing was ~1.1–1.3-fold higher at the long pathogenic *HTT* allele than at the shorter wild-type allele (Fig. 1f), suggesting that the increase in binding opportunities for the CBE at long pathogenic alleles results in higher targeting efficiency and a greater number of interruptions per allele (Extended Data Fig. 1c–e and Supplementary Text).

Next, we asked whether CAG-CBE-induced repeat interruptions affect the stability of edited *HTT* alleles. We cultured GM09197 primary patient fibroblasts for up to 30 d until the cell lines reached senescence, and observed progressive expansion of the pathogenic *HTT* allele that initially contained 180 CAG repeats on average. We assessed CAG repeat instability at five consecutive passages by measuring the CAG repeat length in edited versus unedited cells (Fig. 1g and Extended Data Fig. 1f). Base editing of the *HTT* alleles was completed by 5 d after electroporation and was durable throughout the experiment. We observed a modest increase in the fraction of edited cells at later timepoints, likely due to stochastic clonal expansion within the bulk cell culture, or possibly a growth advantage of the edited fibroblasts relative to unedited cells (Fig. 1h). By passage 5 (30 d after treatment), we observed a shift in the CAG repeat distribution in untreated and mock-edited cells toward an increased repeat size, with the most frequent CAG allele acquiring ~6 CAG repeats relative to passage 1 (day 5 posttreatment; Fig. 1g and Extended Data Fig. 1f). In contrast, CAG-CBE-treated HD fibroblasts did not exhibit repeat expansion by passage 5, and the most frequent CAG allele was reduced by ~5 CAG repeats compared with passage 1. These findings demonstrate that inducing interruptions in pathogenic-length CAG repeats by base editing can prevent somatic repeat expansion and promote contraction of the pathogenic repeats.

### Genome-wide off-target editing analysis of CAG repeat base editing

**Fig. 2:**
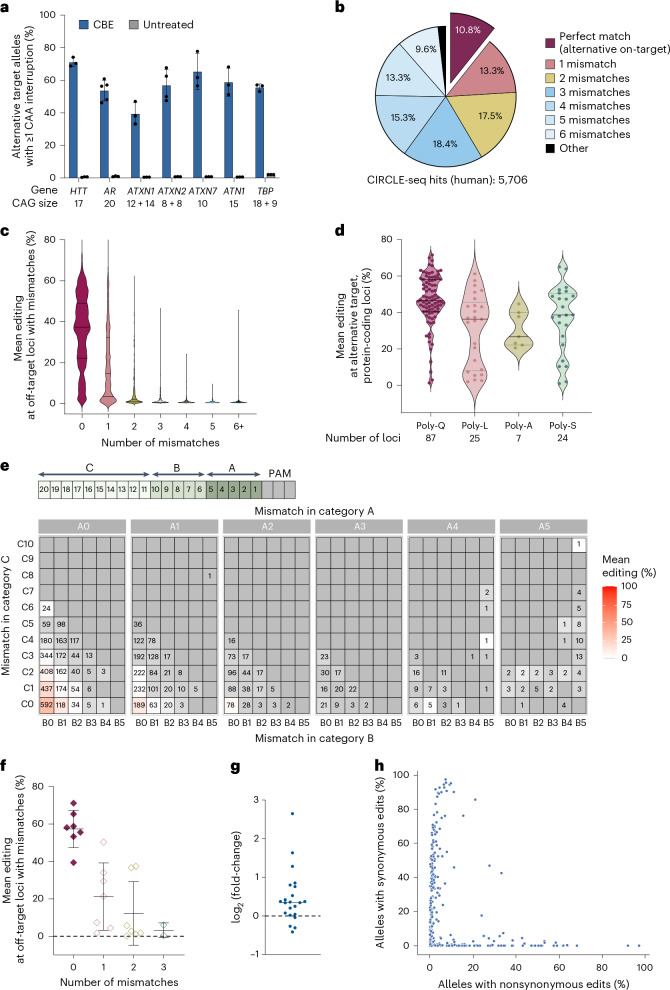
Alternative target and off-target editing analysis of CAG-CBE strategy. **a**, Base editing at TNR disease-associated genes in HEK293T cells. Data are mean ± s.d. of biological triplicates, except for *AR* (*n* = 5) and *ATXN2* (*n* = 4). **b**, CIRCLE-seq off-target hits in the human genome classified by the number of mismatches with sgCTG. **c**–**e**, Alternative target and off-target editing at CIRCLE-seq sites in HEK293T cells, quantified by WGS. **c**, Violin plot representing mean base editing frequencies at CIRCLE-seq sites (>0.5% editing by WGS), classified by mismatch number. Median and quartiles are shown. **d**, Alternative target editing at protein-coding sites in HEK293T cells, grouped by encoded amino acids. Median and quartiles are shown; each dot represents mean editing at a specific locus. **e**, Base editing at CIRCLE-seq sites (>0.5% editing by WGS) and grouped by mismatch position relative to sgCTG spacer. Mean editing (%) represents base editing frequency across genomic sites meeting the specified mismatch criteria. Mismatch category A includes the five nucleotides closest to the PAM (positions 1–5), category B represents positions 6–10 and category C spans the last ten, PAM-distal nucleotides (positions 11–20) of the protospacer. A0–A5, B0–B5 and C0–C10 indicate the number of mismatches (0–5) between the sgCTG and a target site in categories A, B and C. Each square shows the number of loci with >0.5% editing in each mismatch subgroup. **f**, Editing at protein-coding sites with 0–3 mismatches between the sgCTG and a target site in HEK293T cells, measured by HTS. Each dot represents mean editing at a unique locus; disease-associated genes are colored diamonds. Data are mean ± s.d. of all loci in each category. **g**, Comparison of editing quantified by WGS and HTS at selected sites. Each dot represents the log_2_ fold-change between editing frequencies quantified by WGS and HTS at a single locus, with the horizontal line indicating median (*n* = 22, *P* = 0.0045, one-sample *t*-test and Wilcoxon test). **h**, Impact of CAG repeat editing on amino acid sequence at protein-coding genes. The scatterplot shows the percentage of synonymous (*y* axis) and nonsynonymous (*x* axis) editing at edited alleles for each protein-coding gene. Each dot represents mean editing calculated for all loci mapping to a unique gene. Data in **c**–**h** represent biological triplicates. Poly-A, polyalanine; Poly-L, polyleucine; Poly-S, polyserine. Source data

Expansion of CAG repeats at various loci is associated with TNR diseases, including HD and several SCAs^2,58–61^. Our CAG-CBE strategy targets pure CAG•CTG repeats, enabling repeat interruption across pathogenic expansion loci regardless of gene identity. We observed that CAG-CBE introduces interruptions in 39–65% of alleles at multiple TNR loci (*AR*, *ATNX1*, *ATNX2*, *ATNX7*, *ATN1* and *TBP*; Fig. 2a), demonstrating its potential for interrupting and reducing CAG•CTG repeat expansions in a range of TNR disorders.

Base editors can induce off-target edits in the genome through Cas-dependent and Cas-independent mechanisms^62–67^. Many coding and noncoding sites in the human genome contain ≥8 CTG repeats that match the sgCTG spacer, including regions not known to be associated with disease^68^. To assess Cas-dependent off-target activity of CAG-CBE, we performed circularization for in vitro reporting of cleavage effects by sequencing (CIRCLE-seq) analysis on human genomic DNA (gDNA) from HEK293T cells using purified ribonucleoprotein complexes composed of Cas9-NG nuclease and sgCTG^69^. CIRCLE-seq nominated 5,706 potential targets, including 614 (10.8%) loci with ≥8 CTG repeats that perfectly match the sgCTG spacer (‘alternative targets’). These alternative targets include 16 TNR disease-associated loci, 129 additional protein-coding loci and 469 noncoding loci (Fig. 2b). The remaining >89% of CIRCLE-seq-nominated loci harbor mismatches to the sgCTG that are known to inhibit sgRNA binding and base editing in cells^70–72^ (Fig. 2b), including 833 protein-coding loci. We classified these hits based on: (1) the identity of the annotated targeted region with hypergeometric optimization of motif enrichment tool (HOMER)^73^; and (2) the number of mismatches with the pure CAG•CTG repeat targeted by sgCTG^70–72^ (Fig. 2b, Extended Data Fig. 1g,h and Supplementary Tables 2 and 3)^.^

To experimentally validate genome-wide off-target activity, we performed whole-genome sequencing (WGS) in HEK293T cells at a 160× average read depth, and quantified the fraction of CIRCLE-seq-nominated loci with at least one CAA interruption observed at >0.5% over background levels. We detected cytosine base editing at 48% of CIRCLE-seq-nominated loci (2,753), with 1,240 sites showing ≥5% editing (Supplementary Table 4). Editing was greatest at alternative targets (35 ± 18% across 579 loci; Fig. 2c), including 143 protein-coding loci. Most of these encode poly-Q or polyleucine (114 total) and are converted to synonymous codons by cytosine base editing (CAG-to-CAA and CTG-to-TTG, respectively; Fig. 2d). As anticipated, mismatches progressively reduced observed editing^70–72^; a single mismatch decreased editing by ~1.8-fold, while three or more mismatches greatly reduced or abolished off-target editing^70–72^ (Fig. 2c–e and Supplementary Text).

To validate our WGS results^54,64^, we quantified CBE-induced C•G>T•A interruptions at seven poly-Q-coding alternative targets and 16 CIRCLE-seq-nominated protein-coding off-targets using targeted-amplicon HTS sequencing. Concordant with WGS results, a single mismatch reduced editing by ~2.7-fold, while two or more mismatches nearly eliminated CBE activity (Fig. 2f and Supplementary Text). Although WGS and HTS results were well correlated, WGS slightly overestimated off-target editing at some loci (one-sample *t*-test and Wilcoxon test *P* = 0.0045; Fig. 2g).

The WGS pipeline revealed that substantial CBE activity (≥5%) occurred at intergenic (364 loci) or intronic (339 loci) regions, while ~28% of edited sites (350 loci across 243 genes) mapped to protein-coding exons. Of these, 145 genes acquired synonymous codon substitutions, while 91 (~37%) acquired missense mutations in ~26% of alleles on average (Fig. 2h), including at least 47 neuronally expressed genes (Supplementary Tables 5–7). Among these, four (*MED12*, *NOLC1*, *PRPF40A* and *RPLP0*) are considered essential according to the Cancer DepMap database^74,75^ (Supplementary Table 8). To assess the impact of nonsynonymous editing, we used AlphaMissense to predict how CAG-CBE-induced amino acid substitutions may affect protein folding and function^76,77^. This analysis revealed that the induced missense mutations mostly result in benign amino acid changes (87%, 53 genes), with one gene (*TSHZ3*, ~10% edited alleles) acquiring mutations likely to affect protein function. Finally, we observed nonsense mutations in a single gene (*PRRC2C*, ~7% edited alleles) that is nonessential and not expressed in neurons^74^ (Supplementary Tables 5–9).

**Extended Data Fig. 2:**
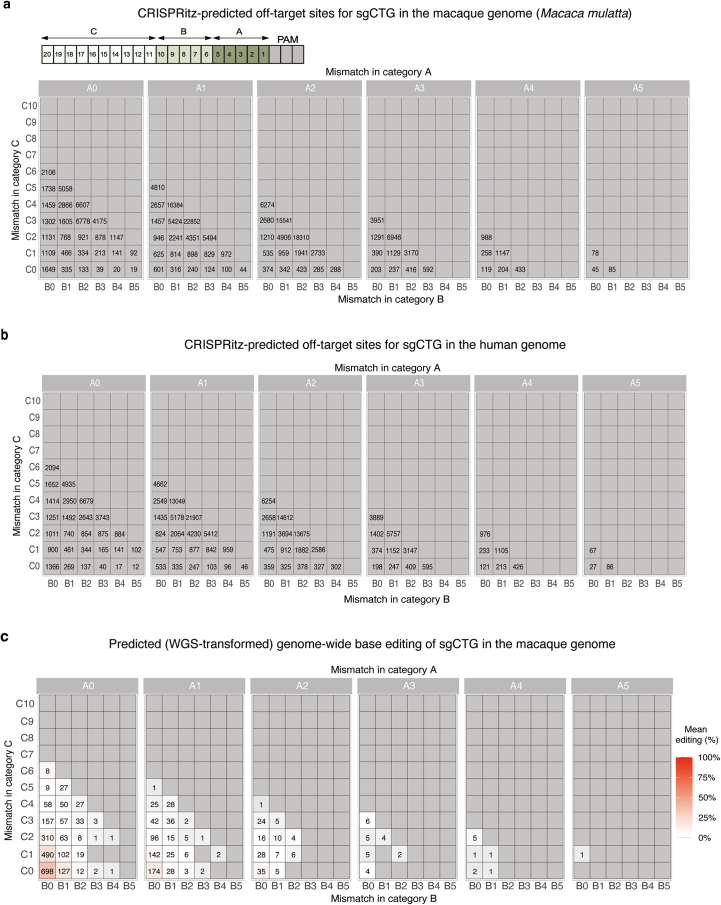
In-silico off-target analysis for CAG repeat-targeting strategy. (**a-b**) CRISPRitz-predicted off-target candidate sites in the (**a**) macaque (*Macaca mulatta)* and (**b**) human genomes organized based on the position of mismatches between the genomic site and sgCTG spacer sequence. Mismatch category A includes the five nucleotides most proximal to the PAM (positions 1-5), category B represents positions 6-10, and category C spans the last ten, PAM-distal nucleotides (positions 11-20) of the protospacer. A0-A5, B0-B5 and C0-C10 indicate the number of mismatches (0-5) between the sgCTG and a target site in categories A, B or C. Each square shows the number of loci in each mismatch subgroup. (**c**) Predicted *in cellulo* base editing off-target activity of our CBE strategy in the macaque genome, based on *in silico* CRISPRitz predictions of off-target edits and a subsequent WGS-transformation using the ratio of off-targets edited in HEK293T cells ( > 0.5% editing by WGS) versus predicted by CRISPRitz in the human genome. Heatmap colors represent predicted *in cellulo* editing per mismatch bin, based on corresponding editing frequencies per bin observed in WGS analysis of edited HEK293T cells.

Collectively, these findings confirm that CAG-CBE introduces C•G>T•A substitutions that are mostly noncoding or synonymous or reproduce natural allelic variation^78,79^. The small fraction of protein-coding off-target loci that undergo editing (6.1% of CIRCLE-seq-nominated sites) merit careful study to understand potential biological consequences of editing at these residues. This work also underscores the limitations of computational predictive models (Supplementary Text and Extended Data Fig. 2a–c), which may overestimate or underreport off-target events, further highlighting the importance of experimental nomination and empirical validation of genome-wide off-target effects.

### Cytosine base editing of HTT alleles reduces CAG length in neurons

The *Htt*.*Q111* mouse model of HD harbors a humanized *HTT* allele with a long pathogenic repeat tract containing ~109–122 CAGs and exhibits age-dependent somatic instability in central nervous system (CNS) tissues including striatum and cortex^80–82^. In contrast, HD knock-in mice that carry (CAGCAACAGCAACAA)_21_, a long poly-Q tract composed of a mixture of CAG and CAA codons, do not exhibit somatic instability^22^, suggesting that interrupting the pathogenic-length CAG tracts may alleviate repeat expansions in vivo.

**Fig. 3:**
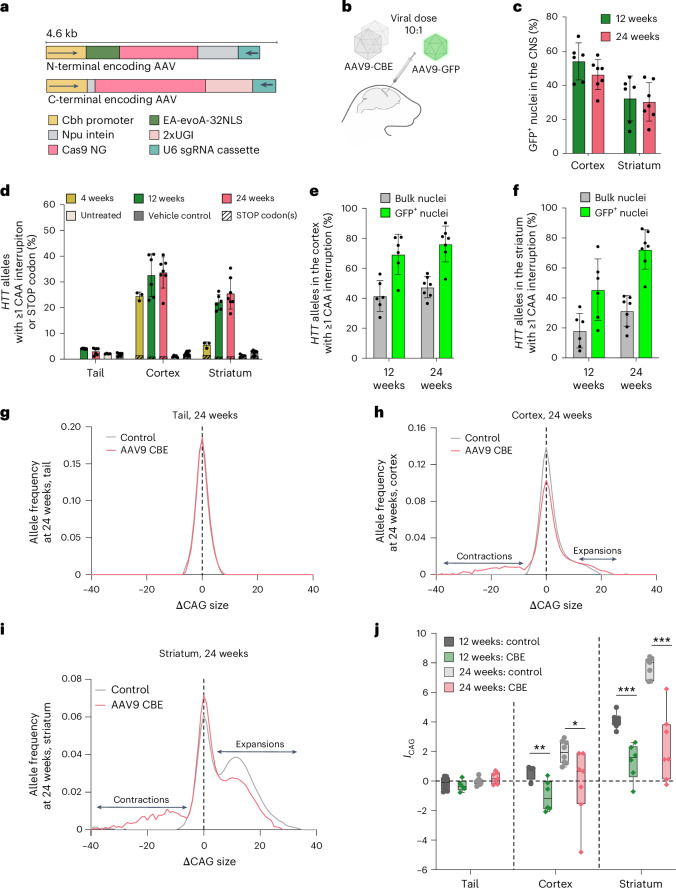
Cytosine base editing of *HTT* CAG repeats in *Htt.Q111* mice. **a**, Dual-AAV vectors encoding split-intein EA-evoA-32NLS-NG and sgCTG cassettes, v5 AAV9-CBE. **b**, Neonatal ICV injections in *Htt.Q111* mice with AAV9-CBE, and AAV9-GFP as a transduction control. **c**, Transduction efficiency in the cortex and striatum of *Htt.Q111* mice treated with AAV9-CBE + AAV9-GFP. Data are mean ± s.d. of independent animals (12-week, *n* = 6; 24-week, *n* = 7). **d**, Base editing in the CNS of *Htt.Q111* mice treated with AAV9-CBE, or controls. Editing was quantified at 4, 12 and 24 weeks postinjection. Data are mean ± s.d. of independent animals (4-week, *n* = 3; 12-week, *n* = 6; 24-week, *n* = 7). **e**,**f**, Base editing in bulk and GFP^+^ flow-sorted nuclei isolated from the cortex (**e**) or striatum (**f**) of *Htt.Q111* mice treated with AAV9-CBE + AAV9-GFP at 12 and 24 weeks postinjection. Data are mean ± s.d. of independent animals (12-week, *n* = 6; 24-week, *n* = 7). **g**–**i**, Distribution of CAG allele sizes in the tail (**g**), cortex (**h**) and striatum (**i**) isolated from 24-week-old *Htt.Q111* mice treated with AAV9-CBE, or controls. The dashed vertical line marks the modal *HTT* CAG allele determined from the tail. Data show mean CAG repeat size distributions from at least four independent animals (tail: untreated *n* = 4, CBE *n* = 11; striatum: untreated *n* = 7, CBE *n* = 7; cortex: untreated *n* = 8, CBE *n* = 7). **j**, *I*_CAG_ calculated for the tail, cortex and striatum isolated from 12- and 24-week-old *Htt*.*Q111* mice treated with AAV9-CBE, or controls. Data are shown as box plots, with each data point representing an independent animal (12 weeks, control group: tail *n* = 8, striatum *n* = 8; cortex *n* = 4; 12 weeks, CBE group: cortex *n* = 6, striatum *n* = 6, tail *n* = 5; 24 weeks, control group: tail *n* = 8, cortex *n* = 8, striatum *n* = 7; 24 weeks, CBE group: all tissues *n* = 7). The horizontal line marks the median, and whiskers denote the minimum and maximum values. *P = 0.0265, **P = 0.0064, ***P = 0.0009, Welch’s one-tailed *t*-test. Illustrations in **a** and **b** were created using BioRender.com. Source data

To assess whether CBE-induced CAA interruption reduces the average length of *HTT* CAG repeats in vivo, we designed a dual adeno-associated virus (AAV) strategy to package CAG-CBE (EA-evoA-32NLS-NG and sgCTG, v5 AAV-CBE; Fig. 3a) for delivery to *Htt*.*Q111* mice^83^. We selected the AAV serotype 9 (AAV9) as it has a well-established tropism for neurons in the CNS and has been shown to almost exclusively target neurons in the cortex^84–86^. We injected *Htt*.*Q111* neonates on postnatal day 0 via intracerebroventricular (ICV) injection with total 3.8 × 10^10^ viral genomes (vg) per mouse of the dual AAV9-CBE vectors, along with 3.8 × 10^9 ^vg of AAV9-Cbh-eGFP-KASH (Klarsicht/ANC-1/Syne-1 homology domain, hereafter, AAV9-GFP)^83^ to serve as a viral transduction control^83,87^ (Fig. 3b). We quantified GFP-positive nuclei from the cortex and striatum of injected mice and observed a mean transduction efficiency of 50 ± 10% and 31 ± 12%, respectively (Fig. 3c), consistent with earlier reports using AAV9 (refs. ^83,88–90^).

**Extended Data Fig. 3:**
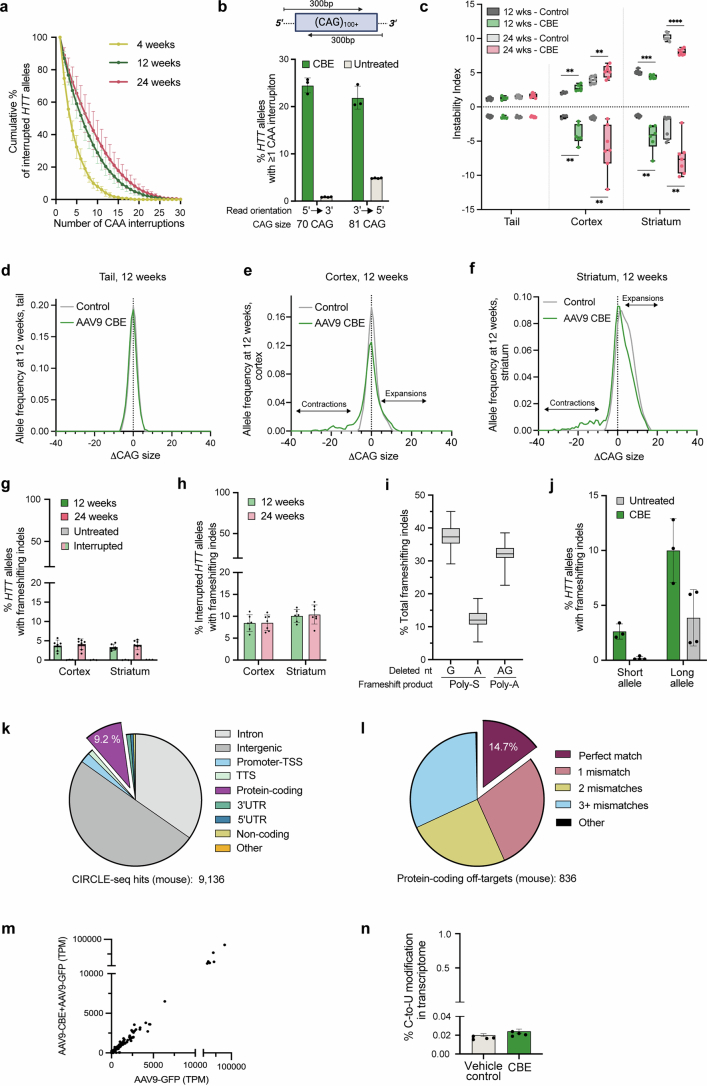
Cytosine base editing of *HTT* CAG repeats in *Htt*.Q111 mice. (**a**) Cumulative mean % interrupted *HTT* alleles with at least a specified number of CAA interruptions in the cortex of AAV9-CBE-treated *Htt*.Q111 mice at 4-, 12- and 24-weeks post-injection. Mean ± SD (4 weeks n = 4, 12 weeks n = 6, 24 weeks n = 7). (**b**) CAG-CBE editing in *Htt*.Q111 mice quantified in 5′- > 3′ and 3′->5′sequencing directions. Mean ± SD (CBE n = 3, untreated n = 4). (**c**) Expansion (I_CAG(e)_, positive) and contraction (I_CAG(c)_, negative) indices in tail, cortex and striatum of 12- and 24-week-old *Htt*.Q111 mice treated with AAV9-CBE, or controls. Box plots of independent animals (Tail: untreated n = 4, CBE n = 5; Striatum: untreated n = 4, CBE n = 6; Cortex: untreated n = 4, CBE n = 6). Median indicated by horizontal line, whiskers show min-max values. **P = 0.007, ***P = 0.0003, ****P < 0.0001, Welch’s one-tailed t-test. (**d-f**) CAG allele sizes in (**d**) tail, (**e**) cortex and (**f**) striatum of 12-week-old *Htt*.Q111 mice treated with AAV9-CBE, or controls. Dotted line indicates modal *HTT* allele. Mean distributions (Tail: untreated n = 8, CBE n = 5; Striatum: untreated n = 8, CBE n = 6; Cortex: untreated n = 4, CBE n = 6). (**g**) Frameshifting indels in AAV9-CBE-treated *Htt.Q111* mice, 12- and 24-weeks post-treatment. Mean ± SD (untreated n = 3, 12 weeks n = 6, 24 weeks n = 7). (**h**) Fraction of interrupted *HTT* alleles with frameshifting indels in AAV9-CBE-tretaed *Htt.Q111* mice at 12 and 24 weeks post-treatment. Mean ± SD (12 weeks n = 6, 24 weeks n = 7). (**i**) Frameshifting indel sequences (G, A and AG) at CAG repeats in cortex and striatum of *Htt*.Q111 mice, 12- and 24-weeks post-injection. Corresponding amino acid sequences (Poly-S – polyserine, Poly-A – polyalanine) noted. Median shown by horizontal line, min-max range by vertical lines (n = 26). (**j**) Frameshifting indels in HD fibroblasts (GM09197) treated with CBE. Mean ± SD (untreated n = 4, CBE n = 3). (**k-l**) CIRCLE-seq off-target hits in the mouse genome classified by (**k**) targeted region and (**l**) mismatch number with sgCTG. (**m**) Differential gene expression analysis in cortex of *Htt.Q111* mice treated with AAV9-CBE + AAV9-GFP or AAV9-GFP vehicle at 12 weeks post-injection (RNA-seq). Each dot represents a transcript. Mean of four animals per group. r = 0.97 across 134,701 transcripts. (**n**) Whole-transcriptome C-to-U RNA off-target analysis in *Htt*.Q111 mice treated with AAV9-CBE + AAV9-GFP or AAV9-GFP vehicle. Mean ± SD of four animals. *P* = 0.0182, Welch’s two tailed t-test. All data points represent independent biological replicates. Source data

At 4 weeks postinjection, we observed CAA interruptions in 24 ± 1.6% and 5.6 ± 1.2% of alleles from cortical and striatal cells, respectively, with no detected nonsense mutations resulting from opposite-strand editing above background levels (Fig. 3d), and with a median of three interruptions and an average of 3.9 per edited allele in the cortex (Extended Data Fig. 3a). As expected given the long-lasting nature of standard AAV expression and the continued availability of long stretches of uninterrupted repeat sequence, base editing levels in these tissues steadily increased up to 12 weeks postinjection, reaching 33 ± 8.3% in the cortex and 22 ± 3.0% in the striatum with a median of six interruptions per edited read (average of 7.6; Fig. 3d and Extended Data Fig. 3a), after which editing largely stabilized and we observed only a modest increase in editing at 24 weeks (34 ± 6.0% edited alleles in the cortex and 26 ± 6.0% in the striatum, with median of eight interruptions and average of 8.8 interruptions per edited read). In transduced cells—enriched by sorting for GFP-positive nuclei^83,87^—we observed that 76 ± 12% and 72 ± 13% of *HTT* alleles from the cortex and striatum, respectively, harbored one or more CAA interruptions by 24 weeks postinjection (Fig. 3e,f). These observed editing frequencies are all underestimates of the true interruption frequency across the entirety of the repeat tract, as they measure editing across only <65% of the repeat region (70 of ≥109 repeats; Supplementary Text and Extended Data Fig. 3b). Collectively, these data confirm that neonatal ICV injection of AAV9-CBE enables efficient synonymous CAA interruption of long pathogenic *HTT* CAG repeats in HD-relevant tissues.

To examine whether CAA interruptions impact somatic CAG repeat expansion in vivo, we performed qualitative and quantitative analyses of CAG repeat length profiles from *Htt.Q111* mice treated with AAV9-CBE compared with controls^80,91^ (Fig. 3g–j and Supplementary Text). At 12 weeks postinjection, we found that AAV9-CBE treatment significantly reduced the average size of CAG repeats (CAG instability index, *I*_CAG_) in the cortex (*I*_CAG_ = −1.6 ± 0.5 repeats, Welch’s one-tailed *t*-test *P* = 0.0064) and striatum (*I*_CAG_ = −2.8 ± 0.5 repeats, Welch’s one-tailed *t*-test *P* = 0.0009; Fig. 3j, Extended Data Fig. 3c–f and Supplementary Text). This effect endured over time, reaching *I*_CAG_ = −2.2 ± 0.9 repeats in the cortex (Welch’s one-tailed *t*-test *P* = 0.0265) and *I*_CAG_ = −5.4 ± 0.9 repeats in the striatum (Welch’s one-tailed *t*-test *P* = 0.0003) at 24 weeks postinjection (Fig. 3g–j).

Collectively, the data reveal that neonatal ICV injection of AAV9-CBE enables substantial transduction of HD-relevant tissues in humanized HD mice, significantly reducing the average size of pathogenic *HTT* CAG repeats by inducing synonymous CAG-to-CAA interruptions with proportionally few byproducts (Supplementary Text, Fig. 3d and Extended Data Fig. 3g–j). This targeted base editing approach in postnatal animals not only prevents repeat expansion but also contracts repeats, offering particular benefits for CAG repeat lengths that exceed the pathogenic threshold, are inherently toxic and cannot become nonpathogenic solely by reducing the rate of somatic expansion^91,92^.

Our findings demonstrate the protective role of CAA interruptions in vivo, and show that CAG-CBE activity primarily results in silent or noncoding sequence changes at off-target loci (Supplementary Text and Extended Data Fig. 3k–n).

### Adenine base editing of GAA repeats at FXN alleles in vitro

Similar to the protective effect of CAG repeat interruptions, GGA and GAG triplet interruptions in the GAA repeat region of *FXN* intron 1 are associated with the absence of FRDA disease phenotypes, later disease onset and milder symptoms compared with patients with similar-sized uninterrupted GAA alleles^21,23,49^. Triplet sequence variation within GAA repeat tracts can also prevent the formation of higher-order DNA structures that underlie repeat expansion and transcriptional repression of *FXN* in vitro^16,21^.

**Fig. 4:**
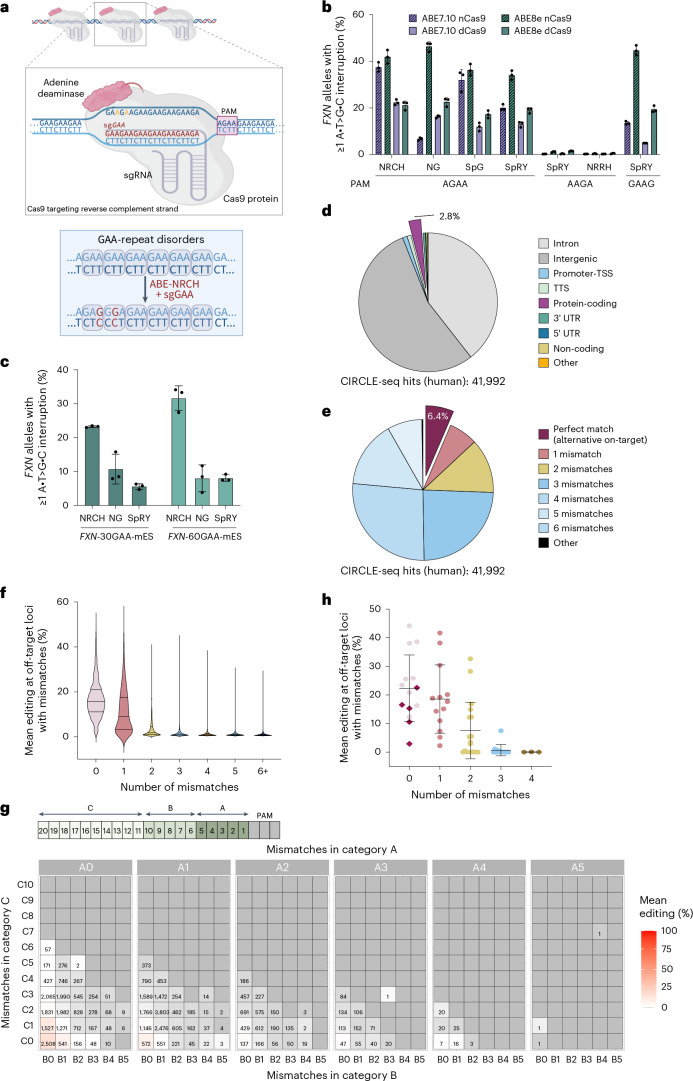
Adenine base editing of *FXN* GAA repeats in vitro*.* **a**, Illustration of adenine base editing at GAA repeats (top) and schematic of the editing strategy (bottom). A smaller cartoon illustrates multiple binding opportunities for the Cas9-sgGAA complex at GAA repeats, with a magnified view showing a single binding event. **b**, Optimization of adenine base editing in HEK293T cells. Sequences below the bar plot indicate the NNNN PAM sequences compatible with sgGAA spacer. Data are mean ± s.d. of biological triplicates. **c**, Comparison of AGAA PAM-targeting ABE8e strategies in *FXN-*mESCs, evaluated across 30 (*FXN*-30GAA-mES) and 50 (*FXN*-60-GAA-mES) GAA repeats. Data are shown as mean ± s.d. of biological triplicates. **d**,**e**, CIRCLE-seq off-target hits in the human genome classified by the identity of the targeted region annotated with HOMER (**d**) and the number of mismatches with the sgGAA (**e**). **f**,**g**, Alternative target and off-target editing at CIRCLE-seq sites in HEK293T cells, confirmed by WGS (>0.5% editing) and classified based on the number (**f**) and the location (**g**) of mismatches relative to the sgGAA spacer. Horizontal lines in **f** mark median and quartiles calculated for all loci in a specific group. Editing for each locus is a mean of triplicates. Mean editing (%) in **g** represents base editing frequency across genomic sites meeting the specified mismatch criteria. Mismatch category A includes the five nucleotides proximal to PAM (positions 1–5), category B represents positions 6–10 and category C spans the last ten, PAM-distal nucleotides (positions 11–20) of the protospacer. A0–A5, B0–B5 and C0–C10 indicate the number of mismatches (0–5) between the sgGAA and a target site in categories A, B and C. Each square shows the number of loci with >0.5% editing in each mismatch subgroup. **h**, Editing frequencies at CIRCLE-seq sites with 0–4 mismatches between the sgGAA and a target site in HEK293T cells, measured by HTS. Each dot shows mean editing at a unique locus; diamonds indicate protein-coding sites. Data are mean ± s.d. of all loci in each category. Data in **f**–**h** represent biological triplicates. Illustration in **a** was created using BioRender.com. TSS, transcription start site; UTR, untranslated region. Source data

We hypothesized that inducing A•T>G•C interruptions at GAA repeats using ABEs could mimic the natural genetic variation of *FXN* alleles that is observed in the general population and in individuals with pathogenic-length GAA repeats that are disease-free, or have later onset compared with patients with FRDA with similar repeat length^21,23,49^ (Table 1 and Supplementary Text). We speculated that A•T>G•C interruptions at GAA repeats could reduce the length of pathogenic repeat expansions that are causal to FRDA (Figs. 1a and 4a). To induce A•T>G•C interruptions throughout GAA repeat tracts, we designed three sgRNAs to target pure GAA triplets using ‘AGAA’, ‘AAGA’ or ‘GAAG’ as a PAM. We paired these sgRNAs with ten different ABEs that used the laboratory-evolved deoxyadenine deaminases from either ABE7.10 or ABE8e (refs. ^56,93^) fused to PAM-compatible Cas-proteins, including Cas9-NG, Cas9-NRCH, Cas9-SpG, Cas9-SpRY and Cas9-NRRH^54,94–97^ (Fig. 4b). We assessed ABE editing at *FXN* alleles of HEK293T cells, which on average harbor nine GAA repeats, by HTS and powTNRka analysis to quantify the fraction of *FXN* alleles with at least one A•T>G•C interruption. We observed the highest fraction of edited *FXN* alleles with strategies using the AGAA PAM (42 ± 2.9% by ABE8e-Cas9-NRCH and 46 ± 2.0% by ABE8e-Cas9-NG) and GAAG PAM (45 ± 2.1% by ABE8e-Cas9-SpRY; Fig. 4b).

**Table 1:** General characteristics of GAA expansions of all samples with biallelic GAA expansions in the UK Biobank

ID	EHv4: genotype	EHv4: genotype CI	Diagnosed with hereditary ataxia	Manual review: genotype quality	Manual review: summary of interruptions	Manual review: observed interruptions
1	135/135	68–149/90–189		Low	Pure	GAAA
2	129/129	58–152/85–206	Yes	Low	Pure	GAAA
3	127/127	73–149/95–184		Low	Pure	
4	125/125	73–158/97–197		Medium	Interrupted	GAG
5	114/114	68–138/88–171	Yes	Medium	Pure	GAAA
6	112/112	64–139/87–176		High	Interrupted	GAG
7	109/109	53–143/81–197	Yes	Medium	Pure	
8	109/109	62–135/85–172		High	Interrupted	GAG, GAAA
9	105/105	50–134/71–201		Medium	Interrupted	GAG
10	103/103	29–145/74–208		Medium	Interrupted	GAG
11	102/102	53–118/76–161		Low	Interrupted	GAG, GAAA
12	100/100	53–138/78–193		Low	Interrupted	GAG
13	100/100	49–146/78–207		Medium	Interrupted	GAG, GAAA
14	98/98	43–142/69–203		Medium	Pure	
15	98/98	48–120/73–173		High	Interrupted	GAG, GAAA
16	96/96	51–121/75–165		High	Interrupted	GAG, GAAA
17	95/95	50–115/73–162	Yes	High	Pure	GAAA
18	90/90	47–127/70–195		Medium	Interrupted	GAG, GAAA
19	90/90	44–97/66–149		Medium	Interrupted	GAG
20	89/89	49–130/73–184		Medium	Interrupted	GAG
21	89/89	50–132/73–183		Low	Pure	
22	85/85	53–121/75–163		Low	Interrupted	GAG
23	85/85	44–114/66–165		Medium	Pure	
24	84/84	48–106/67–153		Medium	Interrupted	GAG, GAAA
25	83/83	38–121/63–178		Medium	Pure	
26	82/82	49–94/64–129		High	Interrupted	GAG, GAAA
27	82/82	40–124/64–210		Medium	Interrupted	GAG, GAAA
28	82/82	48–102/65–151		Medium	Interrupted	GAG, GAAA
29	79/79	39–98/61–150		Medium	Interrupted	GAG
30	79/79	49–103/67–165		Medium	Interrupted	GAG, GAAA
31	66/66	45–84/59–114		High	Interrupted	GAG, GAAA

Supporting data are included in Supplementary Table 1 and Supplementary Note 1. ID, patient identifier; EHv4: genotype, the diploid FXN genotype reported by ExpansionHunter v.4 for this sample, in the format: ‘number of repeats in allele 1/number of repeats in allele 2’; EHv4: genotype CI, ExpansionHunter’s reported confidence intervals for the genotype, having the format: ‘allele1_lower_bound–allele1_upper_bound/allele2_lower_bound–allele2_upper_bound’ (Methods).

GAA repeats are one of the most abundant triplet-repeat sequences in the human genome. They are found in G/A islands, mostly in Alu elements, with nearly 600 genomic integration sites harboring at least eight GAA repeats that support sgRNA binding and base editing by our strategy—several fold more than the ~100 sites in the human genome that harbor at least eight CAG repeats^68,98^. The most efficient base editors use a Cas9 nickase to bias the permanent incorporation of the edited base pair at the target locus by DNA repair^52,56^. While Cas9 nickase and canonical base editors are typically associated with minimal indels^54,64^, we reasoned that a high number of nicking events from the abundance of GAA repeats in the genome may adversely affect genomic stability. Since nicking is not essential to base editing and current ABEs are highly efficient, we assessed whether ABEs that use nuclease-dead Cas9 (D10A + H840A, dCas9) enable efficient A•T>G•C interruption of GAA repeats. Concordant with their nickase-Cas9 (D10A) ABE8e counterparts, we observed the highest fractions of edited *FXN* alleles from non-nicking ABEs using the AGAA PAM by dCas9-NRCH (21 ± 1.9%) and dCas9-NG (23 ± 1.8%), and the GAAG PAM by dCas9-SpRY (19 ± 1.4%; Fig. 4b), with overall fractions of edited *FXN* alleles being approximately half those of the nicking ABE variants.

**Extended Data Fig. 4:**
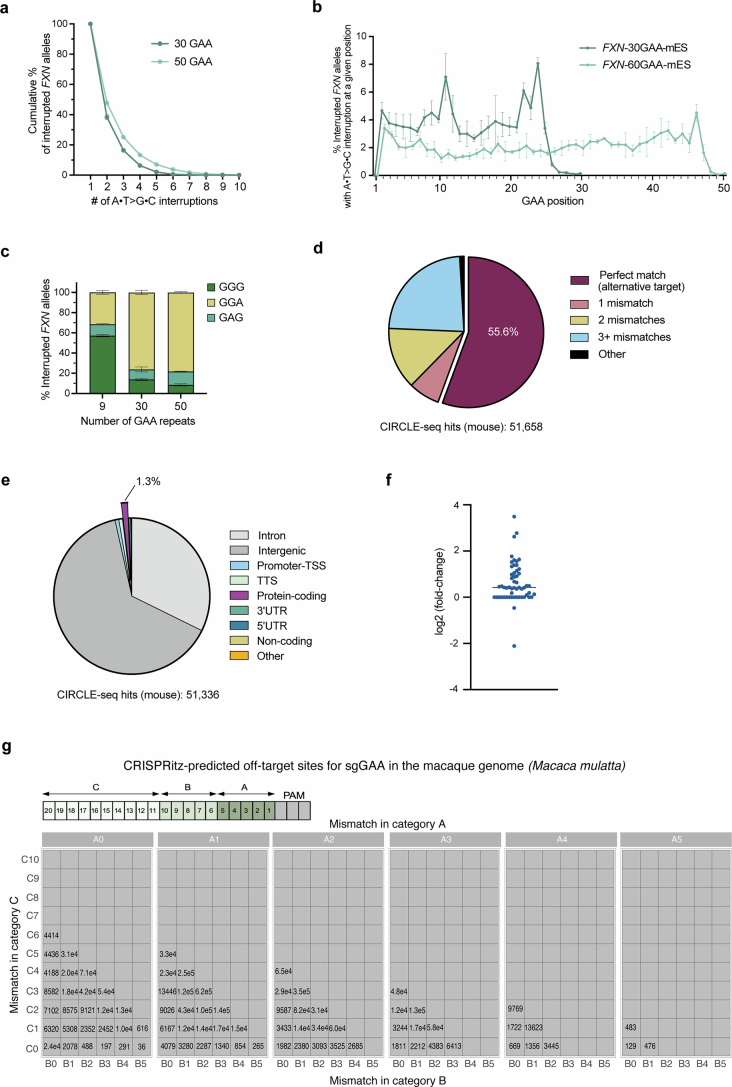
Adenine base editing of *FXN* GAA repeats in vitro. (**a**) Cumulative % of interrupted *FXN* alleles with at least a specified number of A•T > G•C interruptions induced by GAA-ABE in *FXN-mESCs* and measured across 30 (*FXN*-30GAA-mES) and 50 (*FXN*-60GAA-mES) GAA repeats. Data are mean ± SD of biological triplicates. (**b**) Distribution of A•T > G•C interruptions throughout GAA repeats at interrupted *FXN* alleles in *FXN-mES* cells shown as % interrupted alleles with an A•T > G•C interruption at a given position in the GAA repeat tract. Data are mean ± SD of biological triplicates. (**c**) Composition of the A•T > G•C interruptions (GGG, GGA and GAG sequences) introduced at *FXN* alleles in HEK293T cells (9 GAAs) and *FXN-*mESCs (30 and 50 GAAs). Data are mean ± SD of biological triplicates. (**d-e**) CIRCLE-seq off-target hits in the mouse genome classified by (**d**) the number of mismatches with the sgGAA and (**e**) the identity of the targeted region annotated with HOMER. (**f**) Comparison of base editing frequencies quantified by WGS and amplicon sequencing (HTS) at selected sites. Each dot represents the average ratio of editing frequencies quantified by WGS and HTS at a single locus, obtained from biological triplicates. Data are plotted as log2 fold-change, with the median indicated by the horizontal line (n = 55). *P* < 0.0001, One sample t and Wilcoxon test. (**g**) CRISPRitz-predicted off-target sites in the macaque (*Macaca mulatta)* genome, categorized by mismatch position between the sgGAA and genomic site. Category A represents the five nucleotides proximal to PAM (positions 1-5), category B covers the next five nucleotides (positions 6-10), and category C includes the last ten, PAM-distal nucleotides (positions 11-20) of the protospacer. A0-A5, B0-B5 and C0-C10 denote the number of mismatches (0-5) between the sgGAA and a target site in each category. Each square shows the number of loci in each mismatch subgroup. Source data

Next, we evaluated our ABE-dCas9 editing strategies in mouse embryonic stem cell (mESC) lines harboring the human *FXN* intron 1 locus with long GAA repeats^99,100^ (Supplementary Text and Extended Data Fig. 4a–c). We found that ABE8e fused to dCas9-NRCH greatly outperformed other ABEs by up to 4.2-fold (Fig. 4c) and that the editing efficiency of GAA repeats generally increased with the length of the repeat tract (23 ± 0.2% in *FXN*-30GAA-mES compared with 32 ± 3.7% in *FXN*-60GAA-mES; Fig. 4c). Overall, these data demonstrate that the ABE8e-dNRCH base editor in combination with the GAA repeat-targeting sgRNA (sgGAA; a strategy hereafter designated GAA-ABE) enables efficient interruption of *FXN* GAA repeat alleles at both endogenous *FXN* loci and longer-length GAA repeats.

### Genome-wide off-target editing analysis of GAA repeat base editing

The GAA-ABE strategy enables site-specific interruptions at pathogenic GAA expansion loci that underlie neurodegenerative diseases, including FRDA and late-onset cerebellar ataxias^2,101–103^. However, there are many coding and noncoding regions in the genome with ≥8 GAA repeats that could support editing by GAA-ABE, including non-disease-associated regions^68^.

To investigate Cas-dependent genome-wide activity of GAA-ABE, we performed CIRCLE-seq on human (HEK293T) and mouse (NIH3T3) gDNA^69^ using Cas9-NRCH ribonucleoprotein complexes with sgGAA^69,104^ (Supplementary Text). We identified 41,992 putative GAA-ABE off-target sites in the human genome, including 2,703 perfect-match loci (6.4% of CIRCLE-seq-nominated loci), of which only six were in protein-coding regions (Fig. 4d,e and Supplementary Tables 15 and 16). Most CIRCLE-seq-nominated sites (94%) contained mismatches that reduce binding and editing efficiency, and the overwhelming majority (>97%) were in noncoding genomic regions.

To characterize genome-wide GAA-ABE editing, we performed 160× WGS in HEK293T cells. We detected A•T>G•C interruptions at ~50% (20,560 sites) of CIRCLE-seq-nominated loci, with 5,085 sites (~12%) showing ≥5% editing (Supplementary Table 19). Notably, 3,857 edited sites were within Alu elements, which often contain GAA, GGA, GAG and GGG triplets^98^.

Genome-wide GAA-ABE editing decreased with an increasing number of mismatches with the sgGAA^70–72^ (Fig. 4f–h and Supplementary Text). We mainly observed GAA-ABE activity at perfect-match loci (2,497 loci), including five protein-coding sites, with an average editing of 21 ± 9.4% across these sites. Additionally, we detected low-level editing (3.6% on average) at 475 protein-coding off-target loci (Supplementary Tables 16 and 19). Single-amplicon sequencing confirmed that editing efficiency decreased as the number of mismatches increased (Fig. 4h, Extended Data Fig. 4f and Supplementary Text).

Substantial GAA-ABE activity (≥5% editing) occurred mainly in intergenic (3,201 hits, 62%) and intronic (1,755 hits, 34%) regions, with only 83 protein-coding sites (~1.6%) affected. Among 57 genes with nonsynonymous substitutions, 29 are neuronally expressed^105^ and three (*DKC1*, *NOC3L* and *UPF2*) are considered essential, including one expressed in neurons (*DKC1*)^74,75,105^ (Supplementary Tables 19–23). AlphaMissense^76^ predicted that most missense mutations (36 genes) were benign, including those in all three essential genes^74,75^. However, four genes (*B4GALT6*, *BRD9*, *EIF2S2* and *RIMS1*) acquired amino acid changes predicted to impact protein folding or function (Supplementary Table 24). No nonsense mutations were detected at any locus (Supplementary Tables 20 and 21). Collectively, we observed substantial editing at ~12% of CIRCLE-seq-nominated loci.

**Extended Data Fig. 5:**
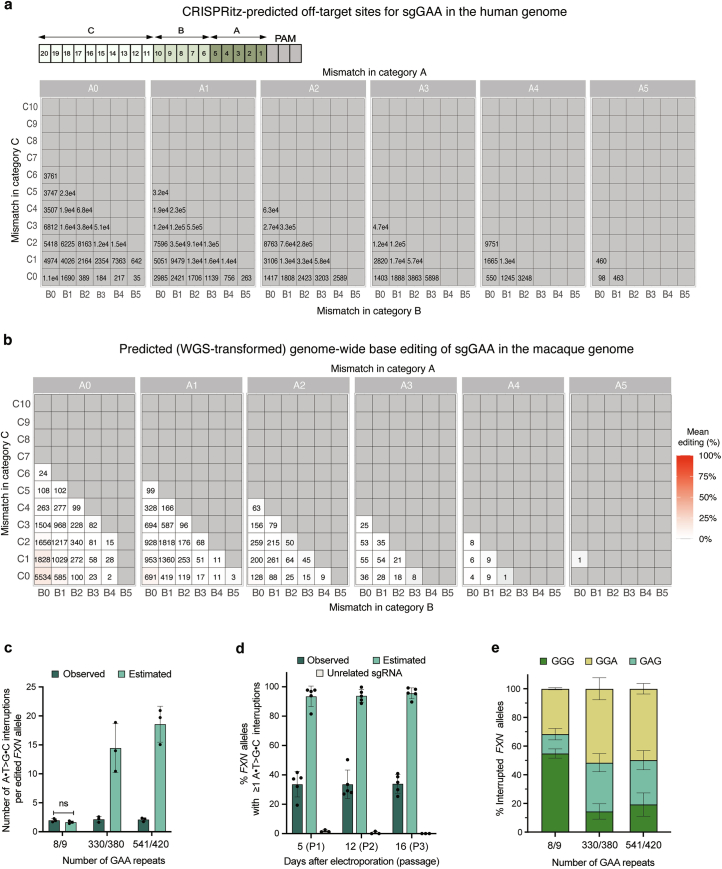
Adenine base editing of *FXN* GAA repeats. (**a**) CRISPRitz-predicted off-target sites in the human genome, categorized by mismatch position between the sgGAA and genomic site. Category A represents the five nucleotides proximal to PAM (positions 1-5), category B covers the next five nucleotides (positions 6-10), and category C spans the last ten, PAM-distal nucleotides (positions 11-20) of the protospacer. A0-A5, B0-B5 and C0-C10 denote the number of mismatches (0-5) between the sgGAA and a target site in each category. Each square shows the number of loci in each mismatch subgroup. (**b**) Predicted *in cellulo* base editing off-target activity of our ABE strategy in the macaque genome, based on *in silico* CRISPRitz predictions of off-target edits and a subsequent WGS-transformation using the ratio of off-targets edited in HEK293T ( > 0.5% editing by WGS) versus predicted by CRISPRitz in the human genome. Heatmap colors represent predicted *in cellulo* editing efficiency per mismatch bin, based on the corresponding editing frequencies per bin observed in WGS analysis of edited HEK293T cells. (**c**) Average number of A•T > G•C interruptions in edited *FXN* alleles isolated from control or FRDA patient-derived fibroblasts, directly observed or estimated. Data are mean ± SD of biological triplicates. ns – not significant, Welch’s two-tailed t-test. (**d**) Observed and estimated *FXN* GAA repeat editing in FRDA patient-derived fibroblasts (GM04078, 541/420 GAAs) 5-16 days and 1-3 cell passages (P) after electroporation, normalized to untreated controls. Data are mean ± SD of biological replicates (unrelated sgRNA n = 3, ABE-treated n = 5). (**e**) Composition of A•T > G•C interruptions (GGG, GGA and GAG sequences) introduced at *FXN* alleles in control (8/9 GAA repeats) or FRDA patient-derived fibroblasts (330/380 or 541/420 GAAs). Data are mean ± SD of biological triplicates. Source data

Together, these results demonstrate that GAA-ABE primarily targets GAA repeats at noncoding regions of the human genome (>98%)^23,49^. Over 87% of candidate off-target sites harbor ≥2 mismatches with the sgGAA, which greatly reduces binding and editing activity^70–72^. As with CAG-CBE, our results suggest that computational predictions may misrepresent off-target editing (Supplementary Text and Extended Data Figs. 4g and 5a,b), emphasizing the need for empirical validation.

### Adenine base editing of GAA repeats in FRDA patient cells

**Fig. 5:**
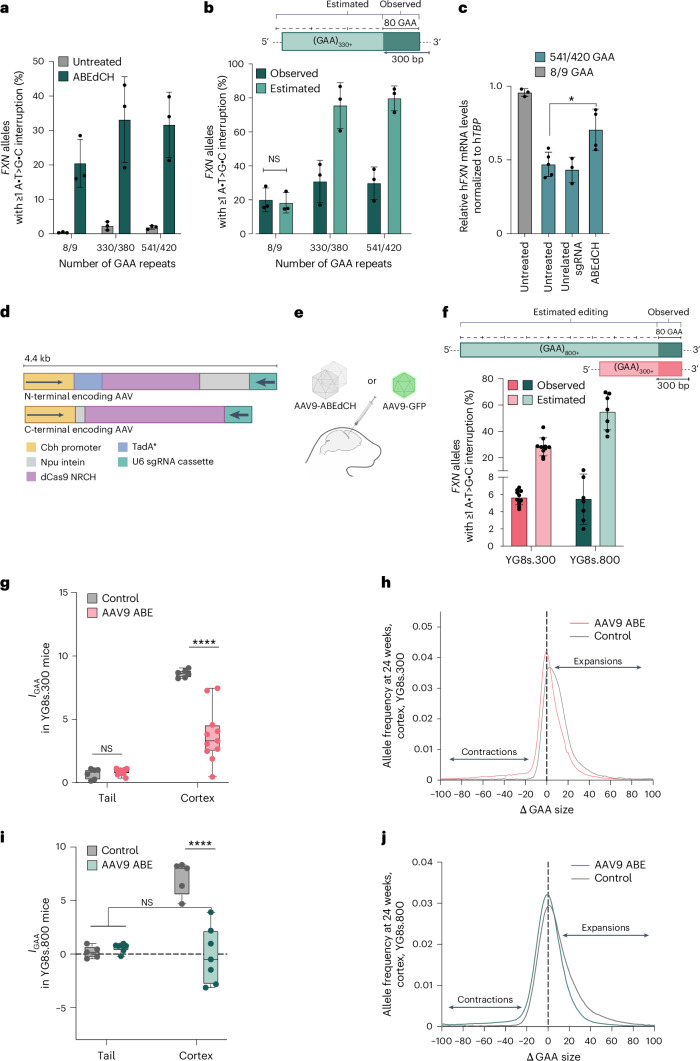
Adenine base editing of *FXN* GAA repeats in patient cells and in YG8s mice. **a**, Base editing of *FXN* GAA repeats in control and FRDA patient-derived fibroblasts. Numbers below the bar plot indicate the size of GAA repeats in each cell line. Data are mean ± s.d. of biological triplicates. **b**, Observed and estimated *FXN* GAA repeat editing in control and FRDA patient-derived fibroblasts, normalized to untreated controls. Data are mean ± s.d. of biological triplicates. NS, not significant, Welch’s two-tailed *t*-test. **c**, *FXN* mRNA expression in human fibroblasts treated with ABEdCH or in controls, 12 d after electroporation, normalized to *TBP* levels. Data are mean ± s.d. of biological triplicates. *P = 0.017, Welch’s one-tailed *t*-test. **d**, Dual-AAV vectors encoding split-intein ABE8e-dNRCH and sgGAA cassettes, v6 AAV9-ABE. **e**, Neonatal ICV injections in YG8s mice with AAV9-ABEdCH. **f**, *FXN* GAA repeat editing in the cortex of YG8s.300 and YG8s.800 mice treated with AAV9-ABEdCH at 24 weeks postinjection, as observed in HTS or estimated, normalized to uninjected controls. Data are mean ± s.d. of independent animals (YG8s.300 *n* = 10, YG8s.800 *n* = 7). **g**,**h**, *I*_GAA_ (**g**) and mean distribution of GAA allele sizes ($\Delta$GAA size) (**h**) in the cortex isolated from 24-week-old YG8s.300 mice treated with AAV9-ABEdCH, or controls. **i**,**j**, *I*_GAA_ (**i**) and mean distribution of GAA allele sizes (ΔGAA size) (**j**) in the cortex isolated from 24-week-old YG8s.800 mice treated with AAV9-ABEdCH, or controls. Data in **g** and **i** are shown as box plots, with each data point representing an independent animal (YG8s.300: untreated *n* = 6, ABE *n* = 10; YG8s.800: untreated *n* = 6, ABE *n* = 7). The horizontal line marks the median, and whiskers represent the minimum and maximum values. ****P < 0.0001, Welch’s one-tailed *t*-test. **h**,**j**, Mean GAA repeat size distributions from at least four independent animals (YG8s.300: untreated *n* = 6, ABE *n* = 10; YG8s.800: untreated *n* = 4, ABE *n* = 8). The dashed line marks the modal *FXN* GAA allele determined from the tail. Illustrations in **d** and **e** were created using BioRender.com. Source data

Small repeat interruptions, such as the A•T>G•C base changes introduced by our GAA-ABE strategy, are commonly found in *FXN* alleles in the general population and are associated with greater stability of GAA repeat alleles^21,106^, higher expression of *FXN* genes, milder disease or later disease onset in patients with FRDA compared with those with uninterrupted repeats of a similar length^16,21,23,49,106,107^. To assess GAA-ABE activity on pathogenic expanded GAA repeats, we quantified repeat interruptions in two primary patient fibroblast cell lines that each carry two long pathogenic *FXN* alleles with ~330/380 (GM03816) or ~541/420 (GM04078) GAA repeats. We treated these cells by mRNA electroporation of the ABE8e-dNRCH editor and synthetic sgGAA^47,57^, and after 5 d observed 20 ± 7.0% average repeat interruption in control fibroblasts (8 or 9 GAA repeats), and 33 ± 12% and 32 ± 9.5% interruption in GM03816 and GM04078 alleles, respectively (Fig. 5a,b, Supplementary Text and Extended Data Fig. 5c). Detected interruption frequencies remained stable over time (Extended Data Fig. 5d), and we observed a positive correlation between editing efficiency and repeat length, concordant with GAA-ABE editing in HEK293T and mES-*FXN* reporter cell lines (Fig. 4b,c), and consistent with the increased opportunity for base editor binding at longer GAA repeats.

To evaluate the impact of ABEdCH-mediated GAA interruptions on *FXN* expression in alleles with pathogenic-length GAA repeats, we quantified *FXN* transcript levels in FRDA patient fibroblasts relative to untreated cells and wild-type controls. We found that ABE treatment increased *FXN* mRNA expression ~1.5-fold in treated FRDA fibroblasts, from ~49% to ~74% of wild-type levels, as measured by digital droplet PCR (Fig. 5c). These results suggest that base editing of pathogenic GAA repeats may alleviate *FXN* transcriptional repression to increase transcript levels, consistent with previous reports that correlate *FXN* repeat purity with transcriptional repression^16,21,106,107^.

Taken together, these data demonstrate that GAA-ABE enables efficient editing of pathogenic GAA repeat tracts in *FXN* alleles of patients with FRDA, significantly increasing *FXN* transcript levels and suggesting that base editing of GAA repeats can partially rescue molecular hallmarks of FRDA in human cells^16,21^. The most prevalent (>80%) interruptions induced by GAA-ABE at *FXN* were GAG and GGA (Extended Data Fig. 5e), which represent the most common *FXN* triplet interruptions in the general population. Thus, GAA-ABE produces *FXN* genotypes that resemble natural genetic variants associated with improved phenotypic outcomes in individuals with expanded GAA repeats^23,49^, and alleviate molecular markers of disease in FRDA cell lines^16,21^.

### Adenine base editing of FXN alleles reduces GAA length in neurons

FRDA patient-derived fibroblasts used in this study do not exhibit measurable GAA repeat instability^108,109^. In contrast, the YG8s.300 and YG8s.800 mouse models of FRDA harbor a human *FXN* YAC transgene with ~300 and 800 GAA units, respectively, that undergo progressive instability starting at ~18 weeks of age that is biased toward expansion of repeats in somatic tissues, including the cortex, striatum and the liver, but not the tail^110–112^.

**Extended Data Fig. 6:**
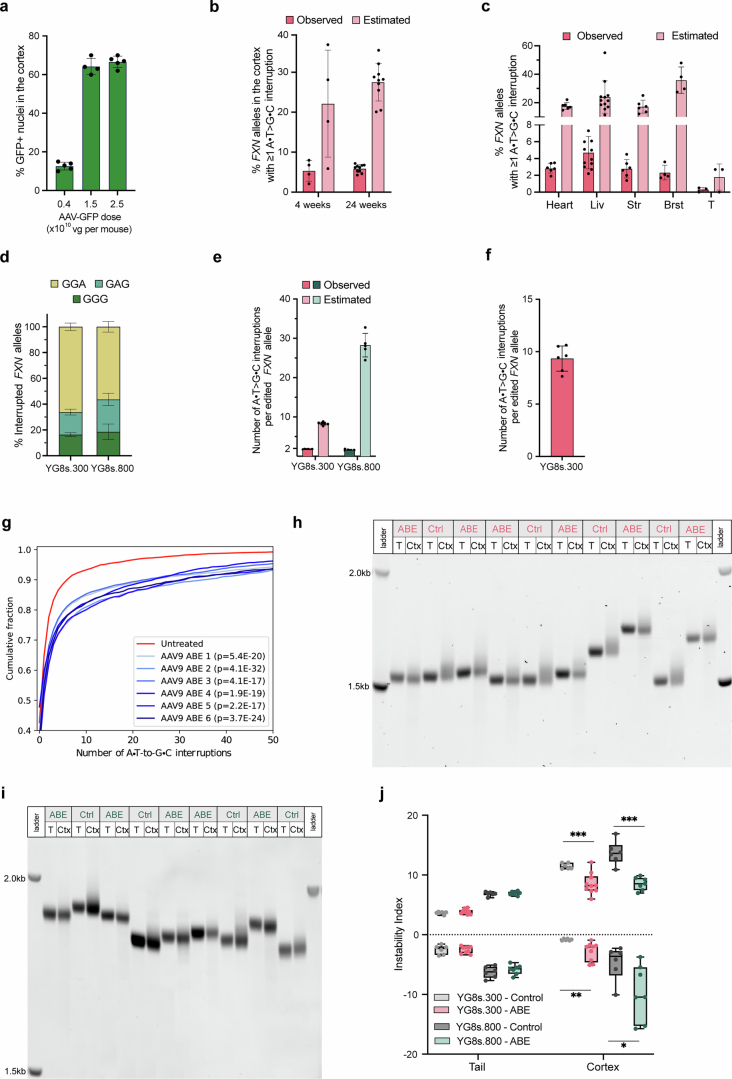
Adenine base editing of *FXN* GAA repeats in YG8s mice. (**a**) Transduction efficiency in the cortex of YG8s mice treated with AAV9-GFP at 0.4-2.5×10^10^ vg/mouse. Mean ± SD (0.4 and 2.5×10^10^vg n = 5,1.5 ×10^10^vg n = 4). (**b**) *FXN* GAA repeat editing in heart, liver (Liv), striatum (Str), brainstem (Brst), and tail (T) of YG8s.300 mice treated with AAV9-ABEdCH at 24 weeks post-injection, observed by HTS or estimated, normalized to controls. Mean ± SD (heart and striatum n = 6, liver n = 12, brainstem n = 4, tail n = 3). (**c**) *FXN* GAA repeat editing in the cortex of YG8s GAA.300 mice treated with AAV9-ABEdCH at 4 and 24 weeks post-injection, observed by HTS or estimated, normalized to uninjected controls. Mean ± SD (4 weeks n = 4, 24 weeks n = 10). (**d**) Composition of A•T > G•C interruptions (GGG, GGA and GAG sequences) introduced at *FXN* alleles in 24-week-old YG8s mice treated with AAV9-ABEdCH. Mean ± SD (n = 6). (**e**) Average number of A•T > G•C interruptions in edited *FXN* alleles in 24-week-old YG8s mice as observed by HTS or estimated. Mean ± SD (YG8s.300 n = 6, YG8s.800 n = 5). (**f**) Average number of A•T > G•C interruptions in edited *FXN* alleles in 24-week-old YG8s.300 mice measured by nanopore sequencing. Mean ± SD (n = 6). (**g**) Cumulative probabilities of a given number of A•T > G•C interruptions in *FXN* alleles isolated from the cortex of YG8s.300 mice treated with AAV9-ABEdCH (n = 6) or uninjected controls (reference probability, mean of n = 2), determined by nanopore sequencing (Kolmogorov-Smirnov, p < 1.58^−14^. (**h-i**) Representative agarose gels used for quantification of GAA instability index in (**h**) YG8s.300 and (**i**) YG8s.800 mice treated with AAV9-ABEdCH (ABE), or controls (Ctrl); T- tail, Ctx – cortex. Each animal tissue was analyzed on the gel at least twice with similar results. (**j**) Expansion (I_GAA(e)_, positive) and contraction (I_GAA(c)_, negative) indices in tail and cortex of 24-week-old YG8s mice treated with AAV9-ABEdCH, or controls. Box plots of biological replicates (YG8s.300 n = 11, YG8s.800 n = 7). Median indicated with horizontal lines, whiskers show min-max values. *P = 0.0203, **P = 0.0022, ***P < 0.0003, Welch’s one-tailed t-test. All data points represent independent biological replicates. Source data

To assess how A•T>G•C interruption of long pathogenic GAA repeats affects repeat expansion at *FXN* alleles in vivo^83–87^, we injected the dual AAV9-ABEdCH vectors at a total dose of 3.8 × 10^10^ vg per mouse^83,88–90^ (Fig. 5d,e and Supplementary Text). At 24 weeks postinjection, the estimated editing in cortical alleles reached 28 ± 6.8% in YG8s.300 mice (of which 5.6% ± 0.8% was directly observed) and 55 ± 14% in YG8s.800 mice (of which 5.5% ± 2.9% was directly observed) (Fig. 5f, Supplementary Text and Extended Data Fig. 6c). Editing predominantly resulted in GAA-to-GGA changes (66 ± 2.9% of interruptions in YG8s.300 mice and 56 ± 4.0% of interruptions in YG8s.800 mice; Extended Data Fig. 6d), concordant with the results of GAA-ABE editing of pathogenic-length GAA repeats in vitro (Extended Data Fig. 5e). We estimated that edited *FXN* alleles acquire 8.3 A•T>G•C interruptions per 300 GAA repeats on average (1.8 A•T>G•C observed interruptions; Extended Data Fig. 6e). These estimates are in general agreement with long-read nanopore sequencing of edited alleles, which revealed up to 9.3 ± 1.2 A•T>G•C interruptions per ~300 GAAs in *FXN* alleles of treated YG8s mice (Extended Data Fig. 6e–g and Supplementary Text).

Next, we assessed whether A•T>G•C interruptions impact somatic GAA expansion in vivo. We quantified GAA instability index (*I*_GAA_) in AAV9-ABEdCH-treated and control YG8s mice^80,113^ (Methods). At 24 weeks postinjection, AAV9-ABEdCH treatment significantly reduced the average size of GAA repeats in the cortex compared with control animals (*I*_GAA_; Fig. 5g–j and Extended Data Fig. 6h,i). In YG8s.300 mice, the average GAA repeat size decreased by *I*_GAA_ = −4.9 ± 0.6 repeats (Fig. 5g,h), while in YG8s.800 mice, we observed even greater reduction of *I*_GAA_ = −7.2 ± 1.2 repeats (Fig. 5i,j; Welch’s one-tailed *t*-test *P* < 0.0001 and *P* < 0.0001, respectively). Notably, cortical GAA size variation in treated YG8s.800 mice did not differ from tail tissue, which does not undergo repeat expansion (not significant, Welch’s one-tailed *t*-test; Fig. 5i), suggesting that AAV9-ABEdCH effectively halts spontaneous cortical expansions (Fig. 5i and Extended Data Fig. 6j).

AAV9-ABEdCH reduced somatic repeat expansions (expansion index, *I*_GAA(e)_) in YG8s.300 mice (*I*_GAA(e)_ = −2.9 ± 0.6 repeats, Welch’s one-tailed *t*-test *P* = 0.0002) and in YG8s.800 mice (*I*_GAA(e)_ = −5.2 ± 0.9 repeats, Welch’s one-tailed *t*-test *P* = 0.0003; Extended Data Fig. 6j) while also promoting repeat contractions (contraction index, *I*_GAA(c)_). At 24 weeks postinjection, we observed a reduction in GAA repeat length in cortical *FXN* alleles of YG8s.300 mice (*I*_GAA(c)_ = −2.0 ± 0.5 repeats, Welch’s one-tailed *t*-test *P* = 0.0022) and, to an even greater extent, in YG8s.800 mice (*I*_GAA(c)_ = −5.0 ± 2.1 repeats, Welch’s one-tailed *t*-test *P* = 0.0203), compared with controls (Extended Data Fig. 6j), suggesting that longer *FXN* alleles are more prone to contractions in YG8s mice.

Collectively, these data establish that neonatal ICV injection of AAV9-ABEdCH enables substantial transduction of FRDA-relevant tissues that undergo somatic repeat expansion. The direct installation of A•T>G•C interruptions using this delivery method at pathogenic-length *FXN* alleles significantly reduces GAA repeat size by limiting repeat expansions and inducing repeat contractions, highlighting that base editing can both stabilize and contract pathogenic *FXN* GAA repeats in postnatal animals.

## Discussion

TNR diseases affect ~1 in 3,000 individuals worldwide^1^. Recently approved Skyclarys (omaveloxolone) is the first treatment for FRDA that delays disease progression in some patients^114,115^, and symptom management plans provide relief to patients living with poly-Q disorders including HD. To date, however, there are no therapeutic interventions that reverse or stop the motor and neurological decline of any TNR disorder.

The *Htt*.*Q111* and YG8s mouse models used in our study do not exhibit the motor and behavioral phenotypes observed in patients with HD and FRDA. Mouse models of FRDA that harbor human pathogenic *FXN* alleles on a mouse *Fxn* knockout background (YG8-800 (ref. ^116^)) or HD mouse models with longer CAG repeats (Q140 (ref. ^117^)) may better recapitulate both the molecular and physiological human disease manifestations. Future studies using such models may help validate whether reduced expansions of pathogenic-length *TNR* alleles following base editor treatment of expanded repeats improves motor and behavioral function to further verify the protective role of repeat interruptions in TNR disorders.

Here, we packaged our repeat-targeting base editing strategies into AAV9 vectors and delivered to murine neonates via ICV injection. We observed that base editing of pathogenic repeats reduced repeat expansions in the CNS of *Htt*.*Q111* and YG8s mouse models of HD and FRDA. However, GAA repeat expansion and frataxin deficiency in patients with FRDA affect non-neuronal tissues as well, including glial cells^118,119^, which may exacerbate neural degeneration in FRDA. Moreover, patients with FRDA often develop a cardiomyopathy associated with heart failure and death^120^. While AAV9 vectors have a well-established neuronal tropism and have been demonstrated to primarily target neurons in the CNS^84–86^, alternative AAV serotypes and routes of administration may enable greater editing and treatment of additional FRDA disease-relevant tissues in older animals^83,121^. Such approaches would facilitate the investigation of later-stage rescue of repeat instability in vivo, including at alleles that already exhibit measurable somatic instability.

AAV delivery results in potentially long-term base editor expression, which promotes the continued accumulation of repeat interruptions in vivo. Prolonged base editor expression may also result in an increase in off-target editing events; however, in previous studies we have not observed an increased accumulation of genomic off-targets from constitutive base editor expression in vivo over time^89^. Unintended genomic changes may affect cell function and confound outcomes in targeted cells, and off-target editing risk is an important consideration for the development of a base editing therapeutic; thus, minimizing the off-target activity is desirable for both the investigation and potential future treatment of TNR diseases using base editors. Deaminase variants such as those in ABE8e-V106W (refs. ^65,93^), ABE8.17-m (refs. ^122^) or V106W variants of TadCBEs or CBE6s (refs. ^108,109^) have been shown to reduce Cas-independent editing events of base editors, and alternative delivery methods including localized administration and delivery modalities that facilitate only transient or cell-type-specific expression of base editors may further reduce the physiological burden of off-target editing in vivo^123,124^.

In this study, we have begun the characterization of unintended targets of these repeat base editing strategies, and found that: (1) the level of off-target editing is inversely correlated with the number of mismatches between the repeat-targeting sgRNA and the sequence of the off-target locus, as expected^70–72^; (2) the vast majority of undesired editing occurs in noncoding or intergenic regions of the human genome; and (3) repeat-targeting base editing often leads to the induction of benign single-nucleotide variations that are observed in the general population and synonymous substitutions at protein-coding loci that preserve endogenous protein sequence. The alternative target and off-target sites of repeat-targeting in the human genome observed in this study warrant further comprehensive cell-type-specific longitudinal analyses to evaluate the regulatory risks of accumulated mutations in targeted tissues, to better assess the safety profile of our approaches and whether interrupting pathogenic repeats that underlie TNR diseases may be a viable therapeutic approach in the future. Nonetheless, the approaches and findings developed here should prove useful to elucidate the causality and biological consequences of uninterrupted and interrupted repeat tracts in cultured cells and animal models of TNR diseases.

## Methods

This research complies with relevant ethical regulations. The study protocol was approved by the Broad’s Institutional Biosafety Committee, the Broad’s Institutional Animal Care and Use Committee (IACUC) and relevant IACUC compliance committees at Massachusetts General Hospital.

### Cell culture

Culture of mESCs and HEK293T cells was performed according to previously published protocols^100^. Undifferentiated 129P2/OlaHsd mESC (male) lines^125^ were maintained on 0.2% gelatin-coated plates feeder-free in mESC media composed of Knockout DMEM (Life Technologies) supplemented with 15% defined FBS (HyClone), 0.1 mM nonessential amino acids (Life Technologies), Glutamax (Life Technologies), 0.55 mM 2-mercaptoethanol (Sigma-Aldrich), 1X ESGRO LIF (Millipore), with the addition of 2i: 5 nM GSK-3 inhibitor XV (Sigma-Aldrich), and 500 nM UO126 (Sigma-Aldrich). *FXN*-mESCs were generated for this project (see below). HEK293T cells were purchased from ATCC (CRL-3216) and were maintained in DMEM (Thermo Fisher) supplemented with 10% FBS (Thermo Fisher). Human fibroblasts lines were purchased from the Coriell Institute and were maintained in DMEM (Thermo Fisher) supplemented with 20% FBS (Thermo Fisher). Human fibroblast lines used in this study included: GM07492 (healthy control); GM04855, GM04281 and GM09197 (HD lines); and GM03816 and GM04078 (FRDA lines). All cells were regularly tested for mycoplasma.

For genome editing experiments, cells were seeded 1 d before the experiment to be ~70–80% confluent on the day of transfection and transfected with sgRNA and base editor plasmids at a 1:1 molar ratio using Lipofectamine 3000 (Thermo Fisher) in accordance with the manufacturer’s protocols. For stable integration of plasmids, cells were co-transfected with Tol2 transposase at an equimolar ratio. For antibiotic selection, *FXN-*mESCs were treated with 50 μg ml^−1^ hygromycin B (Thermo Fisher) and/or 6.67 μg ml^−1^ blasticidin S (Thermo Fisher), as indicated, starting 24 h after transfection. Selected cells were allowed to recover and expand before collection. All sgRNA sequences designed for this study are listed in Supplementary Table 27.

### Cloning

Base editor plasmids were constructed by replacing the deaminase and Cas-protein domains of the p2T-CMV-ABE7.10-BlastR (Addgene, cat. no. 152989) plasmid or p2T-CMV-BE4max-BlastR with USER cloning (New England Biolabs)^54^. Individual sgRNAs were cloned into the SpCas9-hairpin U6-sgRNA expression plasmid (Addgene, cat. no. 71485) using BbsI plasmid digest and Gibson assembly (New England Biolabs). Protospacer sequences and gene-specific primers used for amplification followed by HTS are listed in Supplementary Table 27. Constructs were transformed into Mach1 chemically competent *Escherichia coli* (Thermo Fisher) and grown on Luria-Bertani (LB) agar plates, and liquid cultures were grown in LB broth overnight at 37 °C with 100 μg ml^−1^ ampicillin. Individual colonies were validated by Templiphi rolling circle amplification (Cytivia) followed by Sanger sequencing. Verified plasmids were prepared by mini, midi or maxiprep (Qiagen).

AAV vectors for *FXN* GAA editing were cloned by Gibson assembly using NEB Stable Competent *E. coli* (High Efficiency; New England Biolabs) to insert the sgRNA sequence and N-terminal base editor half of ABE8e-dNRCH into v6 Cbh-AAV-ABE-NpuN+U6-sgRNA (Addgene, cat. no. 137177), and the C-terminal base editor half and a second U6-sgRNA cassette into v6 Cbh-AAV-ABE-NpuC (Addgene, cat. no. 137178)^83^. AAV vectors for *HTT* CAG editing were similarly cloned by Gibson assembly using NEB Stable Competent *E. coli* (High Efficiency). The sgRNA sequence and the N-terminal base editor half of EA-evoA-32NLS-NG were cloned into v5 Cbh-AAV-CBE-NpuN+U6-sgRNA (Addgene, cat. no. 137175), and the C-terminal base editor half and a second U6-sgRNA cassette into v5 Cbh-AAV-CBE-NpuC (Addgene, cat. no. 137176)^83^.

### Electroporation

Patient-derived fibroblast lines were obtained from Coriell and cultured in high-glucose DMEM (Thermo Fisher) supplemented with 20% (v/v) FBS. The following lines were used for HD: GM04855 (20/48 CAG), GM04281 (17/71 CAG), GM09197 (18/180 CAG) and GM07492 (15/16 CAG); and for FRDA: GM07492 (8/9 GAA), GM03816 (330/380 GAA) and GM04078 (541/420 GAA). Patient-derived fibroblasts (Coriell) were grown to 80% confluency on a 15-cm plate, washed with PBS (Thermo Fisher), trypsinized using TrypLE Express enzyme (Thermo Fisher) and suspended in 10 ml of media. Cells were transferred to Falcon tubes and centrifuged for 8 min at 150*g* and washed twice in 1 ml of PBS. Each electroporation reaction was assembled with 200,000 cells, 1,000 mg of editor mRNA and 50 pmol of sgRNA (Synthego) and performed with a P2 cell line kit using the DS-150 program (Lonza) on a Lonza 4D nucleofector. Immediately after electroporation, 80 ml of media was added to each well and incubated at room temperature for 10 min before transferring into 1 ml of media in a 24-well plate. Electroporated cells were grown for 5 d or as indicated in the text, with media change every other day. At the end of the experiment, cells were collected, and extracted DNA and RNA (AllPrep DNA/RNA, Qiagen) were used for downstream applications (sequencing, gel electrophoresis and/or droplet digital PCR (ddPCR)).

### Generation of transgenic mESCs

Transgenic mESCs were generated by Tol2-mediated integration of a custom transgene into mESCs. For generation of *FXN*-mESCs, the transgene was generated by PCR amplification of gBlocks (IDT) encoding an *FXN* intron 1 with 30 GAA repeats with 250 base pairs (bp) of flanking sequence on each end of the repeats. Constructs with longer GAA repeats were cloned by PCR amplification of the *FXN* locus from YG8s.300 mouse with the resulting amplicon harboring ~60 GAA repeats. The obtained transgenes were cloned into p2T-CAGGS-MCS-p2A-GFP-PuroR plasmid (Addgene, cat. no. 107186) using Gibson assembly (New England Biolabs) into BamHI cloning site. Constructs were transformed into Stb3 chemically competent *E. coli* (Thermo Fisher) and grown on LB agar plates and liquid cultures were grown in LB overnight at 37 °C with 100 μg ml^−1^ ampicillin. Individual colonies were validated by Templiphi rolling circle amplification (Cytivia) followed by Sanger sequencing. Verified plasmids were prepared by mini, midi or maxiprep (Qiagen). Six-well plates with >10^5^ initial mESCs were transfected with a total of 3.75 μg of *FXN-*30GAA or *FXN-*60GAA transgene together with 3.75 μg of Tol2 plasmid to allow for stable genomic integration using Lipofectamine 3000 and according to manufacturer protocols. Transfected cells were selected with 0.25 μg ml^−1^ Puromycin (Thermo Fisher, cat. no. A1113803) starting the day after transfection for 4 d, before splitting 1:1. After reaching confluency, cells were sorted for the GFP^+^ population, plated with no antibiotic and expanded for at least 7 d. The GFP^+^ enriched population was GFP^+^ sorted again, expanded, selected with Puromycin for at least 7 d and serially diluted onto two 96-well plates. Obtained individual clones were genotyped and the presence of the *FXN* transgene was confirmed by PCR (Supplementary Table 27). The number of transgene integrations was quantified by ddPCR (Supplementary Table 27), and ranged from 4 to 8 integrations for selected *FXN-*30GAA lines and from 6 to 20 integrations for selected *FXN-*60GAA lines. Clones with correct and intact transgenes were further expanded for base editing experiments. Selected cell lines harbored 4, 4 and 2 integrations of the *FXN-*30GAA transgenes and 3, 9 and 10 integrations of the *FXN-*60GAA transgenes, and were used as independent biological replicates in the base editing experiments.

### HTS of gDNA

Library preparation was performed according to previously published protocols^54^. Primers used in this study are listed in Supplementary Table 27. Briefly, we isolated gDNA with the QIAamp DNA mini kit (Qiagen) and used 50–200 ng of gDNA to assess individual locus editing. Sequencing libraries were amplified in two steps. First, to amplify the locus of interest and, second, to add full-length Illumina sequencing adapters using the NEBNext Index Primer Sets 1 and 2 (New England Biolabs) or internally ordered primers with equivalent sequences. PCRs of *HTT* amplicons were performed with Herculase II Fusion DNA Polymerase (Agilent) according to manufacturer protocols with 2–4-min extension time and a total of 24–28 amplification cycles. PCRs of *FXN* amplicons in HEK293T cells were performed using NEBNext Q5 Master Mix (New England Biolabs) with 2-min extension time and 24 PCR cycles. *FXN* locus in patient fibroblasts and mouse tissues was amplified in a two-step nested PCR with (1) UltraRun LongRange Master Mix (9–10 cycles, 5-min extension time; Qiagen) and (2) NEBNext Q5 Master Mix (8–9 cycles, 5-min extension; New England Biolabs). All PCR reactions were supplemented with Betaine (Sigma-Aldrich) at a final concentration of 0.5 M.

Samples were pooled using TapeStation (Agilent) and quantified using a KAPA Library Quantification Kit (Roche). The pooled samples were sequenced using Illumina MiSeq sequencers and Illumina MiSeq Control software (v.3.1). Alignment of fastq files and quantification of editing frequency for individual loci were performed using custom software powTNRka (v.1.0.0, pol. ‘powtorka’–‘a repeat’) described in Supplementary Note 2 (repeat amplicons) or CRISPResso2 (typical amplicons) in batch mode^126^. The editing frequency for each site was calculated as the ratio between the number of modified reads (that is, containing at least one nucleotide conversion or indel) and the total number of aligned reads.

### Quantification of editing

Editing levels were quantified using powTNRka, a custom alignment and analysis software described in Supplementary Note 2 (refs. ^126,127^). From a user’s perspective, powTNRka accepts International Union of Pure and Applied Chemistry nomenclature when computing alignments. In this work, alleles for base editing were specified as YAR (C or T; A; A or G) for *HTT* to capture all possible cytosine base editing events, including those resulting from the opposite-strand deamination, and GRR (G; A or G; A or G) for *FXN* to capture all combinations of adenine base editing events.

### Estimation of editing

Sequencing reads were filtered and aligned using powTNRka. For each sample and condition, all pure GAA triplets and triplets with A•T>G•C interruptions were calculated. These data were used to determine the fraction $\left(f\;\right)$ of all sequenced triplets that contain interruptions. Fraction $f$ was then normalized to the average number of A•T>G•C interruptions $\left({n}_{\rm{i}}\right)$ observed in interrupted *FXN* alleles in the respective sample (Fig. 5c,f and Extended Data Fig. 6b). The resulting factor $(\frac{f}{{n}_{\rm{i}}})$ describes the probability of an A•T>G•C edit occurring at a single GAA triplet across all sequenced reads. This factor was then used to estimate the probability that an *FXN* GAA allele of a specified repeat size $\left(N\right)$ contains at least one interruption (that is, the estimated editing) according to the following formula: $1-{(1-\frac{f}{{n}_{\rm{i}}})}^{N}$.

To estimate the average number of A•T>G•C interruptions in interrupted *FXN* alleles of a specific GAA repeat size, we calculated the average fraction $\left({\;f}_{\rm{i}}\right)$ of each sequenced repeat tract that was edited into interruptions across all interrupted *FXN* alleles. This number was then used to estimate the number of interruptions that can occur in interrupted *FXN* alleles of a specified GAA repeat size $\left(N\right)$ by simple multiplication: ${f}_{\rm{i}}\times N$.

### Quantification of *FXN* expression

RNA from FRDA patient-derived fibroblasts was isolated with the AllPrep DNA/RNA kit or the RNeasy mini kit (Qiagen). Then, 100–500 ng of RNA was used to perform reverse transcription using SuperScript IV (Thermo Fisher) according to the manufacturer’s protocols. The level of *FXN* transcripts was quantified by digital droplet PCR (Bio-Rad) using 1 × ddPCR Supermix for probes (no dUTP (deoxyuridine triphosphate)) (Bio-Rad), complementary DNA equivalent to 10–20 ng of initial RNA input and two sets of primers/probes: human frataxin (Bio-Rad, assay ID: dHsaCNS648692366) and human TBP (Bio-Rad, assay ID: dHsaCPE5058363). Droplet generation, PCR amplification (95 °C for 10-min ramp at 2 °C s^−1^, (94 °C for 30-s ramp at 2 °C s^−1^, 60 °C for 1-min ramp at 2 °C s^−1^) × 49, 98 °C for 10-min ramp at 2 °C s^−1^) and droplet reading were performed on a QX ONE Droplet Digital PCR system (Bio-Rad). ddPCR data were analyzed using QX ONE Software (Bio-Rad).

### Purification of NRCH and NG Cas nuclease proteins

NRCH and NG Cas nuclease proteins were cloned into the expression plasmid pD881-SR (Atum, cat. no. FPB-27E-269). The resulting plasmid was transformed into BL21 Star DE3 competent cells (Thermo Fisher, cat. no. C601003). Colonies were picked for overnight growth in Terrific Broth + 25 μg ml^−1^ kanamycin at 37 °C. The next day, 2 l of prewarmed Terrific Broth was inoculated with overnight culture at a starting optical density (OD)_600_ of 0.05. Cells were shaken at 37 °C for about 2.5 h until the OD_600_ was ~1.5. Cultures were cold shocked in an ice-water slurry for 1 h, at which point l-rhamnose was added to a final concentration of 0.8%. Cultures were then incubated at 18 °C with shaking for 24 h to induce protein expression. Following induction, cells were pelleted and flash-frozen in liquid nitrogen and stored at −80 °C. The next day, cells were resuspended in 30 ml of cold lysis buffer (1 M NaCl, 100 mM Tris-HCl pH 7.0, 5 mM TCEP, 20% glycerol) with five tablets of cOmplete, EDTA-free protease inhibitor cocktail (Millipore Sigma, cat. no. 4693132001). Cells were passed three times through a homogenizer (Avestin Emulsiflex-C3) at ~124.1 MPa to lyse. Cell debris was pelleted by centrifugation at 20,000*g* for 20 min at 4 °C. Supernatant was collected and spiked with 40 mM imidazole, followed by a 1-h incubation at 4 °C with 1 ml of Ni-NTA resin slurry (G Bioscience, cat. no. 786-940, prewashed once with lysis buffer). Protein-bound resin was washed twice with 12 ml of lysis buffer in a gravity column at 4 °C. Protein was eluted in 3 ml of elution buffer (300 mM imidazole, 500 mM NaCl, 100 mM Tris-HCl pH 7.0, 5 mM TCEP, 10% glycerol). Eluted protein was diluted in 40 ml of low-salt buffer (100 mM Tris-HCl, pH 7.0, 1 mM TCEP, 20% glycerol) just before loading into a 50-ml Akta Superloop for ion-exchange purification on the Akta Pure25 fast protein liquid chromatography. Ion-exchange chromatography was conducted on a 5-ml GE Healthcare HiTrap SP HP prepacked column (Cytivia, cat. no. 17115201). After washing the column with low-salt buffer, the diluted protein was flowed through the column to bind. The column was then washed in 15 ml of low-salt buffer before being subjected to an increasing gradient to a maximum of 80% high-salt buffer (1 M NaCl, 100 mM Tris-HCl, pH 7.0, 5 mM TCEP, 20% glycerol) over the course of 50 ml, at a flow rate of 5 ml min^−1^. We collected 1-ml fractions during this ramp to high-salt buffer. Peaks were assessed by SDS–PAGE to identify fractions containing the desired protein, which were concentrated first using an Amicon Ultra 15-ml centrifugal filter (100-kDa cutoff, UFC910024), followed by a 0.5-ml 100-kDa cutoff Pierce concentrator (cat. no. 88503). Concentrated protein was quantified using a BCA assay and determined to be 12.6 mg ml^−1^ (Thermo Fisher, cat. no. 23227).

### CIRCLE-seq off-target editing analysis

Off-target analysis using CIRCLE-seq was performed as previously described^62,104^. Briefly, gDNA from HEK293T cells or NIH3T3 cells was isolated using Gentra Puregene Kit (Qiagen) according to the manufacturer’s instructions. Purified gDNA was sheared with a Covaris S2 instrument to an average length of 300 bp. The fragmented DNA was end-repaired, poly(A)-tailed and ligated to a uracil-containing stem-loop adapter using the KAPA HTP Library Preparation Kit, PCR Free (Roche). Adapter-ligated DNA was treated with Lambda Exonuclease and *E. coli* Exonuclease I, then with USER enzyme and T4 polynucleotide kinase (New England Biolabs). Intramolecular circularization of the DNA was performed with T4 DNA ligase (New England Biolabs) and residual linear DNA was degraded by Plasmid-Safe ATP-dependent DNase (Lucigen). In vitro cleavage reactions were performed with 250 ng of Plasmid-Safe ATP-dependent DNase-treated circularized DNA, 90 nM NRCH or NG Cas9 nuclease protein, Cas9 nuclease buffer (New England Biolabs) and 90 nM synthetic chemically modified sgRNA (Synthego), in a total volume of 100 μl. Cleaved products were poly-A-tailed, ligated with a hairpin adapter, treated with USER enzyme and amplified by PCR with barcoded universal primers NEBNext Multiplex Oligos for Illumina (New England Biolabs), using Kapa HiFi Polymerase (Roche). Libraries were sequenced with 150-bp paired-end reads on an Illumina MiSeq instrument. CIRCLE-seq data analyses were performed using open-source CIRCLE-seq analysis software and default recommended parameters (https://github.com/tsailabSJ/circleseq), using the human genome assembly hg19 as the reference genome. The CIRCLE-seq nominated sites (in hg19) were converted to hg38 to enable downstream analysis with tools that use hg38 as their reference genome. All coordinates in the corresponding Supplementary Tables are provided in hg19, however. Genomic region assignments for the identified off-target hits were performed with HOMER^73^.

### WGS and data analysis

HEK293T cells were seeded in a 96-well plate and transfected at 70% confluence with 66 ng of sgRNA and 200 ng of CBE or ABE base editor in six technical replicates and three biological replicates using Lipofectamine 3000 (Thermo Fisher), as described above. At 3 d after transfection, cells were collected, gDNA was isolated with the QIAamp DNA mini kit (Qiagen) and technical replicates were pooled together. PCR-free library preparation and WGS were performed by the Broad Institute Genomics Platform. Briefly, 350 ng of human DNA was acoustically sheared with an ultrasonicator (Covaris) to obtain 450-bp-long fragments. Libraries were created using a Kapa HyperPrep Plus kit according to manufacturer protocols. All samples were paired-end sequenced (2 × 150 bp) on the NovaSeqX Platform for 160× sequencing coverage. Initial data processing and read alignment were performed by the Broad Institute Genomics Platform. Reads were demultiplexed and aligned to hg38 using DRAGEN (v.3). The obtained WGS data were used to investigate the editing activity of base editors at genomic coordinates nominated in the CIRCLE-seq analyses described above.

All subsequent analyses were performed using the Muhee Cluster high-performance computing cluster (Brigham and Women’s Hospital). pysam (v.0.22.1) was used to analyze editing frequency^128–131^. The editing fraction at CIRCLE-seq-nominated loci was calculated on every sample independently. Genomic loci represented by 30 or fewer sequencing reads were filtered out. Editing (that is, frequency of a CAA•TTG or GAG•CTC, GGA•CCT or GGG•CCC edit) was measured at every amenable nucleotide position at the remaining sites. The untreated control group was used as a reference to exclude nucleotide positions where the background allele frequency was >2.5%, indicating that these variants were already present in the initial unedited cell population and therefore that polymorphisms at these positions are unlikely to have resulted from base editing activity. The editing at each CIRCLE-seq-nominated locus was then assessed by applying a probability algorithm that accounts for editing at each nucleotide position within a given locus to estimate the likelihood of the locus acquiring at least one interruption^54^. While this pipeline can sensitively detect rare base changes at >0.5% editing threshold, including those naturally occurring in cell culture, this lenient filter may overestimate base editing in treated samples. Due to this inherent high technical error, while we generally report editing values using a >0.5% threshold, we apply a ≥5% editing threshold (‘substantial’ editing) for detailed downstream analyses of mutated allele frequencies in WGS data. This ≥5% editing threshold is also used to classify edits as synonymous, nonsynonymous or nonsense.

The MANE.GRCh38.v1.3.refseq genomic dataset^132^ was used to analyze the effect of base editing on amino acid sequence at respective off-target loci for all protein-coding sites. The normal tissue database from the Human Protein Atlas was used to check whether the coding loci or genes are expressed in brain-related tissues^105^. Essential genes were identified with the Cancer DepMap database^74,75^ and the effect of amino acid substitutions on protein folding and function was determined with the AlphaMissense database^76,77^.

### Husbandry of *Htt.Q111* and YG8s mice

All animal procedures were carried out to minimize pain and discomfort, under approved IACUC protocols of the Massachusetts General Hospital. Animal husbandry was performed under controlled temperature (18–23 °C) with humidity 24–60%, and on a 12-h light/dark cycle. Animals were housed in groups of 1–5 individuals per cage, with the same sex and strain. Animals were trans-cardially perfused via thoracotomy using Avertin or euthanized with CO_2_ to collect tissues for DNA and RNA. Both male and female animals were used in the study.

Heterozygous *Htt.Q111* animals were maintained on a C57BL/6J background^133^. gDNA was isolated from tail biopsies using Quick Extract DNA Extraction Solution (VWR, cat. no. QE09050). The litter was genotyped to determine the size of *HTT* CAG repeats in *Htt*.*Q111* allele by PCR using human-specific *HTT* primers (Supplementary Table 27) and Taq PCR Core Kit with Q solution (Qiagen)^134,135^, with the following thermocycling conditions: initial denaturation 95 °C (5 min), 30 cycles of (95 °C (30 s), 65 °C (30 s), 72 °C (90 s)), final extension 68 °C (10 min). PCR products were resolved on either a 1% agarose gel or on an ABI3730xl automated DNA analyzer (Applied Biosystems) to check for the presence of expanded CAG repeats.

The average modal CAG repeat size (measured in the tail) of the mice used in the study was 118 (ranging from 117 to 119) for control and 114 (ranging from 108 to 120) for AAV-CBE-treated animals in the 12-week cohort, and 113 (ranging from 107 to 122) for control and 115 (ranging from 109 to 117) for AAV-CBE-treated animals in the 24-week cohort.

YG8s animals (also known as ‘Tg(FXN)YG8Pook/J’), carrying a single copy of YAC human *FXN* transgene with either 300 or 800 GAA repeats, were maintained on a C57BL/6J genetic background. Animals used were hemizygous for YAC transgene and wild type for endogenous *Fxn*. Pups were genotyped to determine the size of *FXN* GAA repeats by PCR amplification (Supplementary Table 27) using TaKaRa PCR Amplification Kit (Takara, cat. no. R011) along with Q solution (Qiagen)^136^. PCR was conducted in 20-μl reactions containing 40 ng of DNA template using the following program: 3 min at 94 °C; 20 cycles of 20 s at 94 °C, 30 s at 64 °C and 5 min at 68 °C; followed by 9 cycles of 20 s at 94 °C and 5 min at 68 °C, with each subsequent elongation step increased by 15 s; and a final extension step of 7 min at 68 °C. The average modal GAA repeat size (measured in the tail) of the mice used in the study was 355 (ranging from 343 to 378) for control and 369 (ranging from 341 to 413) for AAV-ABE-treated YG8s.300 mice, and 774 (ranging from 687 to 746) for control and 741 (ranging from 746 to 795) for AAV-ABE-treated YG8s.800 mice.

### ICV injections

Neonatal ICV injections were performed as previously described^83,137^. Briefly, the injection was performed with 600 Series Microliter Hamilton syringe (Hamilton, cat. no. 87943). High-titer qualified AAV was obtained through the Viral Vector Core at UMass Medical School and concentrated using Amicon Ultra-15 centrifugal filter units (Millipore), quantified by quantitative PCR (AAVpro Titration Kit v.2, Clontech) and stored at 4 °C until use. For injection, a small amount of Fast Green Dye was added to the AAV injection solution to assess ventricle targeting. *Htt.Q111*, Yg8s or C57BL/6 pups were anesthetized by placement on ice for 2–3 min, until they were immobile and unresponsive to a toe pinch. Up to 4.5 μl of injection mix (3.8 × 10^13^ vg kg^−1^) was injected freehand, with approximately half of the volume into each ventricle on postnatal day 0–2. No overt adverse events were observed and visual inspection during sample collection and dissection of brain tissue did not reveal any obvious brain abnormalities in the animals treated with AAV-BE compared with those treated with AAV-GFP or PBS or untreated animals.

### Nuclear isolation and sorting of tissues

Tissue collection and nuclear isolation were performed as previously described^83^. Briefly, at the endpoint for the experiment, *Htt.Q111* or YG8s mice were euthanized and brain dissections were performed. For isolation of the cortex and striatum, cerebella were separated from the brain postmortem using surgical tweezers. Hemispheres were separated using a scalpel and the cortex was separated from underlying midbrain tissue with a curved scalpel and tweezers. For nuclear isolation, dissected tissue was homogenized using a glass Dounce homogenizer (Sigma, cat. no. D8938) (20 strokes with pestle A followed by 20 strokes with pestle B) in 2 ml of ice-cold EZ-PREP buffer (Sigma-Aldrich, cat. no. NUC-101). Samples were incubated for 5 min with an additional 2 ml of EZ-PREP buffer. Nuclei were centrifuged at 500*g* for 5 min, and the supernatant was removed. Samples were resuspended with gentle pipetting in 4 ml of ice-cold Nuclei Suspension Buffer consisting of 100 μg ml^−1^ BSA and 3.33 μM Vybrant DyeCycle Ruby (Thermo Fisher) in PBS and centrifuged at 500*g* for 5 min. The supernatant was removed, and nuclei were resuspended in 1–2 ml of Nuclei Suspension Buffer, passed through a 35-μm strainer and sorted into 200 μl of Agencourt DNAdvance lysis buffer using a MoFlo Astrios (Beckman Coulter) at the Broad Institute Flow Cytometry Core Facility. All steps were performed on ice or at 4 °C. gDNA was purified according to the Agencourt DNAdvance (Beckman Coulter) instructions for 200-μl volume.

### Fragment analysis of CAG repeat instability

gDNA was isolated from mouse tissues using DNeasy Blood & Tissue Kit (Qiagen, cat. no. 69506). PCR amplification and repeat instability analysis of the *HTT* CAG repeat locus were performed as described above and oligonucleotides are listed in Supplementary Table 27 (refs. ^134,138^). The forward primer was fluorescently labeled with 6-FAM (Applied Biosystems) and the resulting FAM-labeled PCR products, encompassing the *HTT* CAG repeat, were resolved on the ABI3730xl automated DNA analyzer (Applied Biosystems), with either GeneScan 500-LIZ (mouse tissues) or 1200-LIZ (human fibroblasts) internal size standards, and analyzed with GeneMapper v.5 (Applied Biosystems). Frequencies of *HTT* alleles in HD fibroblasts were calculated relative to the predominant allele in the population, with the main allele frequency set to 1. Oligos used for repeat locus amplification are listed in Supplementary Table 27 (refs. ^134,135^). Of note, the amplicons shown in the agarose gel images include the repeat region as well as a total of ~136-bp flanking sequence for technical reasons related to amplification and HTS analysis. The amplicons used for fragment analysis shown in the histograms were generated using primers that include a total of ~80-bp flanking region, thus differing with the amplicon size shown on the agarose gels by ~56 bp.

CAG expansion indices in *Htt*.*Q111* mice were determined for both AAV9-CBE-treated mice and control animals that were not treated with base editor (untreated or vehicle control mice). CAG repeat instability calculations were performed using GeneMapper peak height data, considering only expansion peaks (that is, change in CAG ≥ 0 units, rightward shift on the histogram, CAG expansion index), only contraction peaks (that is, change in CAG ≤ 0 units, leftward shift on the histogram, CAG contraction index) or both expansion and contraction peaks (*I*_CAG_), and using a 5% peak height threshold^113,135^. Only traces with a modal peak height ≥1,000 were used, and only contractions of up to −40 CAG repeats were considered in the analysis. For each trace, change in CAG was determined from modal allele size (‘main allele’) identified in the tail (stable tissue) of the same animal^80,113^. Of note, minor differences in germline CAG repeat size, the rate of somatic instability between individual *Htt*.*Q111* animals, as well as variations in editing efficiencies in AAV9-CBE-treated animals, can result in variability in the instability index within each group. Additional technical factors, including sampling error, PCR bias due to repeat sequence and size differences between samples and DNA quality that affect peak calling in fragment analysis can also impact data quality and interpretation of these analyses.

### Analysis of GAA repeat instability with long gel electrophoresis

Amplification of GAA repeats was performed according to protocols described by Long et al.^136^. The PCR products were run on 1% agarose gel, and the intensity of the bands was quantified by ImageLab and used to normalize the input of PCR product for a Long gel. The normalized inputs of the amplicons were loaded onto a 1% agarose gel in a large horizonal gel electrophoresis system (VWR, cat. no. 730-1112) and run at 70 V (~67 h for 300 repeats and ~120 h for 800 repeats). Long gel was imaged using a Typhoon 5 laser scanner (Amersham) and band intensities were calculated using ImageJ software to generate a repeat length histogram. For each lane, change in GAA size was determined from modal GAA allele determined in the tail (stable tissue) of the same animal, which was also run in the same gel. GAA repeat instability indices were calculated as described above for the *HTT* CAG repeat^113^, using a 10% intensity threshold. These analyses were performed for AAV9-ABEdCH-treated mice and control animals that were not treated with base editor (untreated, saline-treated or vehicle control mice).

### Statistical analysis

Student’s *t*-tests (two-tailed) with Welch’s unequal variances correction were used to compare sequencing and mRNA levels in individual comparisons. Student’s *t*-tests (one-tailed) with Welch’s unequal variances correction were used to compare somatic instabilities (that is, instability index, expansion index and contraction index) in mouse tissues and *FXN* transcript levels in patient cells. One-sample *t*-test and Wilcoxon test were performed to compare editing frequencies quantified by HTS and WGS. Fisher exact *P* value was calculated to compare *FXN* genotypes in the UK Biobank. The *t*-tests and Pearson correlation were performed using GraphPad Prism v.9.4.1 or Microsoft Excel v.16.64. Presented error bars represent standard deviations of ≥3 independent biological replicates, unless indicated otherwise.

To compare editing outcomes in nanopore sequencing data, the Kolmogorov–Smirnov test was used to compare distributions of the number of interruption products observed in each amplicon. For sample *i* containing ${n}_{i}$ reads, we manually computed the empirical distribution function for the number of interruption products, ${F}_{i,{n}_{i}}\left(x\right)$. For each comparison between samples $i,j$, the Kolmogorov–Smirnov test statistic ${D}_{i,\;j}$ was computed manually based on the formula ${D}_{i,\;j}=\mathop{\max }\limits_{x}\left|{F}_{i,{n}_{i}}\left(x\right)-{F}_{j,{n}_{j}}(x)\right|$. *P* values were then computed by numerically minimizing the following function with the BFGS algorithm: ${P}_{{\rm{KS}}}={\mathrm{arg }}\mathop{\min }\limits_{P}{\left({D}_{i,\;j}-\sqrt{-\frac{{n}_{i}+{n}_{j}}{2{n}_{i}{n}_{j}}\times \log \frac{P}{2}}\right)}^{2}\,$

### Reporting summary

Further information on research design is available in the Nature Portfolio Reporting Summary linked to this article.

## Online content

Any methods, additional references, Nature Portfolio reporting summaries, source data, extended data, supplementary information, acknowledgements, peer review information; details of author contributions and competing interests; and statements of data and code availability are available at 10.1038/s41588-025-02172-8.

## Supplementary information

Supplementary Information Supplementary Text, Discussion and Notes 1 and 2.

Reporting Summary

Supplementary Tables 1–24, 27 A master sheet of all supplementary tables from the manuscript (except for Supplementary Tables 25 and 26). The index of all the items is included in the first tab of this file.

Supplementary Table 25 Off-target sites for sgGAA in the nonhuman primate genome (*Macaca mulatta*) identified with CRISPRitz prediction tool.

Supplementary Table 26 Off-target sites for sgGAA in the human genome identified with CRISPRitz prediction tool.

## Source data

Source Data Fig. 1 Statistical source data.

Source Data Fig. 2 Statistical source data.

Source Data Fig. 3 Statistical source data.

Source Data Fig. 4 Statistical source data.

Source Data Fig. 5 Statistical source data.

Source Data Extended Data Fig. 1 Statistical source data.

Source Data Extended Data Fig. 3 Statistical source data.

Source Data Extended Data Fig. 4 Statistical source data.

Source Data Extended Data Fig. 5 Statistical source data.

Source Data Extended Data Fig. 6 Statistical source data.

Source Data Extended Data Fig. 1f Unprocessed gel.

Source Data Extended Data Fig. 6h,i Unprocessed gel.

## Data Availability

The plasmids used in this study are available through AddGene (depositor: David R. Liu, AddGene IDs: 232720–232724, https://www.addgene.org/browse/article/28252668/). DNA sequencing files can be accessed using the NCBI SRA (PRJNA1193010). Other databases used in this study include: human genome assemblies hg19 and hg38, MANE.GRCh38.v1.3.refseq gnomic dataset, UK Biobank data-field 24062, GENCODE mouse reference genome M32 (GRCm39), the Human Protein Atlas, Cancer DepMap and the AlphaMissense database. All data are available in the main text and the Supplementary Information. Source data are provided with this paper.

## Code availability

Code for powTNRka software written for this paper can be found at https://github.com/alvin-hsu/powTNRka. Supporting source code, which includes all scripts needed to reproduce the data analysis, can be found at 10.7910/DVN/8NFSTC (ref. ^139^).
