# Supplementary material for: Base editing of trinucleotide repeats that cause Huntington’s disease and Friedreich’s ataxia reduces somatic repeat expansions in patient cells and in mice
Source: Nat Genet. 2025 May 26;57(6):1437–51. doi: 10.1038/s41588-025-02172-8 (PMC12165863; doi:10.1038/s41588-025-02172-8)
Supplement: Supplementary file 1 — Supplementary Text, Discussion and Notes 1 and 2. [file 41588_2025_2172_MOESM1_ESM.pdf]

nature genetics

Supplementary information https://doi.org/10.1038/s41588-025-02172-8

Base editing of trinucleotide repeats that cause Huntington's disease and Friedreich's ataxia reduces somatic repeat expansions in patient cells and in mice

In the format provided by the authors and unedited

Table of Contents

Supplementary Text 2
Supplementary Discussion 13
Supplementary Methods 15
References 18
Supplementary Note 1. 23
Supplementary Note 2. 37

Other Supplementary Materials for this manuscript include the following:

- Supplementary Tables 1-24 and 27 (provided as a separate file)
- Supplementary Table 25 (provided as a separate file)
- Supplementary Table 26 (provided as a separate file)

Supplementary Text

Synonymous cytosine base editing of CAG repeats in vitro

In rare instances, CBEs can induce G•C>A•T changes upstream of the protospacer[1], likely from deamination of cytosines on the opposing strand just upstream of the sgRNA binding site transiently exposed by breathing of the DNA duplex. As such, opposite-strand editing may be more likely when multiple CBE binding events occur in proximity at the same target site, as with our sgCTG-targeting approach[1]. At glutamine-coding CAG repeats these edits can result in nonsense mutation (CAG to TAG or TAA, “STOP codons”). We observed the lowest top-strand product purity (defined as the ratio of intended edits to nonsense mutations created on the opposite strand) using the AID family deaminases (20:1 for AID-BE4 and 19:1 for AID-BE5, Fig. 1d, Extended Data Fig. 1a), and the highest top-strand product purity in rAPOBEC1 family deaminases that harbor the ‘EA’ purity and efficiency modifications (55:1 for EA-evoA and 63:1 for EA-BE4, Fig. 1d, Extended Data Fig. 1a)[1].

Next, we explored whether CBE architecture changes can improve the efficiency and purity of CAG repeat base editing (Extended Data Fig. 1a). We speculated that a shorter or more rigid Cas–deaminase linker might restrict movement of the deaminase and thereby limit its access to the opposing DNA strand outside of the R-loop and improve top-strand purity[1,2]. We varied the flexible Gly-Ser (GS) linker length between the EA-evoA deaminase and Cas protein from the canonical 32 amino acids (aa) to 28 or 24 aa[2], and also created rigid linkers of varying lengths (22 to 32 aa) by replacing a portion of the GS linker with a nuclear localization signal (NLS; i.e. 22NLS, 25NLS, 28NLS 32NLS), which we reasoned may additionally increase nuclear localization of the base editor to improve editing efficiency (Fig. 1e, Extended Data Fig. 1a)[3,4]. Linker modifications improved top-strand purity by reducing the frequency of alleles with opposite-strand edits that result in nonsense mutations (STOP codons) up to 1.9-fold and NLS insertion into the linker resulted in a significant increase in editing efficiency up to 1.3-fold relative to the original EA-evoA architecture (Fig. 1e, Extended Data Fig. 1a-b). Among all strategies tested, the EA-evoA-32NLS base editor yielded the highest editing efficiency (64±4.8%) as well as the highest top-strand purity (81:1). We selected this editing strategy (hereafter designated CAG-CBE) for further study (Fig. 1e, Extended Data Fig. 1a-b).

Quantification of cytosine base editing in HD patient cells

Our standard HTS pipeline enables sequencing of up to 81 CAG repeats in a single read, which was sufficient to completely sequence the CAG repeat tracts of all tested HD patient alleles except for the long pathogenic allele in GM09197, which is 180 CAGs long. We investigated whether the frequency of repeat interruptions in a partial segment of the repeat is representative of base editing across an entire repeat allele that exceeds the distance that can be covered by short-read HTS, which

would allow us to extrapolate repeat interruption frequencies even in CAG repeat tracts too long for standard short-read sequencing. First, we analyzed the distribution of repeat interruptions across the entire short (20 repeats) and long (48 repeats) *HTT* alleles of CAG-CBE-treated GM04855 cells by direct sequencing in a single HTS read, and found that interruptions are distributed uniformly across the length of the repeat tract with the exception of ~12 nucleotides from the 5'-end and ~6 nucleotides from the 3'-end, that lie outside the base editing window within the nearest fully matched CAG repeat protospacer (Extended Data Fig. 1d). Next, we compared the detected editing efficiency of the long (180 repeats) pathogenic *HTT* allele of GM09197 cells when sequenced from either the 5' or 3' end, which combined cover ~78% of the repeat region (Extended Data Fig. 1e). Sequencing from the 3' end enabled us to span a larger fraction of the repeat allele (81 repeats from the 3'-end compared to 59 from the 5'-end), and both read orientations resulted in a similar detected fraction of interrupted alleles that was proportional to the length of the repeat region that was covered (78±2.0% over background from the 3'-end, compared to 67±4.9% from the 5'-end). We did not detect the formation of nonsense mutations above background levels resulting from opposite-strand editing across any of the three patient or control primary fibroblast lines used in this study (Fig. 1f). Together, these data demonstrate that CBE interruptions are typically distributed uniformly across the repeat tract, and that the observed frequency of alleles containing at least one edited CAG is therefore proportional to the length of the repeat tract sequenced.

Mismatch-dependent CAG-CBE off-target editing in the human genome

Observed CAG-CBE editing at genomic loci decreased as the number of mismatches with the sgCTG spacer increased (Fig. 2c)[5–7], especially when these mismatches were located in PAM-proximal regions of the sgRNA (Fig. 2e). A single mismatch reduced observed editing by 1.8-fold (19±18% across 655 edited loci, compared to 35±18% at 579 perfect-match loci, Fig. 2c), and increased the fraction of CIRCLE-seq nominated loci with no detectable editing by 2.9-fold (5.7 % of perfect-match loci compared to 14% of single-mismatch loci). Editing was more greatly reduced at off-target loci with three or more mismatches; we did not detect editing at 72% of candidate off-target loci with three or more mismatches, and observed an average of 1.5±0.8% editing at the remaining 928 of 3,335 loci nominated by CIRCLE-seq (Fig. 2c).

Our WGS detection pipeline may overestimate CAG-CBE activity throughout the genome due to sequencing error, allelic variation in HEK293T cells, and our data processing filters that were designed to maximize the sensitivity of off-target detection (Methods)[1,8]. Therefore, to further validate our WGS findings, we characterized editing at *HTT* and six polyglutamine-coding alternative targets of CAG-CBE and at the top 16 CIRCLE-seq nominated protein-coding off-target loci that harbor up to

three mismatches with the sgCTG spacer and that showed ≥1% editing by WGS. We quantified C•G>T•A interruptions in CAG-CBE-treated HEK293T cells and performed targeted-amplicon HTS sequencing to detect editing with high sensitivity. Concordant with the WGS results, a single mismatch reduced editing efficiency by ~2.7-fold (21±18% across seven loci, compared to 57±10% across seven perfectly matched targets), and two or more mismatches largely abolished CBE activity (~5.6-fold reduced, median 2.8%, Fig. 2f). Overall, our WGS pipeline produced results similar to single-amplicon analysis but overestimated off-target editing at some loci (one-sample t and Wilcoxon test *P*=0.0045, Fig. 2g), supporting the ability of the WGS pipeline to filter genome-wide CIRCLE-seq nominated off-target loci for subsequent confirmation by targeted amplicon HTS.

In silico off-target prediction of CAG repeat base editing

We also assessed the potential off-target burden of our base editing strategy in a non-human primate model by *in silico* off-target prediction using CRISPRizt[9] in the macaque (*Macaca mulatta*) genome using a mismatch tolerance of six, as >98% of all candidate off-target loci detected by CIRCLE-seq for this strategy contained up to six mismatches. This analysis identified 193,751 loci that could potentially be targeted by the sgCTG spacer in complex with Cas9-NG in macaques (Extended Data Fig. 2a, Supplementary Table 10). Similarly, CRISPRitz prediction of off-targets in the human genome revealed 173,873 putative off-target loci (Extended Data Fig. 2b, Supplementary Table 11). While the computationally predicted number of off-targets were comparable between macaques and humans, these numbers exceed the empirically determined number of off-targets in the human genome by two orders of magnitude compared to either our CIRCLE-seq biochemical analysis (5,706) or our WGS analysis in base edited HEK293T cells (2,743). Notably, only 4,157 of the CIRCLE-seq, and 2,186 of the WGS-confirmed sites in the human genome (editing >0.5%) overlapped with the *in silico* predictions, suggesting that ≥98% of CRISPRitz predictions are false-positives with respect to either of these empirical assays (Fig. 2e, Extended Data Fig. 2a-c). Additionally, the *in silico* prediction failed to capture 27% of CIRCLE-seq nominated loci and 20% of WGS empirically determined off-targets in base edited cells, indicating that these models exhibit both a high false-positive and false-negative rate.

To provide further insight into the potential off-target editing burden of our sgCTG strategy in a non-human primate (NHP) model, we extrapolated findings from our comparative *in silico* prediction and empirical human genome off-target editing analysis to the CRISPRitz off-target predictions for the macaque genome, based on the number and position of mismatches of each off-target site with the sgRNA. Assuming that the fraction of true positives per mismatch category will be similar across species, we transformed the number of CRISPRitz-predicted off-targets in macaques per mismatch bin based on the ratio of WGS detected off-targets relative to the number of corresponding CRISPRitz

predicted sites in that bin in the human genome, and then applied the same heatmap coloring from our empirical human WGS data (Fig. 2e) to each corresponding bin of the "WGS transformed" macaque data to indicate the potential mean editing that might be observed at those loci (Extended Data Fig. 2c). These representations are highly speculative and intended only to suggest the possible extent of off-target burden that might be expected in an NHP genome; further empirical studies comparing cross-species base editing off-target activity are needed to support the validity of such statistical extrapolations. In sum, these findings underscore that computational predictions can substantially overestimate the off-target burden while also missing off-target genome editing events of potential biological significance that can be detected by experimental off-target detection pipelines, emphasizing both the need for improved computational predictive models and for empirical validation of off-target events.

Quantification of cytosine base editing at pathogenic HTT alleles in vivo

Our HTS workflow does not allow high-fidelity sequencing of the entire length of the CAG repeat tract of *Htt.Q111* alleles. Sequencing up to 81 repeats from either the 5' end and 3' end, however, showed a similar frequency of interrupted alleles (<1.4-fold difference, Extended Data Fig. 3b), concordant with editing observed across *HTT* alleles in HD patient-derived fibroblasts (Extended Data Fig. 1e). These data suggest that CAG-CBE editing is uniformly distributed throughout the CAG repeat (Extended Data Fig. 1d), and thus sequencing from either end of the repeat tract enables estimation of overall editing in *Htt.Q111* alleles.

CAG somatic repeat expansion in Htt.Q111 mice

CAG repeats in *Htt.Q111* undergo age-dependent and tissue-specific somatic expansion, with CAG repeat length remaining largely stable in the mouse tail (Fig. 3g, Extended Data Fig. 3c-d), while undergoing significant expansion in the striatum and cortex (Fig. 3h-i, Extended Data Fig. 3e-f). In bulk tissue samples, the somatic instability that occurs in a minority of alleles can therefore be observed as a deviation of repeat tract lengths compared to the predominant (main) allele size in that sample (Fig. 3g-i, Extended Data Fig. 3d-f). We quantified the average deviation of CAG repeat lengths from the CAG size in the main allele ("CAG instability index") in AAV9-CBE treated and control *Htt.Q111* mice (untreated and vehicle controls, Fig. 3d) in various bulk tissue samples (Fig. 3j, Extended Data Fig. 3c), using a previously established method (Methods)[10,11]. We compared the CAG size variation of *Htt.Q111* alleles in the cortex and striatum, which are targeted by AAV9-CBE and undergo somatic CAG expansion, to the tail which is neither transduced nor undergoes repeat expansion.

CBE-mediated CAA interruption in vivo promotes repeat contractions

Treatment with AAV9-CBE significantly reduced the average size of CAG repeats of *Htt.Q111* alleles in somatic tissues, as measured by the CAG instability index. Tissues that do not exhibit somatic instability, such as the tail, maintain a uniform CAG allele size (Fig. 3g, Extended Data Fig. 3c-d), while tissues that undergo somatic instability exhibit measurable expansion of CAG alleles (Fig. 3h-i, Extended Data Fig. 3e-f). Although this expansion phenotype was seemingly diminished in the cortex and striatum of AAV9-CBE treated animals, close inspection of edited allele sizes revealed that many repeats were reduced in length (Extended Data Fig. 3c).

The classical approach to calculating tandem repeat instability uses the "instability index", which does not distinguish between the relative contributions of expanded and contracted alleles when determining the mean deviation in CAG length[10]. To quantify these changes separately, we calculated indices for each and determined that AAV9-CBE treatment mildly reduces somatic repeat expansion (CAG expansion index $I_{CAG(e)}$) in the striatum at 12 weeks ($I_{CAG(e)}$ = -0.7±0.1 repeats, Welch's one-tailed t-test P=0.0003) and 24 weeks post-injection ($I_{CAG(e)}$= -2.1±0.2 repeats, Welch's one-tailed t-test P<0.0001), but not in the cortex (Extended Data Fig. 3c). Interestingly, base editing at pathogenic CAG repeats contributed to substantial repeat contractions in the edited tissues (CAG contraction index $I_{CAG(c)}$). At 12 weeks post-injection, we observed a reduction in CAG repeat length in *Htt.Q111* alleles from the cortex ($I_{CAG(c)}$ = -2.6±0.6 repeats, Welch's one-tailed t-test P=0.0028) and striatum ($I_{CAG(c)}$= -3.2±0.8 repeats, Welch's one-tailed t-test P=0.0048) which endured over time, reaching $I_{CAG(c)}$= -4.5±1.2 repeats in the cortex (Welch's one-tailed t-test P=0.007) and $I_{CAG(c)}$= -4.5±1.0 repeats in the striatum (Welch's one-tailed t-test P=0.0015) at 24 weeks post-injection, compared to control animals (Extended Data Fig. 3c). Contractions of repeats can be particularly beneficial in cases in which repeat lengths surpass the pathogenic threshold, are inherently toxic, and would not become non-pathogenic simply from reduced somatic expansion[12,13].

Byproducts of CBE-mediated CAA interruption in vivo

Unstable long TNR alleles are known to diverge in length by both expansion and contraction, and may natively undergo -1 and +1 frameshifts that result in polyalanine (poly-A) and polyserine (poly-S) products in a large fraction of alleles (~10–40% of 51Q alleles in prior reports)[14–16]. Since targeted contraction of *HTT* alleles using Cas9 nucleases has previously been shown to induce frameshifting indels[94] in up to ~75% of *HTT* alleles with 41 CAG repeats[17], we investigated whether the allele contractions we observed in AAV9-CBE treated *Htt.Q111* mice are correlated with the acquisition of frameshifted alleles. At 12 weeks post-injection, we observed frameshifts in 3.8±1.5% and 3.3±0.8% of *Htt.Q111* alleles from the cortex and striatum, compared to 0.1±0.1% and 0.0±0.1%, respectively, in untreated animals (Extended Data Fig. 3g). These frameshifted alleles modestly

increased over time, reaching 4.1±1.4% in the cortex and 4.0±1.3% in the striatum by 24 weeks, while remaining relatively low in somatic tissues of untreated animals (0.0±0.1% and 0.1%±0.0%, respectively, Extended Data Fig. 3g). We determined that the frameshifting indels are typically found in interrupted alleles; 94±4.5% and 94±3.4% of the frameshifted alleles in the cortex and striatum, respectively, also contained CAA interruptions (Extended Data Fig. 3g), while 8.5±1.8% and 10±1.8% of interrupted alleles in the cortex and striatum, respectively, contained frameshifting indels (Extended Data Fig. 3h). Frameshifts resulted from the deletion of G, AG, or A nucleotides in 38±3.7%, 32±3.6%, and 12±3.0% of frameshifting indels respectively (Extended Data Fig. 3i), and frameshifting indels were 3.8-fold more abundant at the unstable, pathogenic *HTT* allele compared to the short allele (10±2.9% and 2.6±0.7%, respectively, Extended Data Fig. 3j). It is tempting to speculate that the increase in mismatch repair activity at long CAG repeats increases the error-prone repair of base edited nucleotides, resulting in frameshifting indels that lead to knockout of the pathogenic allele[17].

In vivo DNA and RNA off-target editing analysis of a CAG-CBE

In addition to the off-target analysis in cultured human cells described above, we also assessed the DNA and RNA specificity of AAV9-CBE in treated *Htt.Q111* mice. First, we performed CIRCLE-seq to identify putative Cas9-NG dependent DNA off-target loci of our sgCTG-targeting strategy in mouse genomic DNA isolated from NIH3T3 cells. We then classified the resulting putative off-target loci based on (1) the identity of the targeted region and (2) the number of mismatches with sgCTG, as before (Extended Data Fig. 3k-l, Supplementary Tables 12-13). Among 9,136 CIRCLE-seq nominated off-target candidate sites, 836 (9.2%) map to protein-coding exons in the mouse genome (Extended Data Fig. 3k), 41% map to non-coding gene regions, and 50% are located at intergenic regions. ~15% (123) of putative protein-coding off-targets are alternative targets that are a perfect match for sgCTG (Extended Data Fig. 3l). Thus, the majority of identified off-target sites harbor mismatches that reduce sgRNA binding and base editing efficiency relative to the on-target locus, as demonstrated by our genome-wide analysis of CBE activity and amplicon-based off-target characterization in the human genome (Fig. 2c-f) [5–7]. Further filtering by WGS and validation by single-amplicon HTS in edited mouse cells is required to determine the true frequency of genome-wide CAG-CBE edits and potential biological consequences of off-target cytosine base editing in the mouse genome.

To determine the effect of CAG-CBE on alternative target and off-target gene expression *in vivo*, we performed whole transcriptome analysis on cortical samples of *Htt.Q111* mice treated with either AAV9-CBE+AAV9-GFP or AAV9-GFP at 12 weeks of age. Global transcriptional expression levels were unchanged between the CAG-CBE-treated and control groups (correlation r= 0.97 across 134,701 transcripts, Extended Data Fig. 3m). We identified 782 genes with minor expression changes in CAG-CBE-treated mice compared to vehicle controls (711 upregulated and 71 downregulated

genes, $-1<\log_2 FC<1$, $-\log_{10} P_{adj}>2$, Supplementary Table 14), of which only 3.2% corresponded to CIRCLE-seq nominated off-targets (21 upregulated and 4 downregulated), suggesting that the majority of observed expression changes may relate to *in vivo* base editor expression in general, and are not a direct result of CAG-CBE base editing *in vivo*.

Cas-independent RNA off-target activity of CBEs can lead to low but detectable C-to-U conversion in cells and in mouse embryos[18,19]. We investigated RNA off-target editing *in vivo* in *Htt.Q111* mice treated with AAV9-CBE+AAV9-GFP compared to AAV9-GFP treated controls at 12 weeks post injection[97,98]. Whole transcriptome sequencing revealed low-levels of transcriptome-wide C-to-U accumulation in cortical cells of AAV9-CBE+AAV9-GFP treated mice compared to endogenous background levels observed in AAV9-GFP treated animals (0.004% above the background, Welch's two tailed t-test, P=0.0182, Extended Data Fig. 3n).

Collectively, these data demonstrate that cytosine base editing of CAG repeats efficiently introduces codon interruptions at repeat expansion loci of about a dozen severe TNR diseases, with a proclivity for editing longer repeat alleles. This work demonstrates the protective role of induced synonymous CAG-to-CAA interruptions on expansion of pathogenic *HTT* alleles *in vivo*, and shows that CAG-CBE activity mainly results in silent or non-coding sequence changes at genomic off-target loci. These findings raise the possibility of countering the progression of CAG repeat expansion disorders by base editing of the repeat sequences, with the important caveat that the highly repetitive nature of the target sequence will result in many alternative target and off-target edits that must be carefully characterized before the therapeutic relevance of such a strategy can be understood.

Characterization of GAA expansions in the UK Biobank

To characterize the protective role of interruptions in individuals with pathogenic GAA repeat expansions, we analyzed the *FXN* locus for all genomes of the UK Biobank[20] in which this locus was detected using ExpansionHunter[21] (490,382 genomes). In these data, 31 individuals (~1 in 15,800) had biallelic expansions of GAA beyond the threshold considered pathogenic for FRDA (≥66 repeats)[22], including four individuals with a confirmed diagnosis of hereditary ataxia (G11 ICD-10 code). We then used REViewer[23] to generate read visualizations of the 31 pathogenic-range genotypes and manually assessed genotype quality and GAA repeat sequence purity of these alleles (Table 1, Supplementary Table 1, Supplementary Note 1). We found that 10 of the 31 individuals (32%) with pathogenic *FXN* loci carried two completely pure or near-completely pure GAA repeat tracts, including all four individuals with a G11 hereditary ataxia diagnosis (Fisher exact p-value = 0.0067). The remaining 21 individuals had clear evidence of GAG interruptions in one or both *FXN* alleles with expanded GAA repeats (Table 1, Supplementary Table 1, Supplementary Note 1), yet no

evidence of ataxia or neurologic disease. These findings further support the hypothesis that repeat interruptions reduce the penetrance of pathogenic GAA expansions in FRDA[24–26].

Adenine base editing of GAA repeats at FXN alleles in FXN mESCs

We generated mouse embryonic stem cell lines (mESCs) harboring the human *FXN* intron 1 locus with long GAA repeats (Methods)[27,28] and directly measured ABE-dCas9 editing at up to 50 GAA repeats in these cells. We found that ABE8e fused to dCas9-NRCH greatly outperformed other ABEs by up to 4.2-fold (Fig. 4c). Moreover, we observed that the editing efficiency of GAA repeats generally increased with the length of the repeat tract (23±0.2% in *FXN*-30GAA-mES compared to 32±3.7% in *FXN*-60GAA-mES, Fig. 4c), while maintaining a median of one interruption per edited allele and an even distribution of interruptions across the length of the repeat tract (Extended Data Fig. 4a-b). The increased binding opportunity for base editors at longer repeat tracts likely results in higher fraction of interrupted alleles. Notably, the frequency of triplet interruptions (GAG, GGA, and GGG) differed between the *FXN* allele in HEK293T cells containing nine GAA repeats and the *FXN*-mES cell lines with longer repeat tracts (Extended Data Fig. 4c). Editing in HEK293T cells predominantly resulted in GAA-to-GGG edits (57±1.2% of interruptions), while most interruptions at the longer GAA alleles in *FXN*-mES cell lines were GAA-to-GGA changes (76±2.1% and 78±0.8% of interruptions, respectively, Extended Data Fig. 4c).

GAA-ABE off-target editing in the mouse genome

To investigate the genome-wide Cas-dependent activity of GAA-ABE in the mouse genome, we performed CIRCLE-seq analysis on mouse genomic DNA from NIH3T3 cells[29] using purified ribonucleoprotein (RNP) complexes containing Cas9-NRCH nuclease and the sgGAA sgRNA, as previously described[29,30]. We classified CIRCLE-seq hits based on HOMER and the number of mismatches with sgGAA (Extended Data Fig. 4d-e)[31], as before for genome-wide CBE activity analysis. We identified 51,658 putative GAA-ABE targets in the mouse genome (Supplementary Table 17-18). In the mouse genome, a far greater number (24,354) and proportion (56%) of CIRCLE-seq nominated hits perfectly match the sgGAA sequence compared to the human genome (9-fold and 9-fold higher in mouse, respectively, Fig. 4d-e, Extended Data Fig. 4d-e), and 673 loci (1.3%) mapped to protein-coding exons (Extended Data Fig. 4e, Supplementary Tables 17-18). These data suggest that GAA-ABE may target many GAA repeats, predominantly at non-coding loci, throughout the human and mouse genomes.

Mismatch-dependent GAA-ABE off-target editing in the human genome

Similar to WGS analysis of our CAG-CBE strategy, we observed that genome-wide editing by GAA-ABE decreased with an increasing number of mismatches with the sgGAA spacer (Fig. 4f), in particular when these mismatches were located in PAM-proximal regions of the sgRNA (Fig. 4g)[5–7]. A single mismatch reduced editing by 1.5-fold (12±9.7% across 2,421 edited loci, compared to 17±8.2% at 2,497 perfect-match loci, Fig. 4f-h), and increased the fraction of CIRCLE-seq nominated loci with no detectable editing by 1.8-fold (7.7% of perfect-match loci compared to 13% of single mismatch loci). For loci with three or more mismatches, the fraction of unedited sites increased to 61% (18,915 loci) and we observed an average editing of 1.3±0.3% at the remaining 12,335 of 31,250 CIRCLE-seq nominated loci (Fig. 4f).

Validation of WGS-identified GAA-ABE off-target loci with single-amplicon sequencing

To validate our WGS findings, we selected 63 GAA-ABE targets, including 37 top-ranking protein-coding loci and 26 non-coding loci, among which 14 loci are alternative targets that perfectly match the sgGAA spacer nominated by CIRCLE-seq and 49 loci are off-targets containing up to four mismatches (Supplementary Table 15). We performed single-amplicon sequencing to sensitively detect editing by HTS and quantified A•T>G•C interruptions in GAA-ABE-treated HEK293T cells. Similar to the WGS findings, a single mismatch reduced editing by ~1.2-fold compared to perfectly matched loci (19±12% across 13 loci, compared 22±12% across 14 perfect match loci), two mismatches reduced editing efficiencies by ~3-fold (7.6±9.9% across 20 loci), and three or more mismatches largely abolished GAA-ABE activity to background levels (~37-fold reduced, 0.6±1.9%, Fig. 4h). While WGS overestimated editing at some loci for both CAG-CBE and GAA-ABE (One-sample t and Wilcoxon test P=0.0045 and P<0.0001 respectively, Fig. 2g, Extended Data Fig. 4f), amplicon sequencing largely corroborated findings of our WGS analysis, confirming that WGS is a reliable method to filter genome-wide putative base editing off-targets identified by CIRCLE-seq with high sensitivity for subsequent validation by single-amplicon HTS.

In silico off-target prediction of GAA repeat base editing

Similar to our *in silico* off-target analyses for CAG-CBE, we assessed the potential off-target activity of the sgGAA spacer in a non-human primate model using CRISPRitz[9] for the macaque genome, with a mismatch tolerance of six as >99% of candidate off-target loci nominated by CIRCLE-seq contained up to six mismatches for this strategy. This analysis identified over 3,000,000 potential off-target loci that could be targeted by the sgGAA spacer in macaques in conjunction with Cas9-NRCH, which recognizes both NA and NG PAMs (Extended Data Fig. 4g, Supplementary Table 25). This large number of off-targets can be attributed to the abundance of G/A-rich islands that are predominantly found at non-coding regions, such as primate-specific Alu elements that frequently

contain mixtures of GAA, GGA, GAG, and GGG triplets[32]. Using CRISPRitz, we also predicted over 2,800,000 potential off-target sites in the human genome (Extended Data Fig. 5a, Supplementary Table 26), again exceeding our empirically determined number of human genomic off-targets by either CIRCLE-seq (41,992) or WGS (20,559) by two orders of magnitude, as with the CBE strategy. These *in silico* predicted sites included 33,305 loci nominated by CIRCLE-seq and 17,226 that were confirmed by WGS, indicating a false-positive rate of ≥98% with respect to either of these empirical assays, and a false-negative rate of around 20% (21% for CIRCLE-seq nominated loci and 16% for WGS determined off-targets).

To provide a more realistic estimation of the off-target burden that may be expected in macaque base edited cells, we again extrapolated the ratio of edited *versus* CRISPRitz predicted off-target loci in the human genome per mismatch bin to the CRISPRitz predicted off-targets in macaque, and then imposed the mean editing for each bin that we observed from our HEK293T cells WGS analysis on the macaque bins (Extended Data Fig. 5b). Further studies to compare off-target activity between human and non-human primate cell models are needed to support the validity of these extrapolated graphical representations of off-target burden.

Collectively, our findings suggest that computational predictions for these trinucleotide repeat targets significantly overestimate the off-target burden while frequently missing off-target events of potential importance, highlighting the need for improved computational predictive models and empirical validation of putative off-target candidates.

Adenine base editing of GAA repeats in FRDA patient cells

Our short-read HTS pipeline enables sequencing of up to 80 GAA repeats, which does not allow a direct read out of the entire pathogenic repeat region of FRDA patient *FXN* alleles that are >300 GAAs long. Since base editing repeat interruptions are typically distributed uniformly across the repeat tract (Extended Data Fig. 1d, Extended Data Fig. 4b) and we detect GAA-ABE editing at <24% of the total length of GAAs in the GAA repeat tract in FRDA fibroblasts, the reported values in Figure 5a likely greatly underestimate the true editing frequency of GAA-ABE at long GAA alleles. We designed a computational model, informed on the editing we detect by short-read HTS, to estimate GAA-ABE editing across the entire length of long GAA repeat tracts (Methods). We validated that our estimation model accurately predicts the edited fraction of *FXN* alleles in control fibroblasts with 8/9 GAAs, which are fully sequenced by our short-read HTS pipeline (18±5.9% estimated editing, versus 20±7.0% observed editing efficiency, n.s. Welch's two-tailed t-test, Fig. 5b). In GM03816 and GM04078 FRDA patient-derived fibroblasts, we sequenced 14–23% (~76 GAA repeats on average) of total GAA repeats and detected 31±12% and 30±9.5% interruption of *FXN* alleles, while our model estimates a true interruption frequency of 84±15% and 91±8.1% of alleles, respectively (Fig. 5b).

Next, we used the estimation model to predict the number of A•T>G•C interruptions introduced by GAA-ABE at pathogenic *FXN* alleles. The model estimated that in FRDA fibroblasts with ~330/380 GAAs (GM03816) or ~541/420 GAAs (GM04078), the edited *FXN* alleles acquire on average 14±4 and 19±3 interruptions, respectively (Extended Data Fig. 5c). The predicted number of interruptions in control fibroblasts with short *FXN* alleles that are fully sequenced by our short-read HTS pipeline was almost identical to the observed frequency (2±0.2 estimated average *versus* 2±0.3 observed average interruptions, n.s. Welch's two-tailed t-test, Extended Data Fig. 5c).

Adenine base editing of FXN alleles in vivo

To assess how A•T>G•C interruption of long pathogenic GAA repeats affects repeat expansion at *FXN* alleles *in vivo*, we designed an AAV strategy to deliver ABE-GAA to FRDA mouse models. We packaged ABE8e-dNRCH and sgGAA into a dual AAV delivery system using AAV serotype 9 (v6 AAV9-ABE, Fig. 5d)[33], which has high tropism for FRDA-relevant CNS and peripheral tissues that undergo repeat expansion[34–36]. First, we determined AAV9 transduction efficiency in the CNS of YG8s mice by PND0 neonatal ICV injection using 0.4–2.5x10^{10} vg per mouse AAV9-GFP[33,37], and found that cortical transduction largely saturates around 1.5x10^{10} total vg, resulting in an average transduction efficiency of 64±4.2% (Extended Data Fig. 6a)[33,38–40].

Our previous *in vitro* editing analyses suggest that GAA-ABE-mediated interruption of GAAs occurs largely uniformly throughout the repeat tract, with a potentially positive correlation between repeat length and editing efficiency (Fig. 4c, 5b). To determine the editing frequency in YG8s mice, we sample only a subset (up to 80) of GAA repeats in the *FXN* locus by short-read HTS. The detected frequency of *FXN* editing in the small sample of GAA repeats (~27% and ~10% in YG8s.300 and YG8s.800, respectively) underrepresents the true frequency of edited alleles across the full repeat tract (Fig. 5f). We used our estimation model described previously (Methods) to extrapolate editing across the entire repeat region in YG8s.300 and YG8s.800 mice, and to predict the average number of A•T>G•C interruptions per pathogenic GAA repeat tract. Based on this method, we estimated that four weeks after treatment, the frequency of *FXN* repeat disruption in cortical alleles of YG8s.300 mice reached ~22±13% (of which 5.4±2.6% was directly observed) (Extended Data Fig. 6b).

Adenine base editing of FXN alleles in vivo in peripheral tissues

P0 ICV injection of AAV9 primarily enables the transduction of CNS tissues, though it may also target some peripheral tissues[41]. To this end, we measured GAA-ABE editing in CNS and peripheral tissues that are highly targeted by AAV9 and potentially relevant to FRDA, including heart (18±2.5% estimated, 2.8±0.6% observed), liver (24±11% estimated, 4.7±1.9% observed), striatum (17±4.6% estimated, 2.8±1.1% observed) and brainstem (36±9.3% estimated, 2.3±0.9% observed) in YG8s.300 mice (Extended Data Fig. 6c). Collectively, these data support that AAV9-ABEdCH enables broad

transduction and efficient *in vivo* editing of long *FXN* GAA repeats in YG8s mouse tissues undergoing somatic expansion.

Quantification of adenine base editing with long-read sequencing

Our estimation model relies on base editing information obtained from up to 80 GAA repeats from the 3' end of the GAA repeat tract and operates under the assumption that the probability of base editing events remains constant throughout the entire repeat tract, barring ~6 nucleotides from the 5'-end and ~12 nucleotides from the 3'-end that lie outside the base editing window within the nearest fully matched sgGAA protospacer[42]. We explored an alternative empirical method to detect interruptions in long *FXN* alleles to validate our model using long-read nanopore sequencing[43]. The comparatively high error-rate of nanopore sequencing technology, compared to short-read HTS, does not allow for direct determination of the true editing profile in long GAA repeats or direct quantification of edited *FXN* alleles[43,44]. However, comparative analysis of *FXN* alleles from AAV9-ABEdCH-treated and untreated mice allowed us to calculate the cumulative probabilities of detecting a given number of A•T>G•C interruptions at these alleles, enabling us to better characterize the true interruption profile in edited alleles (Kolmogorov-Smirnov test, Extended Data Fig. 6g). The probability distributions for detecting A•T>G•C changes in *FXN* alleles from the cortex of uninjected YG8s.300 mice show that 14±0.0% of alleles have at least five interruptions at their GAA repeats (Extended Data Fig. 6g), compared to 26±2.2% in AAV9-ABEdCH-treated mice. Moreover, over 10% of *FXN* alleles (11±1.4%) in AAV9-ABEdCH-treated mice contained ≥25 interruptions per GAA repeat tract (Extended Data Fig. 6g). Of interrupted *FXN* alleles, the majority (86±1.3%) acquired <34 interruptions per ~300 GAAs (interruption:GAA ratio of 1:9), in agreement with our HTS data and modeling outcomes that predict up to 9 A:T>G:C interruptions per edited repeat tract (interruption:GAA ratio of 1:9, Extended Data Fig. 6e-f). These long-read sequencing data support the findings of our short-read HTS results and estimation model, supporting that *in vivo* AAV9-ABEdCH delivery enables GAA-repeat interruption throughout the length of the repeat tract.

Supplementary Discussion

Genome editing approaches for TNRs have thus far primarily focused on nuclease-mediated removal of pathogenic repeats[45–52]. While such strategies can benefit cell and animal models of TNR disease, the induction of DSBs can result in large deletions and chromosomal rearrangements, especially when induced simultaneously at multiple genomic loci. In contrast, base editors convert nucleotides without requiring DSBs and induce much lower levels of genomic instability and indel byproducts, and may thus have a lower mutagenic burden on targeted cells than some nuclease-based approaches[50–52]. Several advanced genome editing tools, such as prime editing[53–55], which can

in principle precisely remove pathogenic repeats at a target genomic site, and targeted epigenetic modification to modulate gene expression[56–58] offer promising alternatives for correcting pathogenic mutations at TNR loci. While these strategies address different aspects of TNR disease pathology than those explored in this study, similar to base editors, these methods minimize the formation of DSBs and indels, and may thus provide future therapeutic options and further insights into the biology of TNR diseases.

This study demonstrates that introducing naturally occurring interruptions in pathogenic trinucleotide repeats in animal models of two TNR diseases can reduce somatic repeat expansions, a key neurological feature of these repeat tracts that is known to drive disease progression, inheritance, and anticipation. In a recent study, Choi *et al*. investigated the stabilizing effect of CAA interruptions in pathogenic-length CAG repeats[59]. While the base editing strategies reported in their study did not reduce instability of *HTT* alleles *in vitro* or *in vivo*, possibly due to insufficient base editing efficiency, they reported a HD transgenic mouse model with germline-encoded CAA interruptions at the *HTT* allele, and demonstrated that these mice do not exhibit the somatic poly-Q expansion that is seen in HD mice with pure CAG *HTT* alleles of a similar length. These findings support the stabilizing effect of using base editing to interrupt repeats that we observed in this study.

A single base editor that targets a specific triplet repeat, such as CAG•CTG or GAA, could in principle be applied to a variety of disorders caused by that sequence expansion, regardless of the TNR-associated gene that harbors that repeat. The repeat-targeting CBE and ABE strategies described here induce efficient repeat interruption at pathogenic alleles, along with a wide range of alternative target and off-target loci. While we targeted CAG•CTG or GAA repeat expansions in this study, similar base editing approaches could be developed for other repeat sequences to provide valuable insights into the biology of repeat instability in other nucleotide repeat disorders.

The repeat-targeting approaches described in this study would benefit from improvements to both efficiency and specificity. The primary limitation of these approaches is the high abundance of unintended editing events across the genome. Although base editing often results in silent single base-pair changes at these off-target loci, evaluating the potential downstream consequence of these outcomes is challenging. However, ~90% of the identified genomic off-targets contain one or more mismatches with the sgRNA, suggesting that improved base editor sequence fidelity could significantly reduce the burden from off-target editing. Additionally, improving the activity of CBEs such as recently described TadCBE and CBE6 variants[60,61] could enable the use of non-nicking base editors for CAG•CTG, as with our GAA editing strategy, which would further reduce the potential risk of DSB formation.

This study demonstrates that dual-AAV delivery of base editors can achieve high efficiency *in vivo* editing in mouse models of human diseases that is sufficient to ameliorate pathogenic somatic repeat expansion in disease-relevant tissues[38,62,63]. We recently reported a single-AAV system for *in*

vivo adenine base editing that uses size-minimized AAV vector components and small Cas protein domains[64]. Currently, these single-AAV base editors are compatible with only a subset of PAM sequences that do not include those needed to target CAG or GAA repeats. Ongoing efforts to expand the PAM tolerance of small Cas proteins may enable single-AAV packaging of repeat-targeting editors in the future, potentially improving *in vivo* targeting of disease-relevant tissues and lowering the total required viral dose[65].

Supplementary Methods

Whole transcriptome RNA-sequencing

Library preparation, sequencing and analysis were performed according to the SMART-seq2 protocol.[66] Briefly, total RNA was harvested from cells using the RNeasy Mini kit (Qiagen). First, we incubated 20 ng purified total RNA with RNase inhibitor (Clontech Takara, #2313B), dNTP mix (Thermo Fisher Scientific, R0192), and the 3'-RT primer at 72 °C for 3 min to anneal the 3'-RT primer. Next, we performed first-strand synthesis using the template switching oligo (TSO) (Exiqon, Qiagen) together with RNase inhibitor, betaine (Sigma Aldrich B0300-1VL), MgCl2 (Sigma Aldrich, #1028) and Maxima RNase H-minus RT (Thermo Fisher Scientific, #EP0751), according to the manufacturer's protocols. We performed pre-amplification of first-strand libraries with the ISPCR primer KAPA HiFi HotStart (Roche, #KK2601) and SYBR green (Thermo Fisher Scientific). All oligonucleotide sequences are provided in Supplementary Table 27. Whole transcriptome amplification (WTA) product was washed using DNA AMPure XP beads (Beckman Coulter, #A63881) and quantified by Agilent TapeStation. We performed tagmentation and library preparation of 0.25 ng WTA product using the Nextera XT kit (Illumina) and Nextera i7 and Nextera i5 barcoding primers. Samples were pooled and washed using SPRISelect beads (Beckman Coulter, #B23318) and quantified by Agilent TapeStation and the KAPA Universal Library Quantification kit (Roche, #KK4824). Libraries were run on Illumina NextSeq 550 in paired-end mode with 37 forward cycles and 37 reverse cycles and the sequencing data was collected with Illumina Nextseq Control software. The RNA expression data between experimental conditions was compared using Pearson correlation (r).

FASTQs were generated using bcl2fastq v2.20. Raw sequencing data were first processed with Trim Galore v0.6.7 in paired-end mode with default parameters to remove low-quality bases, adapter sequences, and unpaired sequences. Trimmed reads were aligned to the GENCODE mouse reference genome M31 (GRCm39) using STAR (v2.7.10a), quantified using kallisto[67], and refined to canonical coding sequences using CCDS release 21.[68]

For RNA C-to-U off-target analysis, REDItools v1.3 was used to quantify the average frequency of C-to-U editing among all sequenced cytosines in each sample[69], excluding cytosines with read depth

<10 or read quality score <30. The transcriptome-wide C-to-U editing frequency was calculated independently for each biological replicate as: (number of reads in which a cytosine was called as an uracil)/(total number of reads covering all analyzed cytosine).

FXN STR genotype analysis

To analyze *FXN* short tandem-repeat genotypes in 500k UK Biobank samples, we started with the DRAGEN v3.7.8 ExpansionHunter v4 calls provided by the UK Biobank (data field 24062). Using its default parameters, ExpansionHunter accurately estimates allele sizes both shorter and longer than the read length (150 bp). However, for very large expansions that exceed the paired-end sequencing library's fragment length (~455 bp +/- 29 bp in UKBB), ExpansionHunter typically underestimates allele size, reporting it as being approximately equal to the fragment length. This underestimation occurs because ExpansionHunter's depth-based allele size estimation assumes that, for each read pair containing repeats from a given STR locus, at least one mate will map near the STR locus even if the other mate consists entirely of repeat sequences (In-repeat Reads or IRRs). However, for alleles exceeding the fragment length, this assumption breaks down because such alleles produce read pairs where both mates consist entirely of repeat sequence (Paired In-repeat Reads or paired-IRRs)[70]. For most STR loci, the aligner mismaps these paired-IRRs to a small number (<10) of predictable, narrow (<10kb) regions in the reference genome containing long stretches of repeats with the same motif as the STR locus of interest. Based on this observation, ExpansionHunter allows users to optionally specify genomic loci to check for paired-IRRs when genotyping a given STR locus. These additional genomic intervals, termed "off-target regions", enable ExpansionHunter to overcome the limitation described above by counting any paired-IRRs found in these regions. This increases ExpansionHunter's accuracy - particularly its sensitivity - for very large allele sizes. However, since ExpansionHunter cannot determine the true locus of origin for paired-IRRs, the specificity of such estimates can suffer in samples with multiple very large expansions sharing the same repeat motif or in those with elevated levels of highly repetitive reads due to contamination or other technical issues. Therefore, to reduce the chance of false-positives due to off-target regions, we identified samples with two pathogenic-range *FXN* alleles using only ExpansionHunter genotypes without considering off-target regions. Then, for the 31 samples identified in that first pass, we reran ExpansionHunter, using off-target regions to produce more sensitive estimates of the true allele sizes (Supplementary Table 1).

We generated the list of off-target regions by running the wgsim tool (https://github.com/hammer/wgsim) to simulate paired-IRRs (i.e., read pairs where both mates consisted entirely of GAA repeats) with an 0.001 base error rate. These simulated reads were aligned to the GRCh38 reference using the BWA aligner, recording the genomic intervals where the aligner mapped such read pairs. We then set the list of off-target regions to genomic intervals that absorbed

99.5% of these simulated reads, excluding the FXN repeat locus itself. The list of off-target regions was as follows: chr13:102161416-102161881, chr15:38681095-38681583, chr1:101657701-101658187, chr20:22538626-22539223, chr2:220546033-220546610, chr5:127247161-127247640, chr6:50708070-50708556, chr7:20765163-20765529, chr7:37848005-37848522, chr7:84690949-84691442, chrUn_KN707747v1_decoy:1062-2074, chrX:30539802-30540285, chrX:51621350-51621856, chrY:24024122-24024600, chrY:25645531-25646013, chrY:8465308-8465815.

We then ran REViewer v0.2.7 to generate read visualizations for the 31 individuals that had biallelic expansions above the pathogenic threshold. We calculated the Fisher-exact p-value to compare individuals with/without interruptions versus with/without a “Hereditary Ataxia” (G11) ICD-10 code (total = 31).

Oxford Nanopore Technologies sequencing

Initial amplification of the target *FXN* GAA locus was performed using an amplification method described above. Amplicons were purified using the QIAquick PCR purification kit (Qiagen, #28104), for further use in library preparation and sequencing. Around 400 ng of purified PCR amplicons were used as input for library preparation. The library was prepared following the Oxford Nanopore Technologies (ONT) native barcoding protocol using SQK-NBD114.24 kit, in accordance with the manufacturer’s instructions. The DNA amplicons were end-repaired and dA-tailed using NEBNext FFPE DNA Repair Mix (New England Biolabs, #M6630) and NEBNext End repair/dA-tailing (New England Biolabs, #E7546). Native barcodes were ligated to the end-repaired DNA using NEB Blunt/TA Ligase Master Mix (New England Biolabs, #M0367). Adaptor ligation to the pooled barcoded library was performed using NEBNext quick ligation module (New England Biolabs, #E6056). The final library was purified with AMPure Beads in 0.4× ratio of the reaction volume and ONT short fragment buffer (SFB-SQK-LSK109). Around 20 ng (~15 fmol) of the purified final libraries were loaded onto an ONT-MinION flow cell (MinION R10.4.1) and sequenced on ONT-MinION MK1B device for a duration of around 24 h.

The Guppy basecaller (v.3.5.2) was used to convert raw fast5 files into pooled fastq files using ONT-provided parameters for DNA running at 400 bp/s with a data recording rate of 5 kHz (r10.4.1 e8.2). After demultiplexing, the fastq files were mapped to the reference human *FXN* gene sequence and analyzed with powTNRka, as described earlier.

References

1. Arbab, M., Shen, M. W., *et al.* Determinants of Base Editing Outcomes from Target Library Analysis and Machine Learning. *Cell* **182**, 463-480.e30 (2020).

2. Komor, A. C., Kim, Y. B., *et al.* Programmable editing of a target base in genomic DNA without double-stranded DNA cleavage. *Nature* **533**, 420–424 (2016).

3. Gaudelli, N. M., Komor, A. C., *et al.* Programmable base editing of A•T to G•C in genomic DNA without DNA cleavage. *Nature* **551**, 464–471 (2017).

4. Koblan, L. W., Doman, J. L., *et al.* Improving cytidine and adenine base editors by expression optimization and ancestral reconstruction. *Nat Biotechnol* **36**, 843–846 (2018).

5. Fu, Y., Foden, J. A., *et al.* High-frequency off-target mutagenesis induced by CRISPR-Cas nucleases in human cells. *Nature biotechnology* **31**, 822–826 (2013).

6. Hsu, P. D., Scott, D. A., *et al.* DNA targeting specificity of RNA-guided Cas9 nucleases. *Nature Biotechnology 2013 31:9* **31**, 827–832 (2013).

7. Anderson, E. M., Haupt, A., *et al.* Systematic analysis of CRISPR-Cas9 mismatch tolerance reveals low levels of off-target activity. *Journal of biotechnology* **211**, 56–65 (2015).

8. Doman, J. L., Raguram, A., *et al.* Evaluation and minimization of Cas9-independent off-target DNA editing by cytosine base editors. *Nature biotechnology* **38**, 620–628 (2020).

9. Cancellieri, S., Canver, M. C., *et al.* CRISPRitz: rapid, high-throughput and variant-aware in silico off-target site identification for CRISPR genome editing. *Bioinformatics* **36**, 2001–2008 (2020).

10. Lee, J. M., Zhang, J., *et al.* A novel approach to investigate tissue-specific trinucleotide repeat instability. *BMC Systems Biology* **4**, 29 (2010).

11. Lee, J. M., Pinto, R. M., *et al.* Quantification of Age-Dependent Somatic CAG Repeat Instability in Hdh CAG Knock-In Mice Reveals Different Expansion Dynamics in Striatum and Liver. *PLoS ONE* **6**, (2011).

12. Aldous, S. G., Smith, E. J., *et al.* A CAG repeat threshold for therapeutics targeting somatic instability in Huntington's disease. *Brain* (2024) doi:10.1093/BRAIN/AWAE063.

13. Belgrad, J. & Khvorova, A. More than 185 CAG repeats: a point of no return in Huntington's disease biology. *Brain* (2024) doi:10.1093/BRAIN/AWAE105.

14. Saffert, P., Adamla, F., *et al.* An Expanded CAG Repeat in Huntingtin Causes +1 Frameshifting. *The Journal of Biological Chemistry* **291**, 18505 (2016).

15. Girstmair, H., Saffert, P., *et al.* Depletion of Cognate Charged Transfer RNA Causes Translational Frameshifting within the Expanded CAG Stretch in Huntingtin. *Cell Reports* **3**, 148–159 (2013).

16. Davies, J. E. & Rubinsztein, D. C. Polyalanine and polyserine frameshift products in Huntington's disease. *Journal of Medical Genetics* **43**, 893 (2006).

17. Sledzinski, P., Nowaczyk, M., *et al.* CRISPR/Cas9-induced double-strand breaks in huntingtin locus lead to CAG repeat contraction through the extensive DNA end resection and homology-mediated repair. doi:10.1101/2023.11.24.568568.

18. Grünewald, J., Zhou, R., *et al.* CRISPR DNA base editors with reduced RNA off-target and self-editing activities. *Nature biotechnology* **37**, 1041–1048 (2019).

19. Zuo, E., Sun, Y., *et al.* Cytosine base editor generates substantial off-target single-nucleotide variants in mouse embryos. *Science* **364**, 289–292 (2019).

20. Sudlow, C., Gallacher, J., *et al.* UK Biobank: An Open Access Resource for Identifying the Causes of a Wide Range of Complex Diseases of Middle and Old Age. *PLOS Medicine* **12**, e1001779 (2015).

21. Dolzhenko, E., Deshpande, V., *et al.* ExpansionHunter: a sequence-graph-based tool to analyze variation in short tandem repeat regions. *Bioinformatics* **35**, 4754–4756 (2019).

22. Depienne, C. & Mandel, J. L. 30 years of repeat expansion disorders: What have we learned and what are the remaining challenges? *American Journal of Human Genetics* **108**, 764 (2021).

23. Dolzhenko, E., Weisburd, B., *et al.* REViewer: haplotype-resolved visualization of read alignments in and around tandem repeats. *Genome Medicine* **14**, 1–10 (2022).

24. Al-Mahdawi, S., Ging, H., *et al.* Large interruptions of GAA repeat expansion mutations in Friedreich ataxia are very rare. *Frontiers in Cellular Neuroscience* (2018) doi:10.3389/fncel.2018.00443.

25. Ohshima, K., Sakamoto, N., *et al.* A nonpathogenic GAAGGA repeat in the Friedreich gene: Implications for pathogenesis. *Neurology* **53**, 1854 (1999).

26. Nethisinghe, S., Kesavan, M., *et al.* Interruptions of the FXN GAA Repeat Tract Delay the Age at Onset of Friedreich's Ataxia in a Location Dependent Manner. *International Journal of Molecular Sciences 2021, Vol. 22, Page 7507* **22**, 7507 (2021).

27. Kawakami, K. Tol2: A versatile gene transfer vector in vertebrates. *Genome Biology* vol. 8 S7 (2007).

28. Arbab, M., Srinivasan, S., *et al.* Cloning-free CRISPR. *Stem Cell Reports* **5**, 908–917 (2015).

29. Tsai, S. Q., Nguyen, N. T., *et al.* CIRCLE-seq: a highly sensitive in vitro screen for genome-wide CRISPR-Cas9 nuclease off-targets. *Nat Methods* **14**, 607–614 (2017).

30. Lazzarotto, C. R., Nguyen, N. T., *et al.* Defining CRISPR–Cas9 genome-wide nuclease activities with CIRCLE-seq. *Nature Protocols* **13**, 2615–2642 (2018).

31. Heinz, S., Benner, C., *et al.* Simple combinations of lineage-determining transcription factors prime cis-regulatory elements required for macrophage and B cell identities. *Molecular cell* **38**, 576 (2010).

32. Clark, R. M., Dalgliesh, G. L., *et al.* Expansion of GAA triplet repeats in the human genome: unique origin of the FRDA mutation at the center of an Alu. *Genomics* **83**, 373–383 (2004).

33. Levy, J. M., Yeh, W. H., *et al.* Cytosine and adenine base editing of the brain, liver, retina, heart and skeletal muscle of mice via adeno-associated viruses. *Nature biomedical engineering* **4**, 97–110 (2020).

34. Foust, K. D., Nurre, E., *et al.* Intravascular AAV9 preferentially targets neonatal neurons and adult astrocytes. *NATURE BIOTECHNOLOGY VOLUME* **27**, (2009).

35. Hammond, S. L., Leek, A. N., *et al.* Cellular selectivity of AAV serotypes for gene delivery in neurons and astrocytes by neonatal intracerebroventricular injection. *PLoS ONE* **12**, (2017).

36. Mathiesen, S. N., Lock, J. L., *et al.* CNS Transduction Benefits of AAV-PHP.eB over AAV9 Are Dependent on Administration Route and Mouse Strain. *Molecular Therapy. Methods & Clinical Development* **19**, 447 (2020).

37. Swiech, L., Heidenreich, M., *et al.* In vivo interrogation of gene function in the mammalian brain using CRISPR-Cas9. *Nature Biotechnology* **33**, 102 (2014).

38. Arbab, M., Matuszek, Z., *et al.* Base editing rescue of spinal muscular atrophy in cells and in mice. *Science (New York, N.Y.)* **380**, eadg6518 (2023).

39. Robbins, K. L., Glascock, J. J., *et al.* Defining the therapeutic window in a severe animal model of spinal muscular atrophy. *Human molecular genetics* **23**, 4559–4568 (2014).

40. Meyer, K., Ferraiuolo, L., *et al.* Improving single injection CSF delivery of AAV9-mediated gene therapy for SMA: a dose-response study in mice and nonhuman primates. *Molecular therapy : the journal of the American Society of Gene Therapy* **23**, 477–487 (2015).

41. Hughes, M. P., Smith, D. A., *et al.* AAV9 intracerebroventricular gene therapy improves lifespan, locomotor function and pathology in a mouse model of Niemann–Pick type C1 disease. *Human Molecular Genetics* **27**, 3079–3098 (2018).

42. Zhang, J., Fakharzadeh, A., *et al.* Atypical structures of GAA/TTC trinucleotide repeats underlying Friedreich's ataxia: DNA triplexes and RNA/DNA hybrids. *Nucleic Acids Research* **48**, 9899–9917 (2020).

43. Wang, Y., Zhao, Y., *et al.* Nanopore sequencing technology, bioinformatics and applications. *Nature Biotechnology 2021 39:11* **39**, 1348–1365 (2021).

44. Delahaye, C. & Nicolas, J. Sequencing DNA with nanopores: Troubles and biases. *PLoS ONE* **16**, (2021).

45. Yaméogo, P., Gérard, C., *et al.* Removal of the GAA repeat in the heart of a Friedreich's ataxia mouse model using CjCas9. *Gene therapy* (2023) doi:10.1038/S41434-023-00387-0.

46. Li, Y., Polak, U., *et al.* Excision of expanded GAA repeats alleviates the molecular phenotype of friedreich's ataxia. *Molecular Therapy* **23**, 1055–1065 (2015).

47. Dabrowska, M., Juzwa, W., *et al.* Precise Excision of the CAG Tract from the Huntingtin Gene by Cas9 Nickases. *Frontiers in Neuroscience* **12**, (2018).

48. Li, J., Rozwadowska, N., *et al.* Excision of the expanded GAA repeats corrects cardiomyopathy phenotypes of iPSC-derived Friedreich's ataxia cardiomyocytes. *Stem cell research* **40**, 101529 (2019).

49. Monteys, A. M., Ebanks, S. A., *et al.* CRISPR/Cas9 Editing of the Mutant Huntingtin Allele In Vitro and In Vivo. *Molecular Therapy* **25**, 12–23 (2017).

50. Ouellet, D. L., Cherif, K., *et al.* Deletion of the GAA repeats from the human frataxin gene using the CRISPR-Cas9 system in YG8R-derived cells and mouse models of Friedreich ataxia. *Gene Therapy* **24**, 265–274 (2017).

51. Yang, S., Li, S. & Li, X.-J. CRISPR/Cas9-mediated gene editing ameliorates neurotoxicity in mouse model of Huntington's disease. *J Clin Invest* **127**, 2719 (2017).

52. Ekman, F. K., Ojala, D. S., *et al.* CRISPR-Cas9-Mediated Genome Editing Increases Lifespan and Improves Motor Deficits in a Huntington's Disease Mouse Model. *Molecular Therapy. Nucleic Acids* **17**, 829 (2019).

53. Anzalone, A. V., Gao, X. D., *et al.* Programmable deletion, replacement, integration and inversion of large DNA sequences with twin prime editing. *Nature biotechnology* **40**, 731–740 (2022).

54. Choi, J., Chen, W., *et al.* Precise genomic deletions using paired prime editing. *Nature Biotechnology 2021 40:2* **40**, 218–226 (2021).

55. Hwang, H. Y., Gim, D., *et al.* Precise editing of pathogenic nucleotide repeat expansions in iPSCs using paired prime editor. *Nucleic Acids Research* **52**, 5792 (2024).

56. Nakamura, M., Gao, Y., *et al.* CRISPR technologies for precise epigenome editing. *Nature Cell Biology 2021 23:1* **23**, 11–22 (2021).

57. Xu, X., Tao, Y., *et al.* A CRISPR-based approach for targeted DNA demethylation. *Cell Discovery 2016 2:1* **2**, 1–12 (2016).

58. Seo, J. H., Shin, J. H., *et al.* DNA double-strand break-free CRISPR interference delays Huntington's disease progression in mice. *Communications Biology 2023 6:1* **6**, 1–12 (2023).

59. Choi, D. E., Shin, J. W., *et al.* Base editing strategies to convert CAG to CAA diminish the disease-causing mutation in Huntington's disease. *eLife* **12**, (2024).

60. Zhang, E., Neugebauer, M. E., *et al.* Phage-assisted evolution of highly active cytosine base editors with enhanced selectivity and minimal sequence context preference. *Nature Communications 2024 15:1* **15**, 1–13 (2024).

61. Neugebauer, M. E., Hsu, A., *et al.* Evolution of an adenine base editor into a small, efficient cytosine base editor with low off-target activity. *Nature Biotechnology 2022 41:5* **41**, 673–685 (2022).

62. Villiger, L., Grisch-Chan, H. M., *et al.* Treatment of a metabolic liver disease by in vivo genome base editing in adult mice. *Nature medicine* **24**, 1519–1525 (2018).

63. Yeh, W. H., Chiang, H., *et al.* In vivo base editing of post-mitotic sensory cells. *Nature Communications* **9**, 1–10 (2018).

64. Davis, J. R., Wang, X., *et al.* Efficient in vivo base editing via single adeno-associated viruses with size-optimized genomes encoding compact adenine base editors. *Nature Biomedical Engineering 2022 6:11* **6**, 1272–1283 (2022).

65. Kuzmin, D. A., Shutova, M. V., *et al.* The clinical landscape for AAV gene therapies. *Nature reviews. Drug discovery* **20**, 173–174 (2021).

66. Picelli, S., Faridani, O. R., *et al.* Full-length RNA-seq from single cells using Smart-seq2. *Nature Protocols* **9**, 171–181 (2014).

67. Bray, N. L., Pimentel, H., *et al.* Near-optimal probabilistic RNA-seq quantification. *Nature Biotechnology* **34**, 525–527 (2016).

68. Pujar, S., O'Leary, N. A., *et al.* Consensus coding sequence (CCDS) database: A standardized set of human and mouse protein-coding regions supported by expert curation. *Nucleic Acids Research* **46**, D221–D228 (2018).

69. DIroma, M. A., Ciaccia, L., *et al.* Elucidating the editome: Bioinformatics approaches for RNA editing detection. *Briefings in Bioinformatics* **20**, 436–447 (2019).

70. Dolzhenko, E., Bennett, M. F., *et al.* ExpansionHunter Denovo: A computational method for locating known and novel repeat expansions in short-read sequencing data. *Genome Biology* **21**, 1–14 (2020).

Supplementary Note 1.
UK Biobank genotypes with bi-allelic *FXN* repeat expansions.

We used ExpansionHunter v5 (Dolzhenko et al, Bioinformatics, 35(22): 4754-4756, 2019*)* to genotype the *FXN* locus in 490,381 UK Biobank genomes and identified 31 individuals with bi-allelic *FXN* expansions of ≥ 66 GAA repeats. We then used REViewer (Dolzhenko et al, Genome Medicine*, 14(1):* 1-10, 2022) to generate read visualizations for these 31 genotypes and performed manual review to assess genotype quality and check for interruptions within the repeat sequence.

This document includes all 31 read visualization images and summarizes manual review findings (Table 1, Supplementary Table 1).

ID#	Hereditary Ataxia ICD-10 code (G11)	Genotype quality	Summary of interruptions	Per-allele summary of interruptions	Description of observed interruption patterns	Page
**1**		low	pure	pure / pure except 2 GAAA interruptions near right flank	... GAAA + 3 x GAA + GAAA + 18 x GAA	*25*
**2**	Yes	low	pure	pure / pure except 1 GAAA interruption near right flank	... GAAA + 4 x GAA	*25*
**3**		low	pure	pure / pure		*25*
**4**		medium	interrupted	pure / interrupted	scattered GAG repeats in ~1/2 the reads	*26*
**5**	Yes	medium	pure	pure / pure except a few scattered GAAA interruptions	a few scattered GAAA interruptions in the middle	*26*
**6**		high	interrupted	pure / interrupted	16 x GAA + 9 x GAAGAG + ~52 x GAA ? + 9 x GAAGAG + GAAA + 5 x GAA	*26*
**7**	Yes	medium	pure	pure / pure		*27*
**8**		high	interrupted	pure / interrupted	... GAAA + 17 x GAA. Scattered GAG repeats in ~2/3 of the reads	*27*
**9**		medium	interrupted	interrupted / interrupted	scattered GAG interruptions	*27*
**10**		medium	interrupted	interrupted / interrupted	scattered GAG interruptions	*28*
**11**		low	interrupted	pure / interrupted	11 x GAA + 7 x GAAGAG + .. + GAAA + 19 x GAA	*28*
**12**		low	interrupted	interrupted / interrupted	20 x GAA + .. GAA, GAAGAG .. + 12 x GAA	*28*
**13**		medium	interrupted	interrupted / interrupted	9 x GAA + >9 x GAAGAG + 6 x GAA + .. GAAA + 19 x GAA	*29*
**14**		medium	pure	pure / pure		*29*
**15**		high	interrupted	pure / interrupted	30 x GAA + >6 x GAAGAG .. GAAA + 5 x GAA	*29*
**16**		high	interrupted	pure / interrupted	13 x GAA + >15 x GAAGAG .. GAAA + 17 x GAA	*30*
**17**	Yes	high	pure	pure / pure	... GAAA + 3 x GAA	*30*
**18**		medium	interrupted	interrupted / interrupted	10 x GAA + >14 x GAAGAG .. GAAA + 17 x GAA	*30*
**19**		medium	interrupted	pure / interrupted?	20 x GAA + >17 x GAG	*31*
**20**		medium	interrupted	pure / interrupted	13 x GAA + >11 x GAAGAG ..	*31*
**21**		low	pure	pure / pure		*32*
**22**		low	interrupted	pure / interrupted	... ~ 20 x GAG ..	*32*
**23**		medium	pure	pure / pure		*33*
**24**		medium	interrupted	pure / interrupted	... ~ 38 x GAG .. GAAA + 5 x GAA	*33*
**25**		medium	pure	pure / pure		*33*
**26**		high	interrupted	pure / interrupted	.. ~ 25 x GAG + ... + GAAA + 7 x GAA + GAAA + 12 x GAA	*34*
**27**		medium	interrupted	interrupted / interrupted	13 x GAA + >12 x GAAGAG + ... + GAAA + 20 x GAA	*34*
**28**		medium	interrupted	pure / interrupted	11 x GAA + >6 x GAAGAG + ... GAAA + 17 x GAA	*35*
**29**		medium	interrupted	pure / interrupted	9 x GAA + >10 x GAAGAG + ..	*35*
**30**		medium	interrupted	interrupted / interrupted	13 x GAA + >14 x GAAGAG + ... 10 x GAAGAG + 6 x GAA + GAAA + 20 x GAA	*36*
**31**		high	interrupted	interrupted / interrupted	13 x GAA + >14 x GAAGAG + ... GAAA + 17 x GAA or 14 x GAAGAG	*36*

1 of 31: genotype: **135 (pure) / 135 (pure)**, confidence intervals: **68-149 / 90-189**
genotype quality from manual review: **low**
ataxia phenotype: None recorded
current age: **50-60yo**

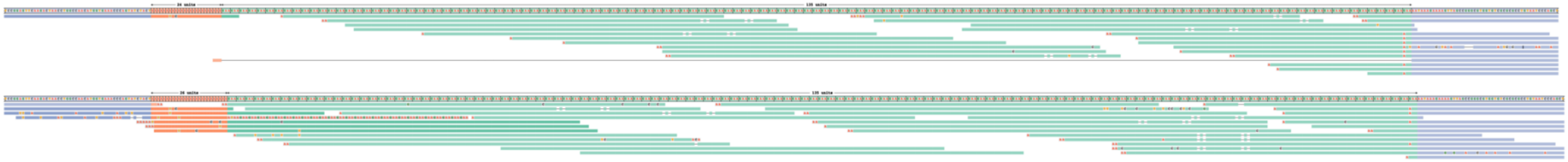

2 of 31: genotype: **129 (pure) / 129 (pure)**, confidence intervals: **58-152 / 85-206**
genotype quality from manual review: **low**
ataxia phenotype: **Hereditary ataxia diagnosis at age 10-20yo**
current age: **50-60yo**

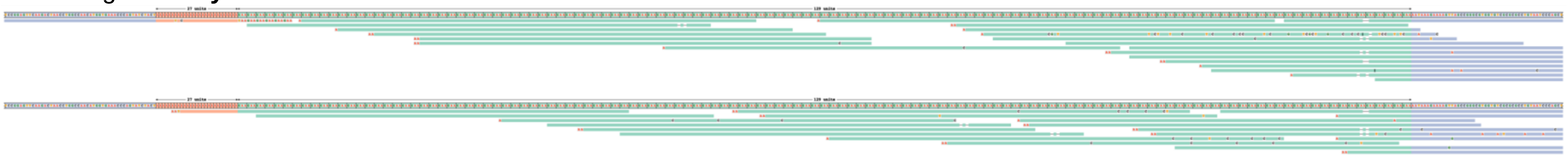

3 of 31: genotype: **127 (pure) / 127 (pure)**, confidence intervals: **73-149 / 95-184**
genotype quality from manual review: **low**
ataxia phenotype: None recorded
current age: **80-90yo**

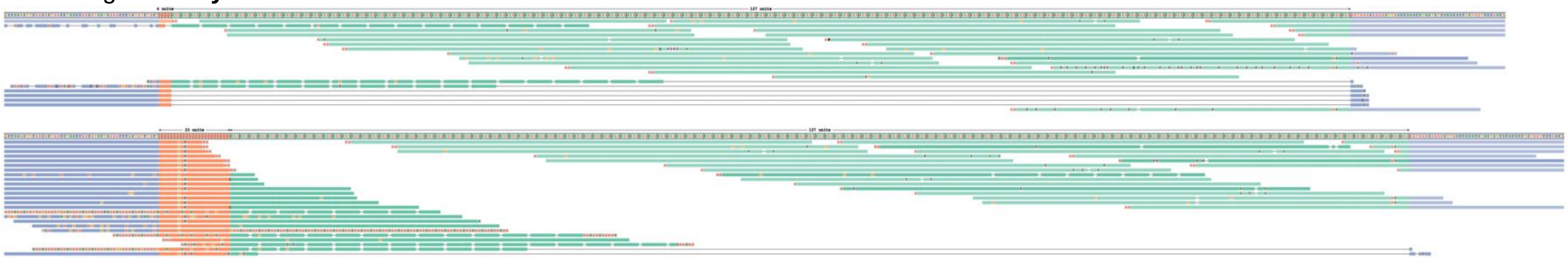

4 of 31: genotype: **125 (pure) / 125 (interrupted)**, confidence intervals: **73-158 / 97-197**
genotype quality from manual review: **medium**
ataxia phenotype: None recorded
current age: **80-90yo**

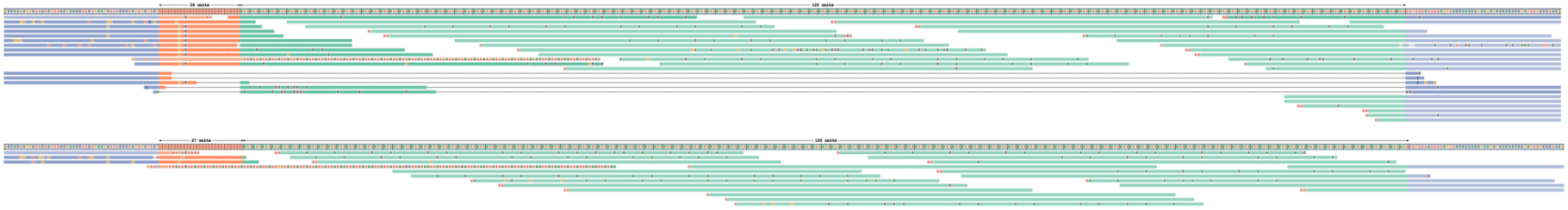

5 of 31: genotype: **114 (pure) / 114 (pure except a few scattered GAAA interruptions)**, confidence intervals: **68-138 / 88-171**
genotype quality from manual review: **medium**
ataxia phenotype: **Hereditary ataxia diagnosis at age 70-80yo**
current age: **80-90yo**

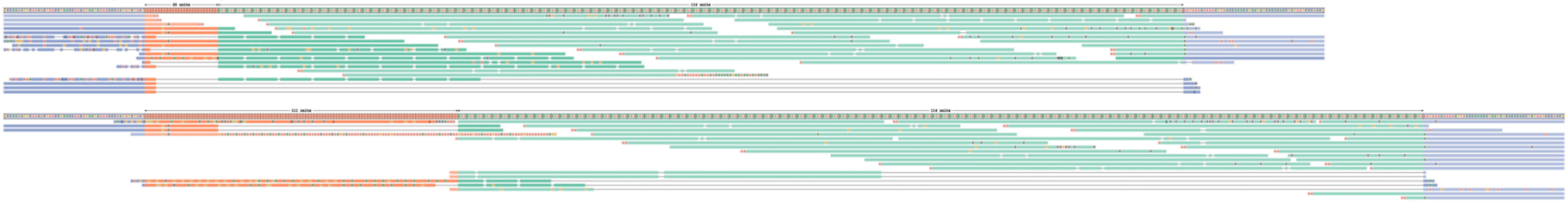

6 of 31: genotype: **112 (pure) / 112 (interrupted)**, confidence intervals: **64-139 / 87-176**
genotype quality from manual review: **medium**
ataxia phenotype: None recorded
current age: **70-80yo**

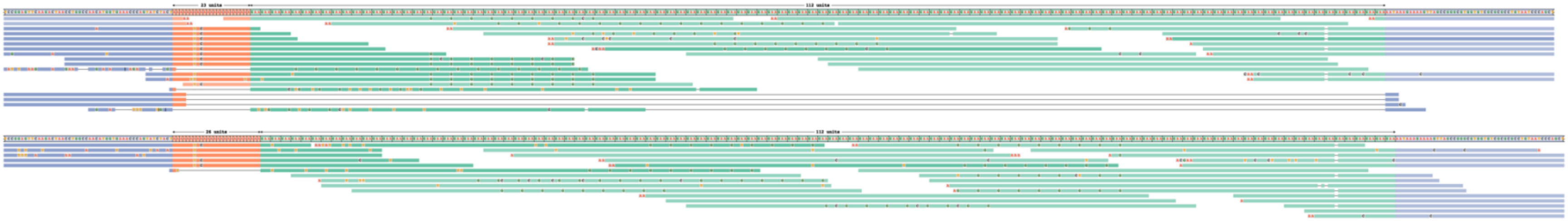

7 of 31: genotype: **109 (pure) / 109 (pure)**, confidence intervals: **53-143 / 81-197**
genotype quality from manual review: **medium**
ataxia phenotype: **Hereditary ataxia diagnosis at age 10-20yo**
age at death: **60-70yo**

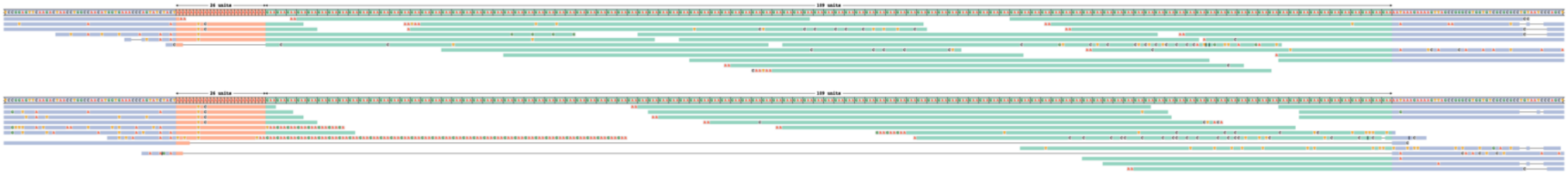

8 of 31: genotype: **109 (pure) / 109 (pure)**, confidence intervals: **62-135 / 85-172**
genotype quality from manual review: **high**
ataxia phenotype: None recorded
current age: **50-60yo**

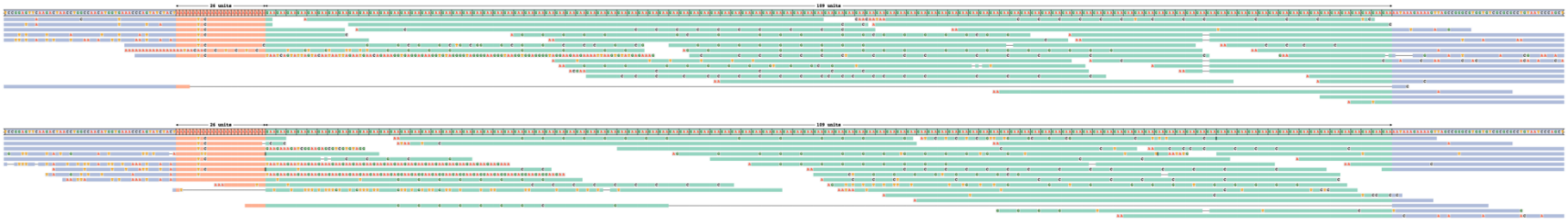

9 of 31: genotype: **105 (interrupted) / 105 (interrupted)**, confidence intervals: **50-134 / 71-201**
genotype quality from manual review: **medium**
ataxia phenotype: None recorded
current age: **60-70yo**

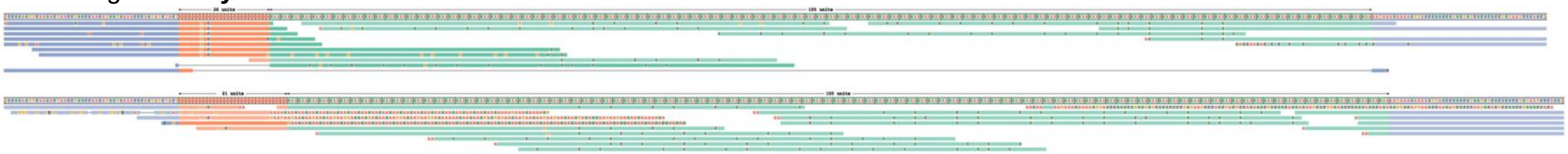

10 of 31: genotype: **103 (interrupted) / 103 (interrupted)**, confidence intervals: **29-145 / 74-208**
genotype quality from manual review: **medium**
ataxia phenotype: None recorded
current age: **80-90yo**

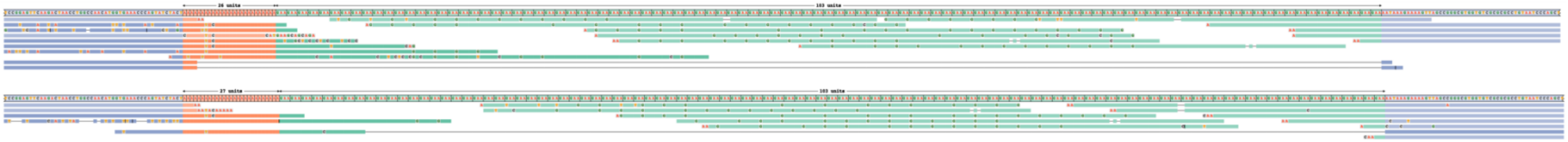

11 of 31: genotype: **102 (pure) / 102 (interrupted)**, confidence intervals: **53-118 / 76-161**
genotype quality from manual review: **low**
ataxia phenotype: None recorded
current age: **60-70yo**

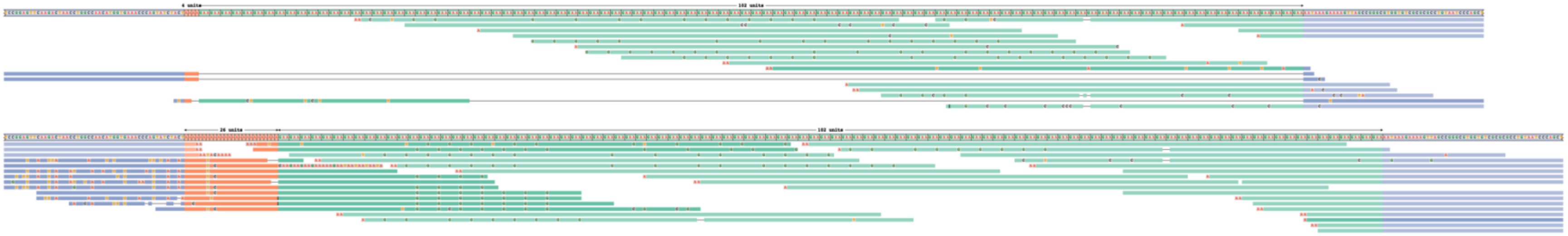

12 of 31: genotype: **100 (interrupted) / 100 (interrupted)**, confidence intervals: **53-138 / 78-193**
genotype quality from manual review: **low**
ataxia phenotype: None recorded
current age: **80-90yo**

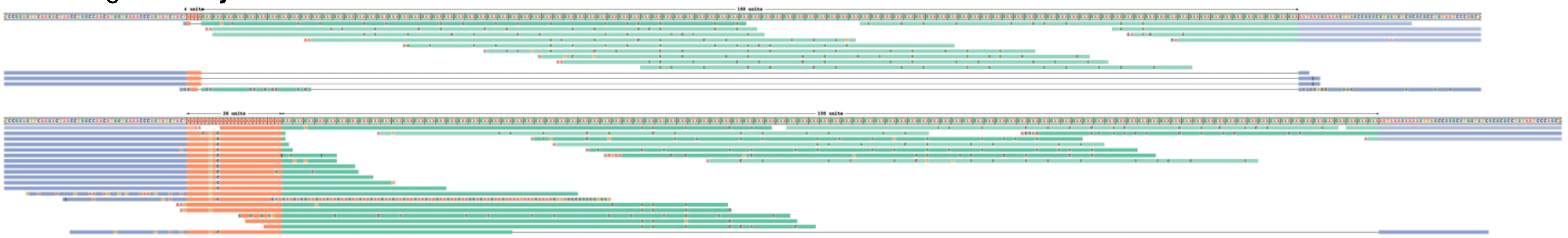

13 of 31: genotype: **100 (interrupted) / 100 (interrupted)**, confidence intervals: **49-146 / 78-207**
genotype quality from manual review: **medium**
ataxia phenotype: None recorded
current age: **80-90yo**

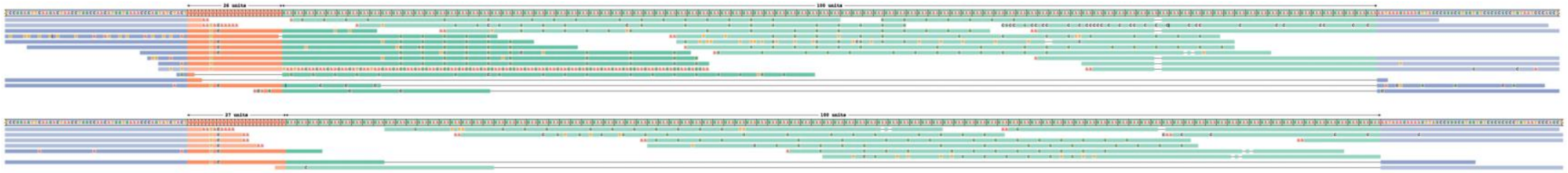

14 of 31: genotype: **98 (pure) / 98 (pure)**, confidence intervals: **43-142 / 69-203**
genotype quality from manual review: **medium**
ataxia phenotype: None recorded
current age: **80-90yo**

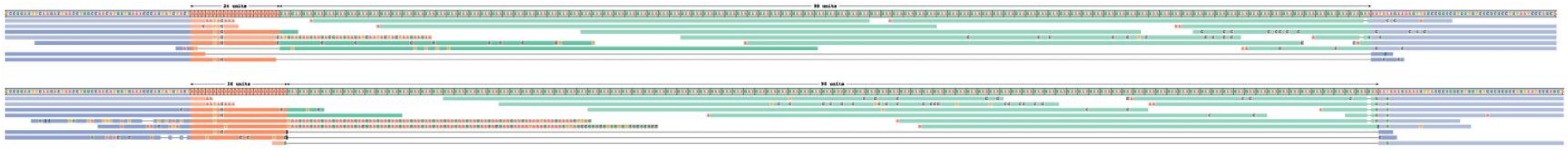

15 of 31: genotype: **98 (pure) / 98 (interrupted)**, confidence intervals: **48-120 / 73-173**
genotype quality from manual review: **high**
ataxia phenotype: None recorded
current age: **70-80yo**

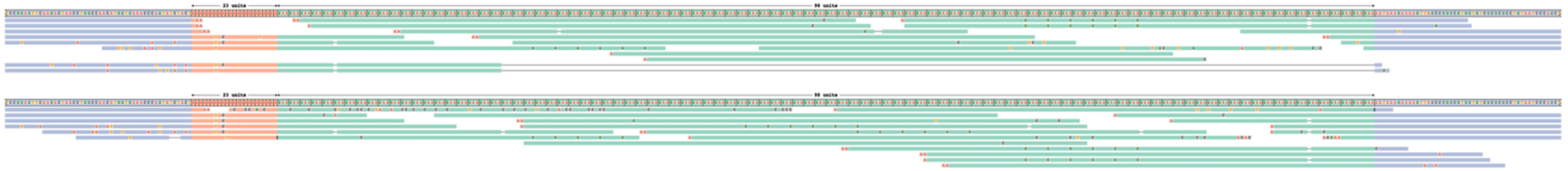

16 of 31: genotype: **96 (pure) / 96 (interrupted)**, confidence intervals: **51-121 / 75-165**
genotype quality from manual review: **high**
ataxia phenotype: None recorded
current age: **80-90yo**

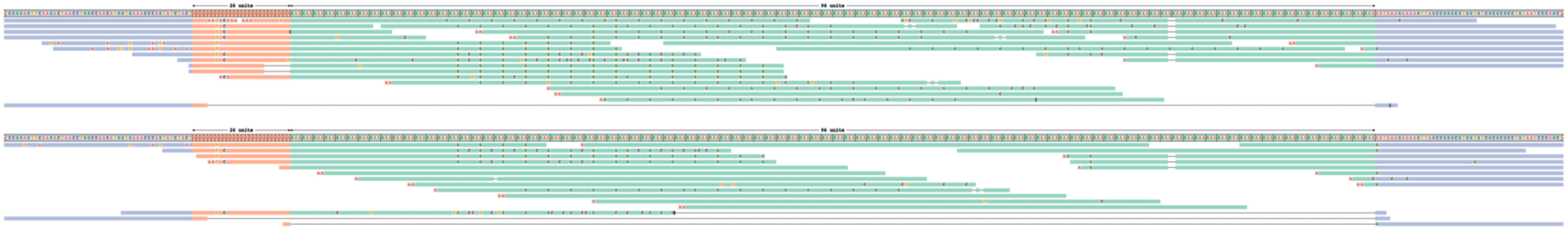

17 of 31: genotype: **95 (pure) / 95 (pure)**, confidence intervals: **50-115 / 73-162**
genotype quality from manual review: **high**
ataxia phenotype: **Hereditary ataxia diagnosis at age 40-50yo**
age at death: **60-70yo**

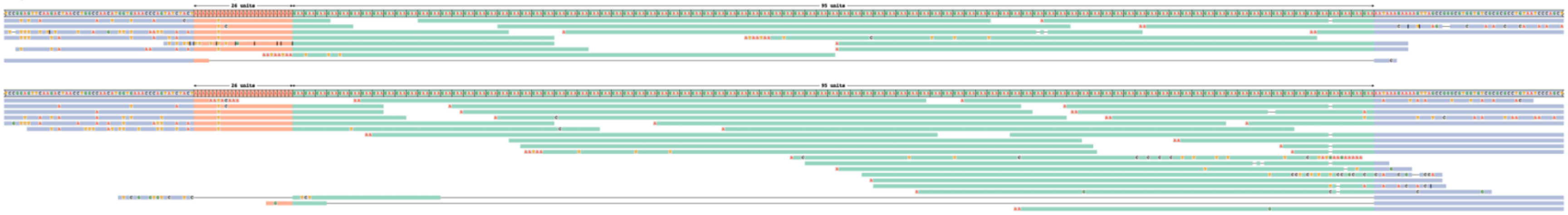

18 of 31: genotype: **90 (interrupted) / 90 (interrupted)**, confidence intervals: **47-127 / 70-195**
genotype quality from manual review: **medium**
ataxia phenotype: None recorded
current age: **80-90yo**

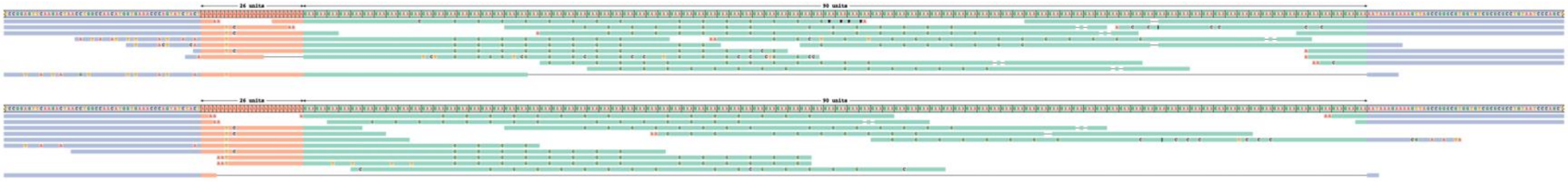

19 of 31: genotype: **90 (pure) / 90 (interrupted)**, confidence intervals: **44-97 / 66-149**
genotype quality from manual review: **medium**
ataxia phenotype: None recorded
age at death: **70-80yo**

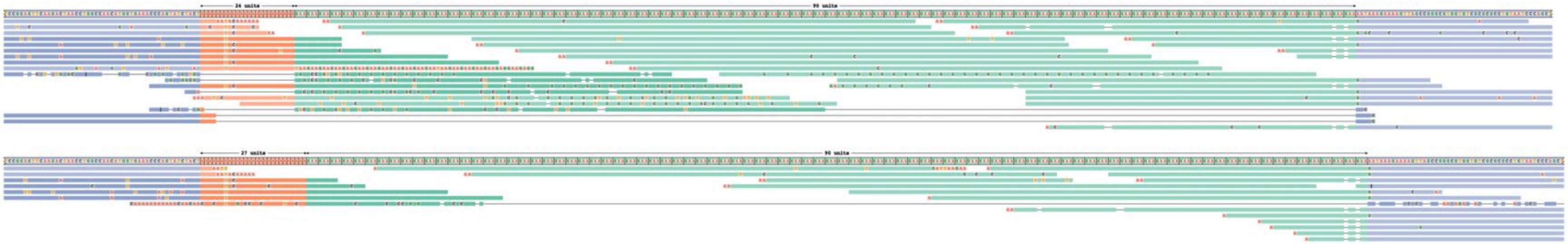

20 of 31: genotype: **89 (pure) / 89 (interrupted)**, confidence intervals: **49-130 / 73-184**
genotype quality from manual review: **medium**
ataxia phenotype: None recorded
current age: **70-80yo**

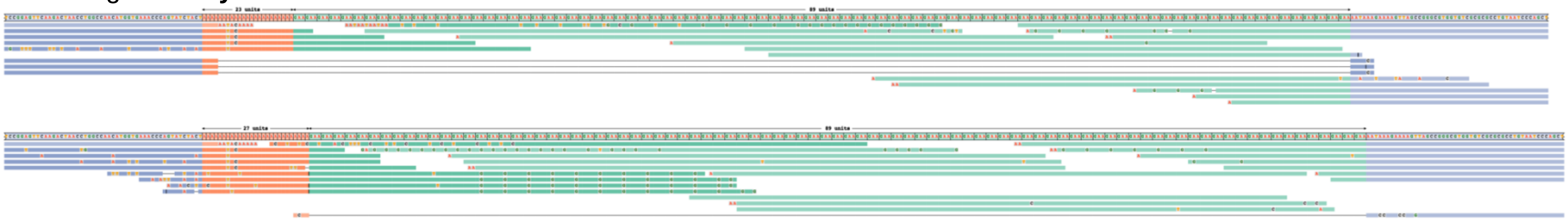

21 of 31: genotype: **89 (pure) / 89 (pure)**, confidence intervals: **50-132 / 73-183**
genotype quality from manual review: **low**
ataxia phenotype: None recorded
current age: **70-80yo**

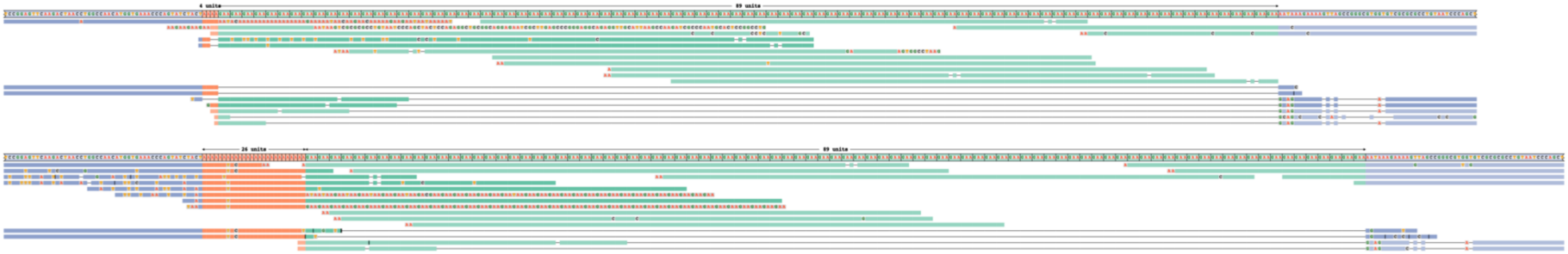

22 of 31: genotype: **85 (pure) / 85 (interrupted)**, confidence intervals: **53-121 / 75-163**
genotype quality from manual review: **low**
ataxia phenotype: None recorded
current age: **80-90yo**

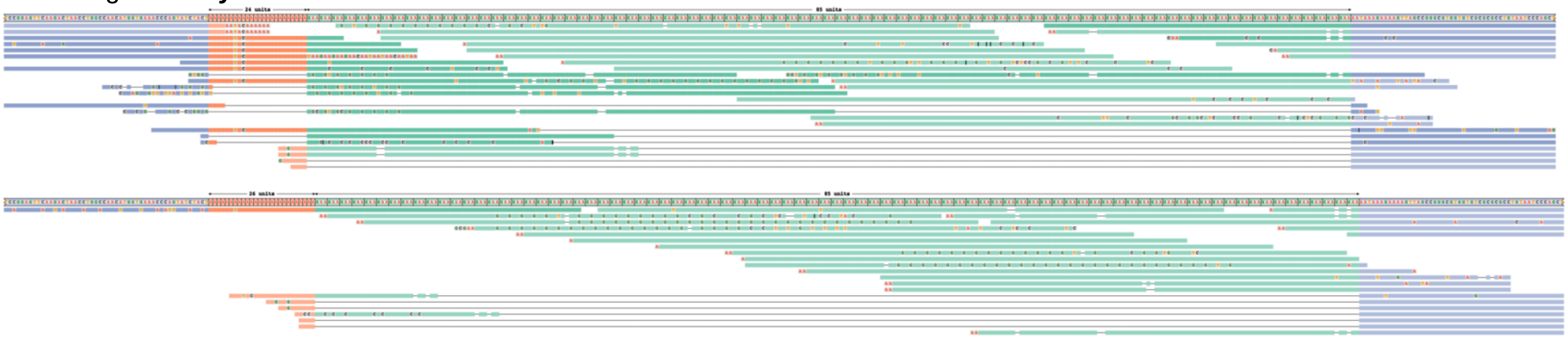

23 of 31: genotype: **85 (pure) / 85 (pure)**, confidence intervals: **44-114 / 66-165**
genotype quality from manual review: **medium**
ataxia phenotype: None recorded
current age: **70-80yo**

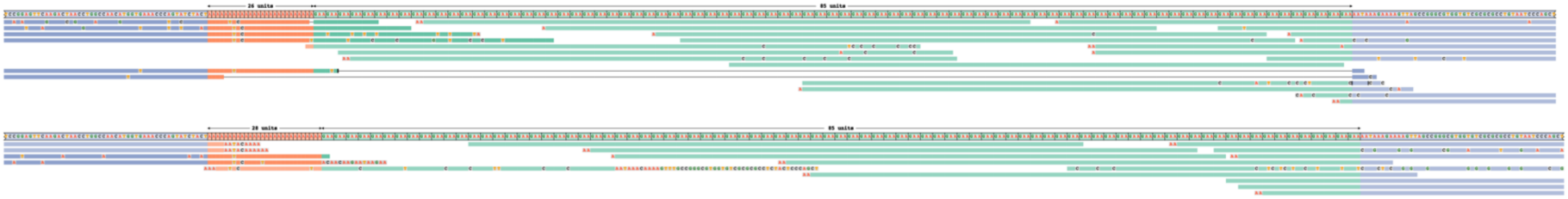

24 of 31: genotype: **84 (pure) / 84 (interrupted)**, confidence intervals: **48-106 / 67-153**
genotype quality from manual review: **low**
ataxia phenotype: None recorded
current age: **70-80yo**

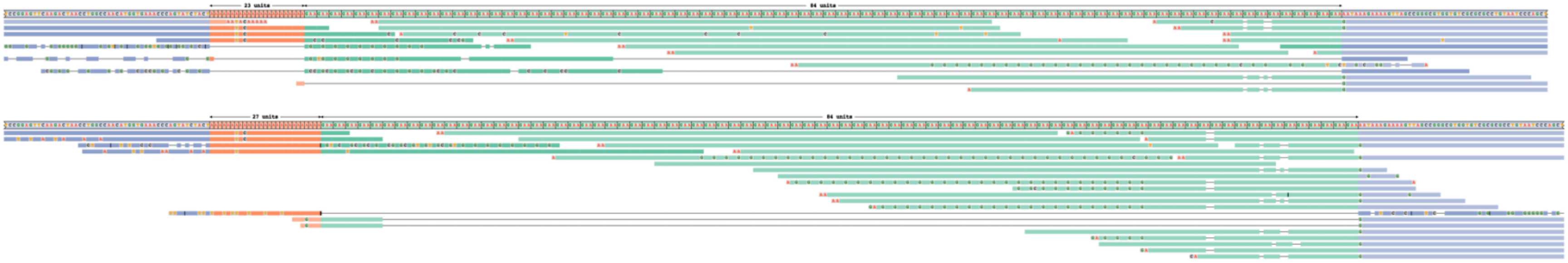

25 of 31: genotype: **83 (pure) / 83 (pure)**, confidence intervals: **38-121 / 63-178**
genotype quality from manual review: **medium**
ataxia phenotype: None recorded
current age: **50-60yo**

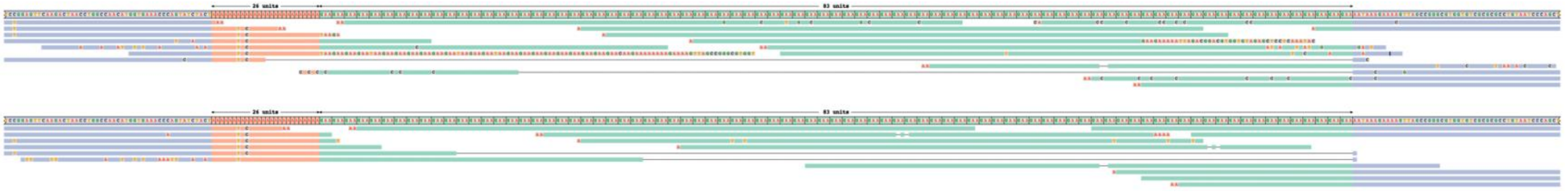

26 of 31: genotype: **82 (pure) / 82 (interrupted)**, confidence intervals: **49-94 / 64-129**
genotype quality from manual review: **high**
ataxia phenotype: None recorded
age at death: **70-80yo**

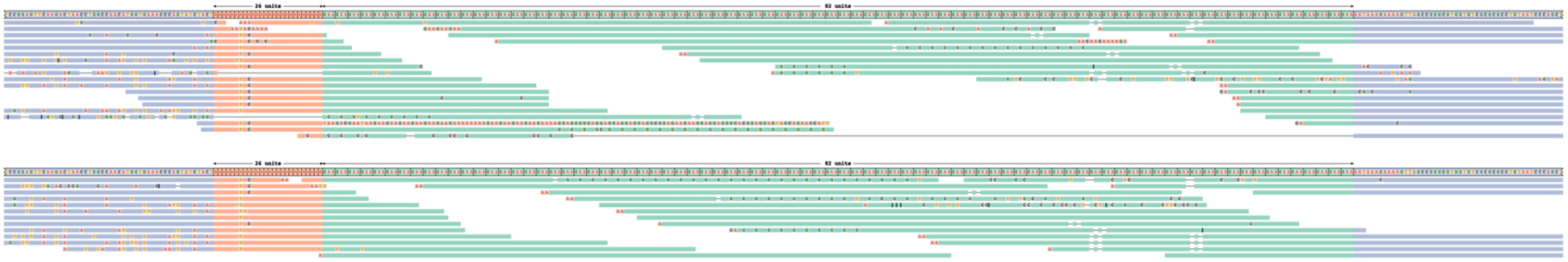

27 of 31: genotype: **82 (interrupted) / 82 (interrupted)**, confidence intervals: **40-124 / 64-210**
genotype quality from manual review: **low**
ataxia phenotype: None recorded
current age: **60-70yo**

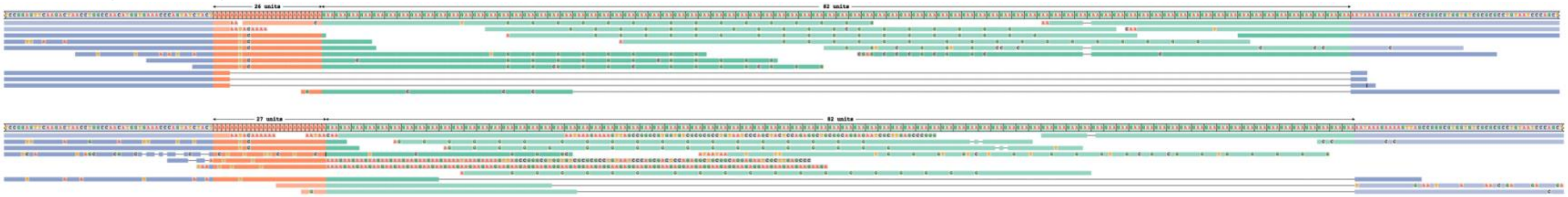

28 of 31: genotype: **82 (pure) / 82 (interrupted)**, confidence intervals: **48-102 / 65-151**
genotype quality from manual review: **medium**
ataxia phenotype: None recorded
current age: **50-60yo**

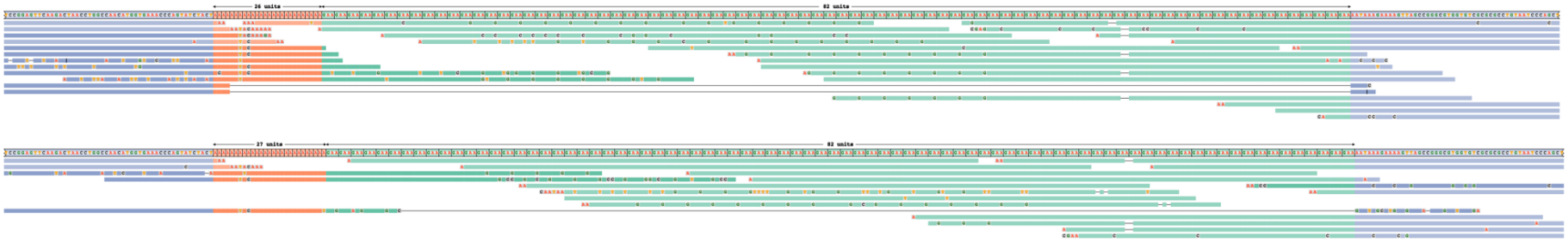

29 of 31: genotype: **79 (pure) / 79 (interrupted)**, confidence intervals: **39-98 / 61-150**
genotype quality from manual review: **medium**
ataxia phenotype: None recorded
current age: **70-80yo**

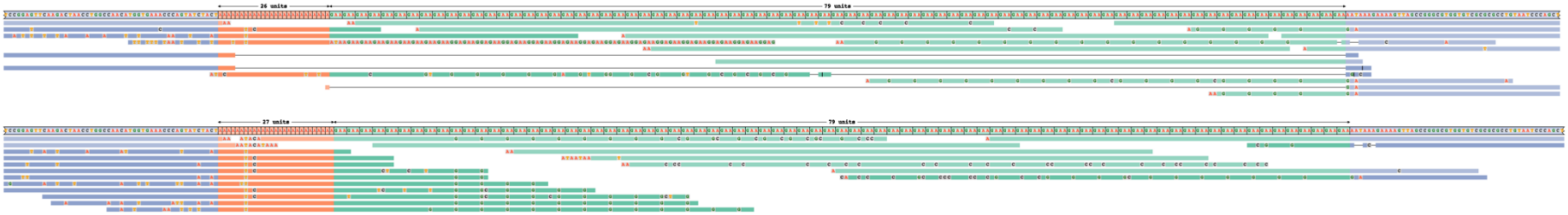

30 of 31: genotype: **79 (interrupted) / 79 (interrupted)**, confidence intervals: **49-103 / 67-165**
genotype quality from manual review: **medium**
ataxia phenotype: None recorded
current age: **60-70yo**

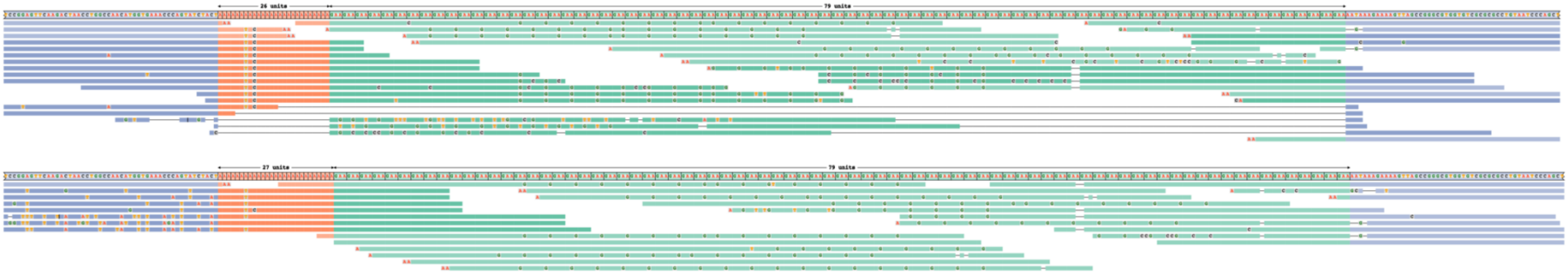

31 of 31: genotype: **66 (interrupted) / 66 (interrupted)**, confidence intervals: **45-84 / 59-114**
genotype quality from manual review: **high**
ataxia phenotype: None recorded
current age: **80-90yo**

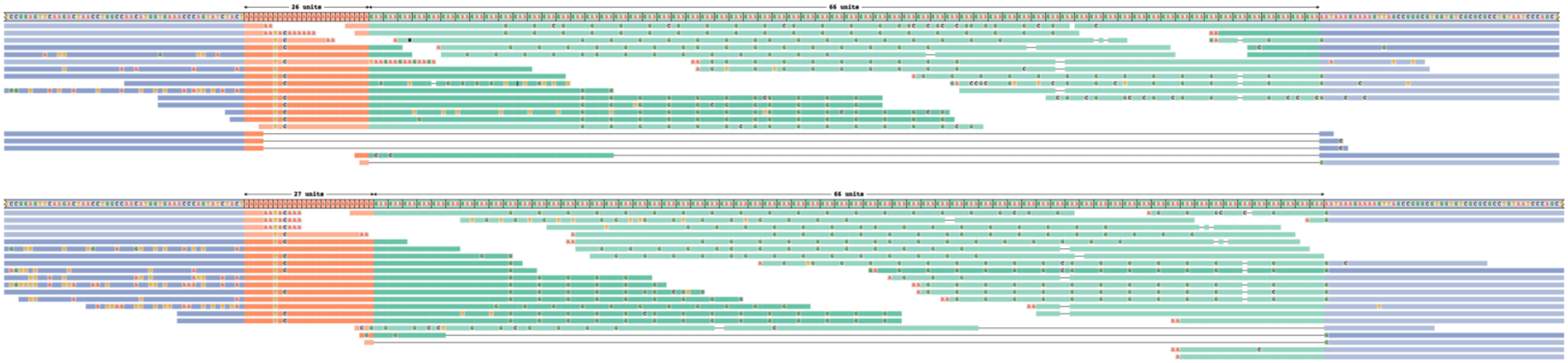

Supplementary Note 2.

Explanation and the algorithm for *powTNRka* analysis software tool.

Most pairwise sequence alignment algorithms find an optimal alignment of two strings (in this case, a reference sequence $\mathcal{R}$ and a sequenced read $\mathcal{Q}$) by maximizing a score made of rewards for matched characters and penalties for mismatched characters or gaps. This score is typically computed with dynamic programming by filling in a scoring matrix M of shape $[\mathcal{R}.length + 1, \mathcal{Q}.length + 1]$ such that each entry $M_{i,j}$ represents the optimal score for an alignment of subsequences $\mathcal{R}[1..i]$ and $\mathcal{Q}[1..j]$. Letting m_{ij} be the match/mismatch score between $\mathcal{R}[i]$ and $\mathcal{Q}[j]$ and g be the gap penalty, this recursion can be written as:

$$M_{i,j} = \max \begin{cases} M_{i-1,j-1} + m_{ij} \\ M_{i,j-1} + g \\ M_{i-1,j} + g \end{cases}$$

Fixed gap penalties are usually undesired in the context of biological sequence alignments because they fail to capture the biological intuition that insertions and deletions (indels) should be "rare" events, and therefore an alignment in which many gaps are clustered together (representing fewer individual indel events) is more biologically plausible than one in which they are not. For example, the following 3 alignments all have identical scores under fixed gap penalty scoring systems, despite Alignments 2 and 3 being more "plausible":

Alignment 1:
```
GTCCTTCC---AGCA---GCA---GCCTCCTC (reference)
||||||||...||||...|||...||||||||
GTCCTTCCAGCAGCAGCAGCAGCAGCCTCCTC (query)
```

Alignment 2:
```
GTCCTTCCAGCAGCAG---------CCTCCTC (reference)
||||||||||||||||.........|||||||
GTCCTTCCAGCAGCAGCAGCAGCAGCCTCCTC (query)
```

Alignment 3:
```
GTCCTTC---------CAGCAGCAGCCTCCTC (reference)
|||||||.........||||||||||||||||
GTCCTTCCAGCAGCAGCAGCAGCAGCCTCCTC (query)
```

PowTNRka alignment:
```
GTCCTTCcagcagcagcagcagcagCCTCCTC (reference)
||||||||||||||||||||||||||||||||
GTCCTTCCAGCAGCAGCAGCAGCAGCCTCCTC (query)
```

Therefore, Gotoh and coworkers introduced the affine gap penalty, in which consecutive gaps are penalized with a gap *extension* score (g_e), rather than the initial gap open score (g_o). With the Gotoh algorithm, Alignments 2 and 3 have higher scores than Alignment 1, and are therefore "optimal".

To implement the Gotoh algorithm, two additional scoring matrices must be added to the recursion to keep track of insertions (I) and deletions (D). The recursions for the Gotoh algorithm are:

$$M_{i,j} = \max \begin{cases} M_{i-1,j-1} + m_{ij} \\ I_{i-1,j-1} + m_{ij} \\ D_{i-1,j-1} + m_{ij} \end{cases} \quad I_{i,j} = \max \begin{cases} M_{i,j-1} + g_o \\ I_{i,j-1} + g_e \\ D_{i,j-1} + g_o \end{cases} \quad D_{i,j} = \max \begin{cases} M_{i-1,j} + g_o \\ I_{i-1,j} + g_o \\ D_{i-1,j} + g_e \end{cases}$$

Under the Gotoh algorithm, Alignments 2 and 3 have identical scores. In genome editing experiments with CRISPR-Cas nucleases, we have additional information about where the programmed cut site is within the reference sequence. Therefore, CRISPResso2 further modifies the Gotoh algorithm by adding a gap incentive G_i to the values of I_{ij} and D_{ij}, which is positive at the indices i flanking a cut in $\mathcal{R}$ and 0 elsewhere. CRISPResso2 also disallows alignments with insertions followed immediately by deletions (and vice-versa). These modifications break scoring ties by prioritizing alignments with indels at cutsites. The

CRISPResso2 recursions are:

$$M_{i,j} = \max \begin{cases} M_{i-1,j-1} + m_{ij} \\ I_{i-1,j-1} + m_{ij} \\ D_{i-1,j-1} + m_{ij} \end{cases} \quad I_{i,j} = \max \begin{cases} M_{i,j-1} + g_o + G_i \\ I_{i,j-1} + g_e + G_i \end{cases} \quad D_{i,j} = \max \begin{cases} M_{i-1,j} + g_o + G_i \\ D_{i-1,j} + g_e + G_i \end{cases}$$

While the CRISPResso2 algorithm is excellent for typical genome editing experiments, we found that it was unable to properly align trinucleotide repeats (TNRs), especially in treated samples, because there are many possible "optimal" alignments with identical scores when a reference sequence containing TNRs is provided. Moreover, TNRs are prone to expansion and contraction both *in vivo* throughout the course of the experiment and *in vitro* during sample preparation for targeted amplicon sequencing using PCR.

In addition to preserving the properties of the CRISPResso2 algorithm, an "ideal" alignment algorithm for analyzing the outcomes of our experiments would also have the following properties:

- Aligns sequences with different numbers of repeats to a single reference without calling repeat length variations as indels.
- Calls "true" indels within repeats as indels, even when the overall length is a multiple of the repeat length. For example,
  `GTCCTTCcag-cagcagcagcagcagCCTCCTC` (reference)
  `||||||||||.||||||||||||||.|||||||`
  `GTCCTTCCAGCCAGCAGCAGCAGCA-CCTCCTC` (query)
  is called as an indel despite the repetitive sequence having a `cag` length that is a multiple of 3.
- Users should be able to specify programmed base edits. For example, for cytosine base editing of CAG repeats in the example, programmed C•G-to-T•A transitions should not affect the alignment score.
  `GTCCTTCyaryaryaryaryaryarCCTCCTC` (reference; Y = C or T; R = A or G)
  `||||||||||||||||||||||||||||||||`
  `GTCCTTCCAACAACAGTAACAATAGCCTCCTC` (query)
- For very long amplicons, should gracefully handle reads that end in the middle of repeats:
  `GTCCTTCcagcagcagcagcagcagcagcagcagcagcagca` (reference)
  `||||||||||||||||||||||||||||||||||||||||||`
  `GTCCTTCCAGCAGCAGCAGCAGCAGCAGCAGCAGCAGCAGCA` (query)

We developed powTNRka, an alignment algorithm that meets all of the above desiderata. powTNRka allows users to specify templates containing defined repeats that are analyzed for the presence of substitutions, insertions, and deletions.

As input, powTNRka takes a template containing r repeat sequences $\mathcal{S}_1 ... \mathcal{S}_r$ with lengths $\ell_1 ... \ell_r$ at indices I_r. To call repeats, powTNRka constructs r additional scoring matrices $R^{(1)} ... R^{(r)}$ where $R^{(k)}$ has shape $[\mathcal{S}_k.length, \mathcal{Q}.len + 1]$.

The powTNRka recursions are as follows:

$$M_{i,j} = \begin{cases} -\infty & \text{if } i = I_r[k] & \text{(In repeat)} \\ \max \begin{cases} R^{(k)}_{\ell_k,j-1} + m_{ij} \\ \max\limits_{1\le n\le \ell_k-1} R^{(k)}_{n,j-1} + g_o + m_{ij} \end{cases} & \text{if } (i-1) = I_r[k] & \text{(After repeat)} \\ \max \begin{cases} M_{i-1,j-1} + m_{ij} \\ I_{i-1,j-1} + m_{ij} \\ D_{i-1,j-1} + m_{ij} \end{cases} & \text{elsewise} & \text{(Normal Gotoh recursion)} \end{cases} \tag{1}$$

$$I_{i,j} = \begin{cases} -\infty & \text{if } i \in I_r & \text{(In repeat)} \\ \max \begin{cases} M_{i,j-1} + g_o + G_i \\ I_{i,j-1} + g_e \end{cases} & \text{elsewise} & \text{(CRISPResso2 recursion)} \end{cases} \tag{2}$$

$$D_{i,j} = \begin{cases} -\infty & \text{if } i \in I_r & \text{(In repeat)} \\ \max \begin{cases} M_{i-1,j} + g_o + G_i \\ D_{i-1,j} + g_e \end{cases} & \text{elsewise} & \text{(CRISPResso2 recursion)} \end{cases} \tag{3}$$

$$R^{(k)}_{n,j} = \begin{cases} \max \begin{cases} M_{I_r[k]-1,j-1} + m^{(k)}_{1j} \\ R^{(k)}_{\ell_k,j-1} + m^{(k)}_{1j} \\ R^{(k)}_{1,j-1} + g_o \\ R^{(k)}_{\ell_k,j} + g_o \end{cases} & \text{if } n = 1 & \text{(First repeat base)} \\ \max \begin{cases} R^{(k)}_{n-1,j-1} + m^{(k)}_{nj} \\ R^{(k)}_{n,j-1} + g_o \\ R^{(k)}_{n-1,j} + g_o \end{cases} & \text{elsewise} & \text{(Other repeat bases)} \end{cases} \tag{4}$$

The directions used in the traceback matrix $\mathcal{T}$ are:

$$\mathcal{T}_{i,j} = \begin{cases} \begin{cases} \text{``}\nwarrow\text{''} & \text{if } M_{ij} = \max(M_{ij}, I_{ij}, D_{ij}) \\ \text{``}\leftarrow\text{''} & \text{if } I_{ij} = \max(M_{ij}, I_{ij}, D_{ij}) \\ \text{``}\uparrow\text{''} & \text{if } D_{ij} = \max(M_{ij}, I_{ij}, D_{ij}) \end{cases} & \text{if } i \notin (I_r \cup I_h) & \text{(Normal Gotoh recursion)} \\ \begin{cases} \text{``}\nwarrow\text{''} & \text{if } R^{(k)}_{1,j} = M_{I_r[k]-1,j-1} + m^{(k)}_{1j} \\ \text{``}\leftarrow\text{''} & \text{elsewise} \end{cases} & \text{if } i = I_r[k] & \text{(In repeat)} \end{cases} \tag{5}$$

Algorithm 1: The powTNRka algorithm

Data: reference $\mathcal{R}$, query $\mathcal{Q}$, repeats $\mathcal{S}_1 ... \mathcal{S}_r$, repeat indices I_r
Result: A_r and A_q, an optimal alignment of $\mathcal{R}$ and $\mathcal{Q}$, respectively

```
/* Store lengths as separate variables for convenience */
```
$\mathcal{L}_r \leftarrow \mathcal{R}.length$; $\mathcal{L}_q \leftarrow \mathcal{Q}.length$
```
/* Initialize dynamic programming tables */
```
let M, I, D, and $\mathcal{T}$ be new tables with indices $[0..\mathcal{L}_r, 0..\mathcal{L}_q]$
let R be a length-r array of references to tables
for $k = 1..r$ **do**
  $\ell_k \leftarrow \mathcal{S}_k.length$
  let $R^{(k)}$ be a new table with indices $[1..\ell_k, 0..\mathcal{L}_q]$
  for $n = 1..\ell_k$ **do**
    $R^{(k)}_{n,0} \leftarrow -\infty$
```
/* Fill in row/column 0 of each table with appropriate values */
```
$M_{0,0} \leftarrow 0$; $I_{0,0} \leftarrow -\infty$; $D_{0,0} \leftarrow -\infty$; $\mathcal{T}_{0,0} \leftarrow \emptyset$
for $i = 1..\mathcal{L}_r$ **do**
  $M_{i,0} \leftarrow -\infty$; $I_{i,0} \leftarrow -\infty$; $D_{i,0} \leftarrow i \cdot g_e$; $\mathcal{T}_{i,0} \leftarrow$ "$\uparrow$"
for $j = 1..\mathcal{L}_q$ **do**
  $M_{0,j} \leftarrow -\infty$; $I_{0,j} \leftarrow j \cdot g_e$; $D_{0,j} \leftarrow -\infty$; $\mathcal{T}_{0,j} \leftarrow$ "$\leftarrow$"
```
/* Fill in dynamic programming tables according to equations 1-5 */
```
for $i = 1..\mathcal{L}_r$ **do**
  for $j = 1..\mathcal{L}_q$ **do**
    if $i \notin I_r$ **then**
      fill in $M_{i,j}$, $I_{i,j}$, and $D_{i,j}$ using Eqs. (1), (2), and (3) `// Non-repeat scoring`
    else
      let k be the index of the repeat such that $i = I_r[k]$
      $\ell_k \leftarrow \mathcal{S}_k.length$
      for $n = 1..\ell_k$ **do**
        fill in $R^{(k)}_{n,j}$ using Eq. (4) `// Repeat scoring`
    fill in $\mathcal{T}_{i,j}$ using Eq. (5) `// Fill in traceback matrix with appropriate direction`
$i \leftarrow \arg\max_{1 \le i \le \mathcal{L}_r} M_{i,\mathcal{L}_q}$ `// Set i to be the highest scoring position in the reference`
$j \leftarrow \mathcal{L}_q$
$d \leftarrow \mathcal{T}_{i,j}$
$A_r \leftarrow \langle\rangle$; $A_q \leftarrow \langle\rangle$ `// Aligned reference/query sequences`
```
/* Follow the directions in the traceback matrix until the upper-left corner is reached */
```
while $d \neq \emptyset$ **do**
  if $d =$ "$\nwarrow$" **then**
    add $\mathcal{R}[i]$ to the left of A_r
    add $\mathcal{Q}[j]$ to the left of A_q
    $i \leftarrow i - 1$
    $j \leftarrow j - 1$
  else if $d =$ "$\leftarrow$" **then**
    if $i = I_r[k]$ **then**
      add `k` to the left of A_r
    else
      add `-` to the left of A_r
    add $\mathcal{Q}[j]$ to the left of A_q
    $j \leftarrow j - 1$
  else
    add $\mathcal{R}[i]$ to the left of A_r
    add `-` to the left of A_q
    $i \leftarrow i - 1$
return A_r, A_q
