# Supplementary material for: Base editing of trinucleotide repeats that cause Huntington’s disease and Friedreich’s ataxia reduces somatic repeat expansions in patient cells and in mice
Source: Nat Genet. 2025 May 26;57(6):1437–51. doi: 10.1038/s41588-025-02172-8 (PMC12165863; doi:10.1038/s41588-025-02172-8)
Supplement: Supplementary file 2 — Reporting Summary [file 41588_2025_2172_MOESM2_ESM.pdf]

natureresearch

Corresponding author(s): David R. Liu

Last updated by author(s): 7/01/2025

Reporting Summary

Nature Research wishes to improve the reproducibility of the work that we publish. This form provides structure for consistency and transparency in reporting. For further information on Nature Research policies, see Authors & Referees and the Editorial Policy Checklist.

Statistics

For all statistical analyses, confirm that the following items are present in the figure legend, table legend, main text, or Methods section.

n/a	Confirmed	
☐	☒	The exact sample size (*n*) for each experimental group/condition, given as a discrete number and unit of measurement
☐	☒	A statement on whether measurements were taken from distinct samples or whether the same sample was measured repeatedly
☐	☒	The statistical test(s) used AND whether they are one- or two-sided<br>*Only common tests should be described solely by name; describe more complex techniques in the Methods section.*
☐	☒	A description of all covariates tested
☐	☒	A description of any assumptions or corrections, such as tests of normality and adjustment for multiple comparisons
☐	☒	A full description of the statistical parameters including central tendency (e.g. means) or other basic estimates (e.g. regression coefficient) AND variation (e.g. standard deviation) or associated estimates of uncertainty (e.g. confidence intervals)
☒	☐	For null hypothesis testing, the test statistic (e.g. *F*, *t*, *r*) with confidence intervals, effect sizes, degrees of freedom and *P* value noted<br>*Give P values as exact values whenever suitable.*
☒	☐	For Bayesian analysis, information on the choice of priors and Markov chain Monte Carlo settings
☒	☐	For hierarchical and complex designs, identification of the appropriate level for tests and full reporting of outcomes
☐	☒	Estimates of effect sizes (e.g. Cohen's *d*, Pearson's *r*), indicating how they were calculated

Our web collection on statistics for biologists contains articles on many of the points above.

Software and code

Policy information about availability of computer code

Data collection	Illumina Miseq Control software (v3.1) was used on the Illumina Miseq sequencers to collect the high-throughput DNA sequencing data. ddPCR data was obsained with QX ONE Droplet Digital PCR system (BioRad). Long-read sequencing data were obtained with an ONT-MinION flow cell (R10.4.1) and sequenced on ONT-MinION MK1B device. NovaSeqX Control software was used to collect WGS data and Illumina Nextseq Control software was used to collect RNA sequencing data.
Data analysis	HTS data was analyzed to quantify editing frequency using custom software powTNRka (v1.0.0) described in Supplementary Note 2 (repeat amplicons) or CRISPResso2 (typical amplicons). ddPCR data was analyzed using OX ONE Software (Bio-Rad). ExpansionHunter (v4) was used to analyze the FXN STR genotypes from UK Biobank data. A list of off-target sites for ExpansionHunter analysis was identified using wgsim tool (https://github.com/hammer/wgsim). Visualizations for the EH data were generated with REViewer v0.2.7. CIRCLE-Seq data was processed using the CIRCLEseq analysis pipeline (https://github.com/tsailabSJ/circleseq) with parameters: "read_threshold: 4; window_size: 3; mapq_threshold: 50; start_threshold: 1; gap_threshold: 3; mismatch_threshold: 6; merged_analysis: True". Genomic region assignment was performed with HOMER. Nanopore sequencing raw fast5 files were converted into pooled fastq files with Guppy basecaller (v.3.5.2) and subsequently analyzed with powTNRka. Whole-genome sequencing data was processed using Illumina DRAGEN software (v3). pysam(v.0.22.1) was used to analyze editing frequency in the WGS data. Off-target predictions for NHP and human genomes was performed using CRISPRitz online prediction tool. FASTQs in the RNAseq experiment were generated using bcl2fastq v2.20, raw sequencing data was processed with Trim Galore v0.6.7, aligned to mouse reference genome using STAR (v2.7.10a), quantified with kallisto and refined to canonical coding sequences with CCDS release 21. REDItools v1.3 was used to quantify the whole-transcriptome C-to-U editing. Long gel images were analyzed with Amersham Typhoon Scanner Control Software 2.0.0.6. HTT.Q111 instability was calculated using GeneMapper vs (Applied Biosystems). YG8s instability was analyzed with Biorad image lab 6.1, R studio v4.2.1 and ImageJ-win64. GraphPad Prism 10 and Microsoft Excel for Mac version 16.86 were used for some analyses.

For manuscripts utilizing custom algorithms or software that are central to the research but not yet described in published literature, software must be made available to editors/reviewers. We strongly encourage code deposition in a community repository (e.g. GitHub). See the Nature Research guidelines for submitting code & software for further information.

Data

Policy information about availability of data

All manuscripts must include a data availability statement. This statement should provide the following information, where applicable:
- Accession codes, unique identifiers, or web links for publicly available datasets
- A list of figures that have associated raw data
- A description of any restrictions on data availability

Illumina high-throughput sequencing data, whole-genome sequencing data, CIRCLE-seq data and RNA-seq data have been deposited in the NCBI Sequence Read Archive database under an accession code PRJNA1193010. Other databases used in this study include: human genome assembly hg19 and hg38, MANE.GRCh38.v1.3.refseq gnomic dataset, UK Biobank data-field 24062, GENCODE mouse reference genome M32 (GRCm39), Human Protein Atlas, Cancer DepMap and AlphaMissense database.

Field-specific reporting

Please select the one below that is the best fit for your research. If you are not sure, read the appropriate sections before making your selection.

☒ Life sciences ☐ Behavioural & social sciences ☐ Ecological, evolutionary & environmental sciences

For a reference copy of the document with all sections, see nature.com/documents/nr-reporting-summary-flat.pdf

Life sciences study design

All studies must disclose on these points even when the disclosure is negative.

Sample size	Sample sizes were determined based on literature precedence and prior experience for genome editing experiments (Gaudelli 2017, Arbab 2023).
Data exclusions	No obtained experimental data were excluded from these analyses.
Replication	All attempts at replication were successful as described. All experiments were performed in at least three biological replicates (studies involving cell lines), unless stated otherwise, or performed on at least three independent animals (in vivo studies).
Randomization	Animals enrolled were randomized and both, male and female animals were used in the study.
Blinding	Blinding was applied where possible. Mice were housed, fed, and handled identically regardless of the treatment. Injections were performed by a researcher distinct from the one responsible for animal handling and downstream analyses. The second experimenter was not provided any specific information about the treatment until data collection was completed. For experiments not involving animals, a single experimenter conducted all procedures, making blinding to treatment conditions impossible.

Reporting for specific materials, systems and methods

We require information from authors about some types of materials, experimental systems and methods used in many studies. Here, indicate whether each material, system or method listed is relevant to your study. If you are not sure if a list item applies to your research, read the appropriate section before selecting a response.

Materials & experimental systems

n/a	Involved in the study	
☒	☐	Antibodies
☐	☒	Eukaryotic cell lines
☒	☐	Palaeontology
☐	☒	Animals and other organisms
☒	☐	Human research participants
☒	☐	Clinical data

Methods

n/a	Involved in the study	
☒	☐	ChIP-seq
☐	☒	Flow cytometry
☒	☐	MRI-based neuroimaging

Eukaryotic cell lines

Policy information about cell lines

Cell line source(s)	129P2/OlaHsd mESC cells were a gift from Richard Sherwood, HEK293T and NIH3T3 cells were obtained from ATCC,fibroblast lines were obtained from Coriell, transgenic mESCs were generated for this study.
Authentication	Cells from ATCC and Coriell were authenticated by the supplier by STR analysis. FXN-mESCs were genotyped by PCR.

Mycoplasma contamination | All cells tested negative for mycoplasma.

Commonly misidentified lines (See ICLAC register) | None used.

Animals and other organisms

Policy information about studies involving animals; ARRIVE guidelines recommended for reporting animal research

Laboratory animals

HTT.Q111 (Htttm5Mem/J): Strain #:003456
YG8s (Tg(FXN)YG8Pook/J): Strain #:030324
C57BL/6J: Strain #:000664

Heterozygous HTT.Q111 males were bred with C57BL/6J females to generate heterozygous litters.
YG8s animals (also known as "Tg(FXN)YG8Pook/J"), carrying FXN human transgene with either 300 or 800 GAA repeats, were generated by breeding males carrying both 300 and 800 YG8s transgene (YG8s300/800) with C57BL/6J wild-type females . All animals were purchased from The Jackson Laboratory. Both male and female animals were used in the study. All injections were performed between PND 0 and 2, and animals were euthanized at 4, 12 or 24 weeks of age.

Wild animals

The study did not involve wild animals.

Field-collected samples

The study did not involve samples collected from the field.

Ethics oversight

Broad's Institutional Biosafety Committee (IBC), the Broad's Institutional Animal Care and Use Committee (IACUC), and relevant IACUC compliance committees at Massachusetts General Hospital provided ethical guidance. Genotyping was performed using standard PCR methods as described by Jax lab or according to previously published protocols. Neonates were intracerebroventricularly injected with split AAV constructs in accordance with IACUC approved protocols. Animals were euthanized at 4, 12 and 24 weeks of age and individual tissues were harvested for genomic DNA analysis or FACS sorting for sequencing analysis and somatic instability analysis. Mice were euthanized according to predetermined criteria, in complience with the Massachusetts General Hospital and OSU IACUC guidelines.

Note that full information on the approval of the study protocol must also be provided in the manuscript.

Flow Cytometry

Plots

Confirm that:

- [ ] The axis labels state the marker and fluorochrome used (e.g. CD4-FITC).
- [ ] The axis scales are clearly visible. Include numbers along axes only for bottom left plot of group (a 'group' is an analysis of identical markers).
- [ ] All plots are contour plots with outliers or pseudocolor plots.
- [ ] A numerical value for number of cells or percentage (with statistics) is provided.

Methodology

Sample preparation

Describe the sample preparation, detailing the biological source of the cells and any tissue processing steps used.

Instrument

Identify the instrument used for data collection, specifying make and model number.

Software

Describe the software used to collect and analyze the flow cytometry data. For custom code that has been deposited into a community repository, provide accession details.

Cell population abundance

Describe the abundance of the relevant cell populations within post-sort fractions, providing details on the purity of the samples and how it was determined.

Gating strategy

Describe the gating strategy used for all relevant experiments, specifying the preliminary FSC/SSC gates of the starting cell population, indicating where boundaries between "positive" and "negative" staining cell populations are defined.

- [ ] Tick this box to confirm that a figure exemplifying the gating strategy is provided in the Supplementary Information.
