# Supplementary figures and images for: Base editing of trinucleotide repeats that cause Huntington’s disease and Friedreich’s ataxia reduces somatic repeat expansions in patient cells and in mice
Source: Nat Genet. 2025 May 26;57(6):1437–51. doi: 10.1038/s41588-025-02172-8 (PMC12165863; doi:10.1038/s41588-025-02172-8)

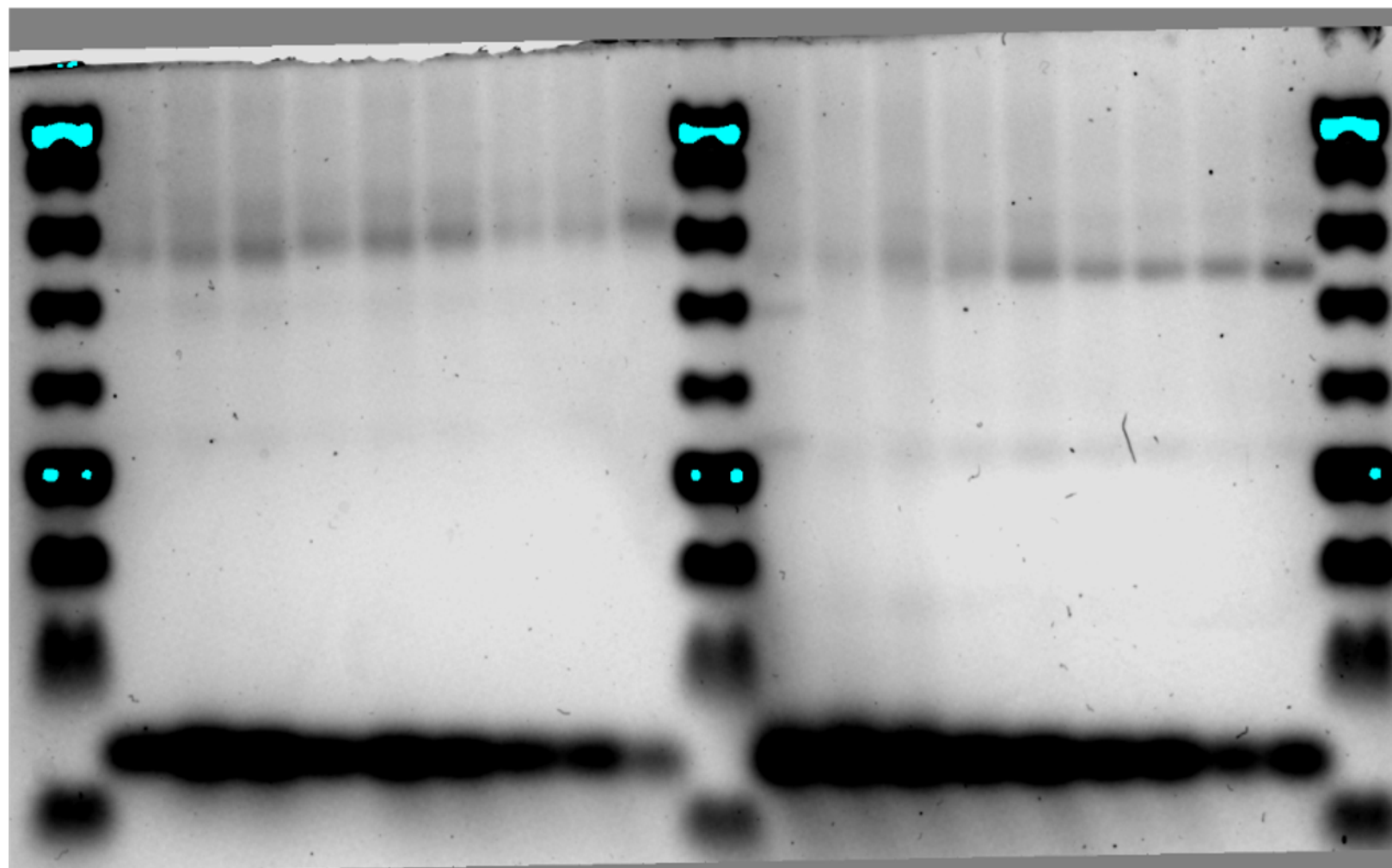

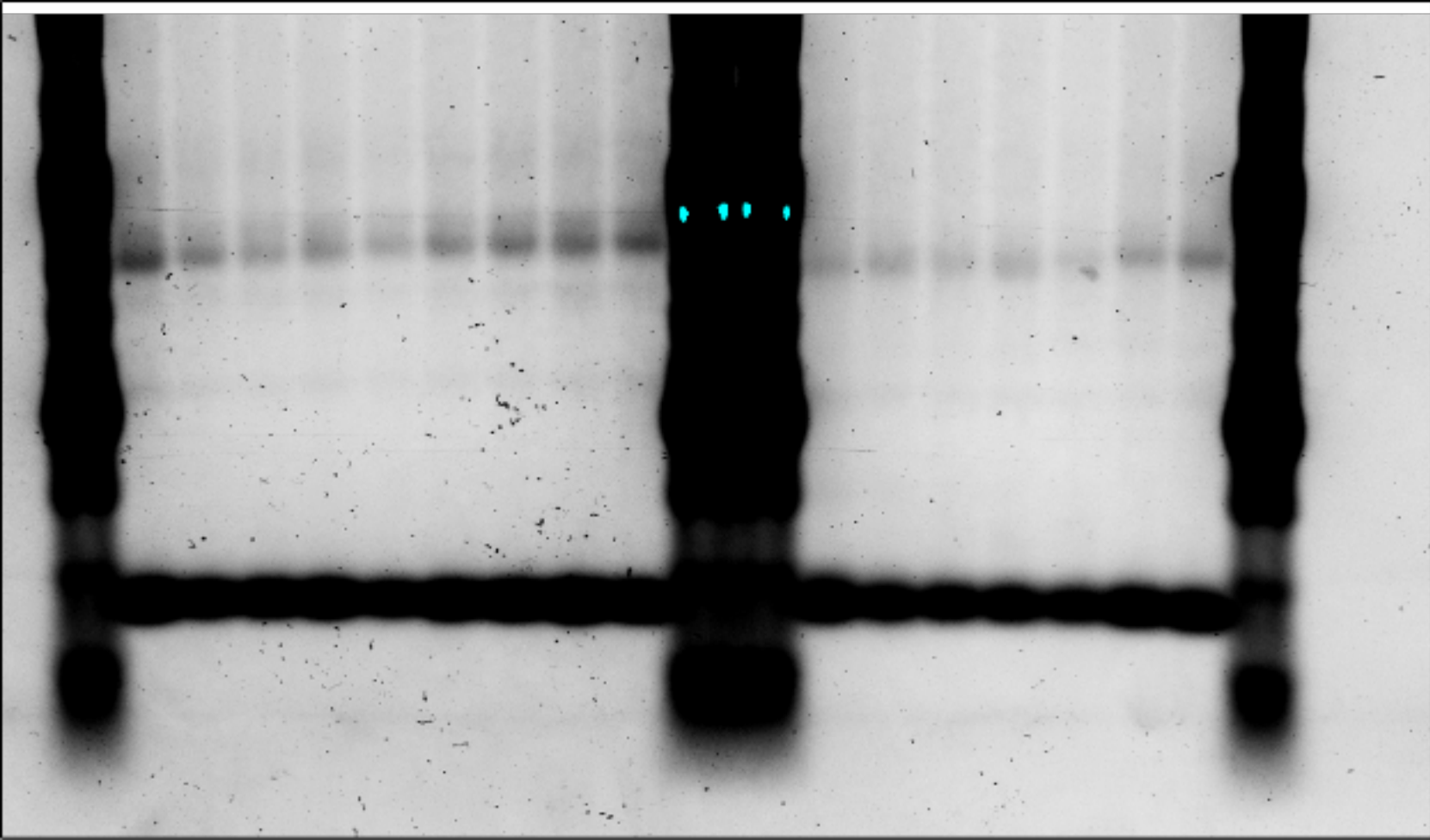

Supplement: Supplementary file 16 — Unprocessed gel. [file 41588_2025_2172_MOESM16_ESM.pdf]

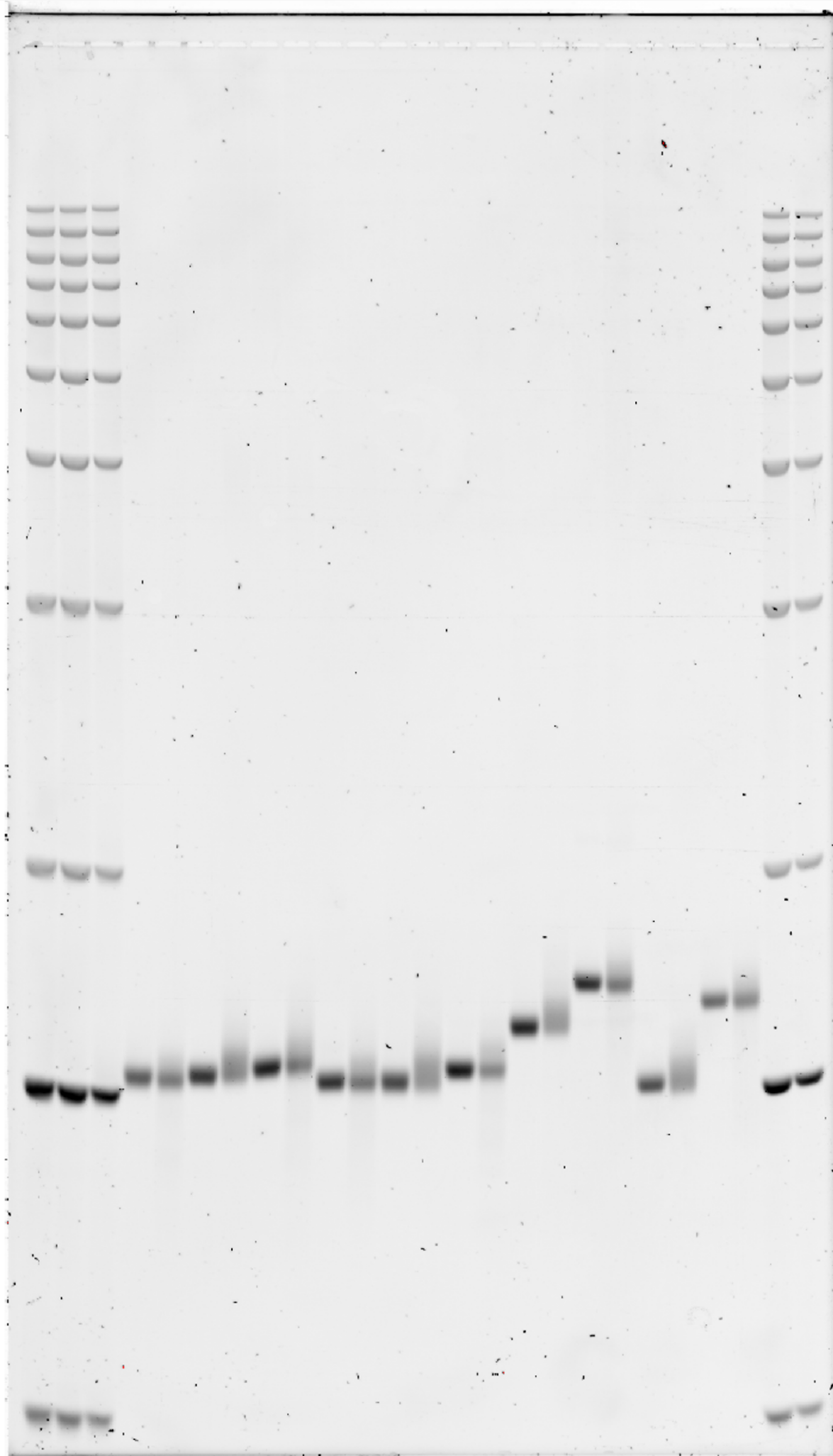

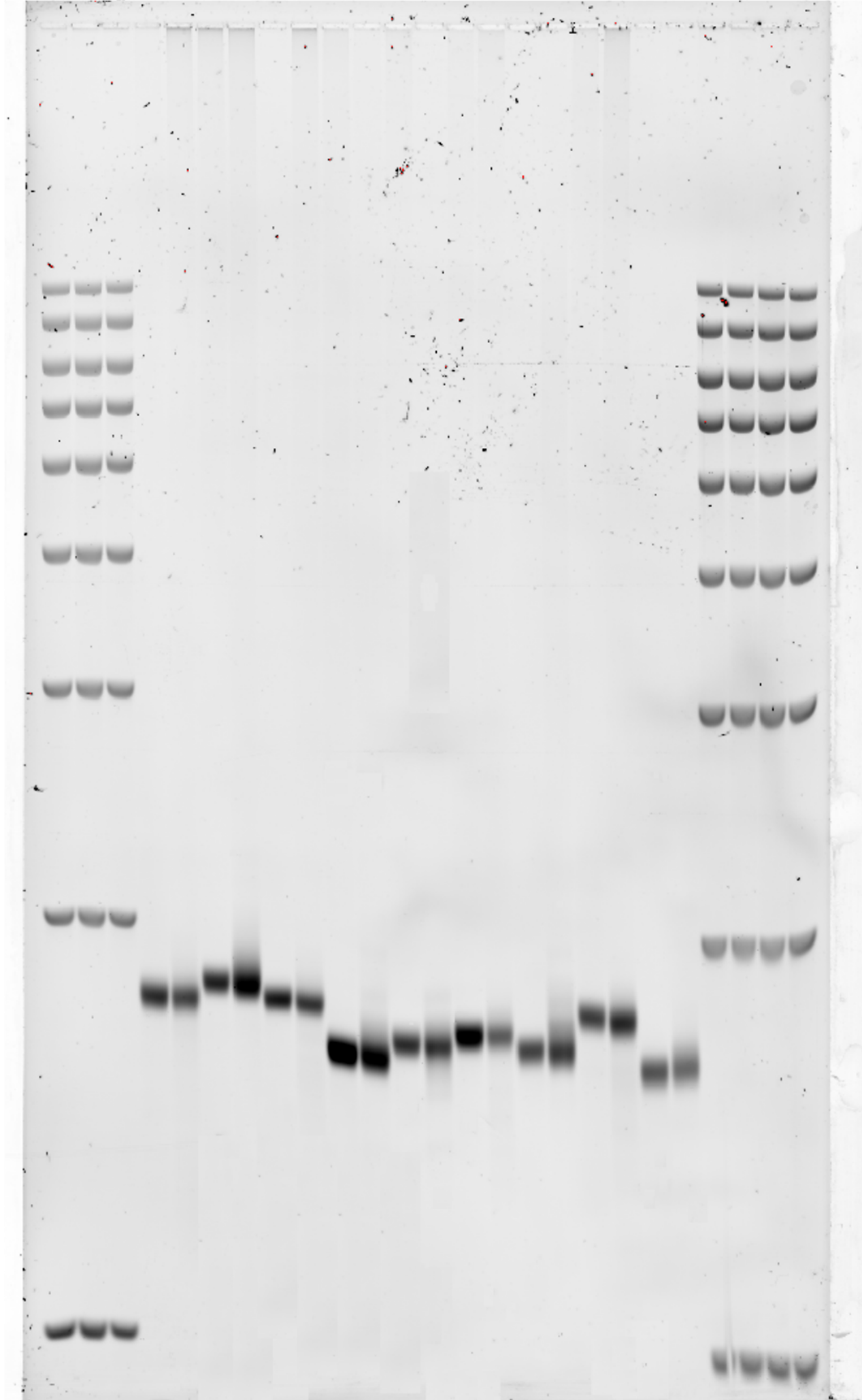

Supplement: Supplementary file 17 — Unprocessed gel. [file 41588_2025_2172_MOESM17_ESM.pdf]
